# o8G-modified circKIAA1797 promotes lung cancer development by inhibiting cuproptosis

**DOI:** 10.1186/s13046-025-03365-z

**Published:** 2025-04-02

**Authors:** Haotian Xu, Qingyun Zhao, Dunyu Cai, Xingcai Chen, Xiaodong Zhou, Yihong Gao, Jiaxi Wu, Shengyi Yuan, Deqing Li, Ruirui Zhang, Wenyi Peng, Gang Li, Aruo Nan

**Affiliations:** 1 School of Public Health, Guangxi Medical University, Nanning, 530021 China; 2 Guangxi Key Laboratory of Environment and Health Research, Guangxi Medical University, Nanning, 530021 China

**Keywords:** Lung cancer, circKIAA1797, o8G modification, Cuproptosis

## Abstract

**Background:**

Lung cancer is a serious threat to human life and health, but effective screening and treatment methods are lacking. Circular RNAs (circRNAs) have important biological functions and are closely related to tumour development. Some studies have shown that the 8-oxo-7,8-dihydroguanosine (o8G) modification plays a key role in the disease process, but the effect of the o8G modification on circRNAs has not been elucidated. Moreover, cuproptosis is a novel mode of cell death in which copper ions directly promote protein aggregation and the disruption of cellular metabolic pathways. The present study revealed that the o8G modification of circKIAA1797 occurs and promotes lung cancer development by inhibiting cuproptosis, which provides new perspectives for epitranscriptomic studies and the development of novel therapeutic approaches for lung cancer.

**Methods:**

circRNA differential expression profiles in lung cancer were revealed via RNA high-throughput sequencing, and circKIAA1797 expression in lung cancer cell lines and tissues was detected using qPCR. Experiments such as o8G RNA immunoprecipitation (o8G RIP) and crosslinking immunoprecipitation (CLIP) were performed to explore the presence of o8G on circKIAA1797. The regulation of circKIAA1797 by the o8G reader Y-box binding protein 1 (YBX1) was explored using nuclear–cytoplasmic fractionation, actinomycin D (Act D) stability experiments and other experiments. circKIAA1797 silencing and overexpression systems were constructed for in vivo and in vitro experiments to study the role of circKIAA1797 in lung cancer development. Tagged RNA affinity purification (TRAP), RNA immunoprecipitation (RIP), coimmunoprecipitation (Co-IP), and immunofluorescence (IF) staining were subsequently conducted to reveal the molecular mechanism by which circKIAA1797 regulates cuproptosis and promotes lung cancer development.

**Results:**

This study is the first to reveal the presence of o8G on circKIAA1797 and that YBX1 is a reader that recognises ROS-induced circKIAA1797 o8G modifications and increases the stability and cytoplasmic expression of circKIAA1797. circKIAA1797, which is associated with the tumour stage and prognosis, has been shown to significantly promote the biological function of lung cancer development both in vivo and in vitro. This study revealed that circKIAA1797 inhibits intracellular cuproptosis by binding to the ferredoxin 1 (FDX1) mRNA, decreasing FDX1 mRNA stability, inhibiting FDX1 expression, and binding to the signal transducer and activator of transcription 1 (STAT1) protein and inhibiting lipoyltransferase 1 (LIPT1) transcription; moreover, circKIAA1797 promotes the closure of the mitochondrial permeability transition pore (mPTP), inhibits cuproptosis, and ultimately promotes lung cancer development.

**Conclusions:**

This study revealed the presence of the o8G modification in circKIAA1797, which plays an important role in the development of lung cancer. circKIAA1797 can inhibit cuproptosis by inhibiting key cuproptosis proteins and promoting mPTP closure, ultimately promoting the development of lung cancer. This study provides not only a new theoretical basis for an in-depth understanding of the molecular mechanisms of lung cancer development but also a potential target for lung cancer treatment.

**Supplementary Information:**

The online version contains supplementary material available at 10.1186/s13046-025-03365-z.

## Introduction

According to the latest statistics from the International Agency for Research on Cancer (IARC) of the World Health Organization, an estimated 2.48 million new cases of lung cancer were diagnosed in 2022, accounting for 12.4% of the total number of new cancer cases in the world, and it has re-emerged as the most common cancer worldwide [1], thus posing a serious threat to human life and health [2]. Currently, cancer treatments mainly include surgery, radiotherapy, chemotherapy, targeted therapy, immunotherapy, etc [3–5]. Depending on the specific situation, patients need to receive a combination of treatments to achieve the best therapeutic effect. In recent years, the rapid development of targeted therapy has brought new hope for cancer treatment, but difficulties persist, such as a lack of target genes and gene mutations [6–8]. Therefore, identifying new targets that are more conservative and specific is particularly important.

Circular RNAs (circRNAs) are generated mainly by the reverse splicing of precursor RNAs; do not possess 5’ caps or 3’ polyadenylated tails; and have a closed-loop structure [9], which is highly stable, tissue specific, and widely expressed [10]. As an emerging research subject, circRNAs show great potential and advantages in cancer diagnosis and treatment [11, 12]. circEMB, a therapeutic target for osteosarcoma (OSA), promotes the development of OSA through the miR-3184-5p/EGFR pathway [13]. circATP8A1, a potential prognostic biomarker and therapeutic target for gastric cancer, induces macrophage M2 polarisation and gastric cancer progression through the circATP8A1/miR-1-3p/STAT6 axis [14]. In addition, circRNAs regulate cellular, physiological and pathological processes via different mechanisms [15]. circRNAs are involved in regulating protein function and can directly interact with proteins and affect their stability, activity, etc. Exosomal circLPAR1 directly binds to eIF3h, competitively inhibits the METTL3-eIF3h interaction, and suppresses the translation of the oncogene BRD4 [16]. circRNAs also directly bind RNA and affect RNA stability and activity. circRERE acts as a competitive endogenous RNA that adsorbs miR-6837-3p to upregulate MAVS expression, thereby activating the type I interferon signalling pathway and promoting antitumour immunity [17]. Finally, circRNAs can also affect the transcriptional activity of genes by interacting with RNA polymerase II or specific transcription factors. circCAPRIN1 directly binds to STAT2, activates the transcription of ACC1, regulates lipid metabolism, and promotes colorectal cancer (CRC) tumorigenesis [18].

Despite increasing research on the roles of circRNAs in tumorigenesis and progression, recent studies have focused on the regulation of downstream genes and signalling pathways by circRNAs [19–21], but the upstream molecular mechanisms of circRNAs are still poorly understood. The expansion of research on RNA modifications has provided new research ideas for elucidating the mechanisms of changes in circRNA expression and for functional studies of circRNAs in malignant tumours. More than 170 chemical modifications, such as 5-methylcytosine (m5C), N6-methyladenine (m6A), and 8-oxoguanine, have been found on RNA [22, 23]. Recent studies of circRNAs have focused mainly on m6A methylation, whereas few studies have focused on other modifications [24]. Generally, an imbalance exists between reactive oxygen species (ROS) levels and the concentration of the reducing substance glutathione (GSH) in tumour cells; GSH generally balances ROS, but its concentration is not high enough in tumour cells to prevent ROS accumulation [25]. This increased level of ROS is a prerequisite for the occurrence of the 8-oxo-7,8-dihydroguanine (8-oxoG) or 8-oxo-7,8-dihydroguanosine (o8G) modification. Since RNA is a relatively unstable and transient intermediate, guanine residues in RNA are more susceptible to the o8G modification than guanine residues in DNA. o8G, an epitranscriptional modification, alters the regulation of RNA‒RNA interactions in a “redox-dependent” manner. At the posttranscriptional level, inappropriate o8G-A base pairing affects RNA structure and function and leads to translation errors that reduce RNA translational activity, thereby affecting protein synthesis and cellular function [26]. miRNAs (miR-124, let-7, and miR-1) have o8G modifications that affect their stability, target specificity, and ability to regulate gene expression [27, 28], but the o8G modification of circRNAs has not been reported.

Numerous studies have shown that circRNAs can participate in cellular events such as apoptosis [29], ferroptosis [30, 31], autophagy [32], or lipid metabolism [33] in various ways to regulate cellular functions and play extremely critical roles in tumour formation and development. Cuproptosis is a new type of cell death event that is distinguished from apoptosis, necrosis, and autophagy and is triggered by the accumulation of excessive copper ions [34]. Research on cuproptosis is still in the preliminary stage, and the mechanism of cuproptosis involves several processes [35, 36]. First, in terms of oxidative stress, excess copper ions promote ROS production, increase oxidative stress, damage the cell structure and function, and ultimately lead to cell death [37]. Second, copper can bind to a variety of proteins in the cell, altering their structures and functions and thereby interfering with normal signal transmission and metabolic processes in the cell [38]. In terms of mitochondrial damage, the accumulation of copper ions can also directly damage mitochondria, affecting their energy metabolism, leading to the loss of the mitochondrial membrane potential and promoting programmed cell death [39]. Finally, in terms of DNA damage, excessive copper ions can also directly or indirectly induce ROS generation, leading to DNA damage and triggering the cellular DNA damage response mechanism, which in turn triggers cell cycle arrest or cell death [40]. An increasing number of studies have shown the therapeutic value of cuproptosis in various cancers, such as lung, liver, and breast cancers [41]; however, methods for regulating cuproptosis are lacking [42]. circRNA-related studies will help further elucidate the molecular mechanism of cuproptosis and provide new insights into the development of more effective tumour therapeutic regimens.

In this study, we found for the first time that o8G modifications occur on circRNAs and that YBX1 can act as an o8G reader to increase circKIAA1797 expression. Through in vivo and in vitro functional experiments, o8G-modified circKIAA1797 was shown to significantly promote the development of lung cancer. An exploration of the molecular mechanism revealed that circKIAA1797 could bind to the ferredoxin 1 (FDX1) mRNA, reduce FDX1 mRNA stability, inhibit FDX1 expression and bind to the signal transducer and activator of transcription 1 (STAT1) protein and inhibit lipoyltransferase 1 (LIPT1) transcription; moreover, circKIAA1797 could promote the mitochondrial permeability transition pore (mPTP) closure, which in turn inhibited cuproptosis and ultimately promoted the development of lung cancer. In this study, we elucidated the molecular mechanism by which circKIAA1797 inhibits cuproptosis from an epigenetic perspective, which provides a new approach for identifying therapeutic targets for lung cancer.

## Methods

### Cell culture

Human normal bronchial mucosal epithelial cells (BEAS-2B) were purchased from the American Typical Culture Collection (ATCC), lung cancer cell lines (H460, H226, H2170, H1299, and A549) were purchased from the Typical Culture Collection Centre of the Chinese Academy of Sciences (Shanghai, China), and human embryonic renal cells (293T) were purchased from Guangzhou Cellcook Blot ech Co. BEAS-2B cells were cultured in bronchial epithelial cell-specific growth medium (BEGM) (Lonza, CC-3171). H460, H226, H2170 and H1299 cells were cultured in RPMI-1640 medium (Servicebio, G4530), A549 cells were cultured in Ham’s F-12 K medium (Servicebio, G4560), and 293T cells were cultured in DMEM (Servicebio, G4510); all of these types of media contained 10% foetal bovine serum (Gibco, 10099141C) and 1% penicillin‒streptomycin (Servicebio, G4003). All the cells were cultured in a cell culture incubator at 37 °C with 5% CO_2_.

### Patient sample collection

The 35 pairs of paraneoplastic and lung cancer tissue samples used in this study were obtained from the First Affiliated Hospital of Guangxi Medical University. All the subjects who provided samples signed an informed consent form. All studies followed the guidelines of the Declaration of Helsinki and were formally approved by the Ethics Committee of the First Affiliated Hospital of Guangxi Medical University (No. 2022-KY-E-(289)).

### Animal model experiments

Four-week-old, female, specific pathogen-free (SPF)-grade nude mice were purchased from Guangxi Medical University Laboratory Animal Centre for this study. The animal experiments in this study followed internationally accepted guidelines, and the experimental animal management complied with the basic operational requirements for experimental animals. All animal experiments were approved by the Experimental Animal Ethics Committee of Guangxi Medical University (No. 202311216).

### Construction of the circKIAA1797 expression vector

The full-length sequence of circKIAA1797 was amplified from tool cell (293T) cDNA using specific primers. The PCR product was gel-purified and cloned between the BamHI and EcoRI restriction enzyme cleavage sites of the pcircRNA 2.2 hsa vector (BersinBio, Guangzhou, China). The vector contains flanking intron sequences with complementary Alu elements that promote RNA cyclisation. The constructed plasmid was verified by Sanger sequencing.

### Acquisition of other plasmids

LITP1 and Bcl2 overexpression plasmids and the corresponding empty vector (pcDNA3.1) were purchased from Fenghui Biotechnology Co (Changsha, China). The FDX1 overexpression plasmid was constructed by inserting the full-length FDX1 cDNA into the pcDNA3.1 vector (BersinBio, Guangzhou, China). All plasmids were extracted using a plasmid extraction kit (Biomiga, BW-PD1211-03) according to the manufacturer’s instructions.

### Stably transfected cell construction

A stock solution of lentivirus stably expressing the circKIAA1797-silencing construct was purchased from Shanghai Genechem Co. Ltd. (Shanghai, China). The general steps for extracting the circKIAA1797 shRNA virus were as follows: the synthesised oligonucleotides, according to the circKIAA1797 siRNA sequences, were annealed and cloned and inserted into the GV112 lentiviral vector (GeneChem, Shanghai, China) between the AgeI and EcoRI restriction sites under the control of the human U6 promoter. The constructed plasmids were verified by Sanger sequencing. The lentiviral particles were produced by cotransfecting 293T cells with the recombinant GV112 vector and two helper plasmids (pHelper 1.0 and pHelper 2.0) using GeneChem transfection reagent. The viral supernatant was collected at 48–72 h posttransfection, concentrated by ultracentrifugation, and titrated using the qPCR method. The viral stock solution was added dropwise to the culture media of 60% confluent A549 and H1299 cells to reach a viral load of 1 × 10^8^ (MOI = 100), and the cells were cultured in a cell culture incubator for 72 h. Fluorescence signals were assessed by fluorescence microscopy (AMG EVOS, Mill Creek, USA) for the evaluation of fluorescence signals. Single colonies of stably transfected cells were selected by screening.

### Reverse transcription and qPCR

Total RNA was isolated from cell samples using TRIzol reagent (Invitrogen, 15596018) and quantified using a Nanodrop One Spectrophotometer (Thermo Fisher Scientific, ND-ONEC-W). The GoScript™ Reverse Transcription System Kit (Promega, A5002) was used for circRNA and mRNA reverse transcription with GoTaq^®^ qPCR Master Mix (Promega, A6001) and an Applied Biosystems™ QuantStudio™ 7 Flex Real-Time PCR System (Thermo Fisher Scientific). The results of the target RNA were subsequently normalised to those of the internal control and presented as 2^−△△CT^ values relative to those of the control sample. β-Actin was used as an internal reference for DNA, and GAPDH was used as an internal reference for circRNAs and mRNAs (except for nuclear–cytoplasmic fractionation, CLIP-qPCR, and RNase R digestion experiments). Please see Supplementary Table 1 for the sequences of the primers used in this study.

### Cell transfection

Overexpression plasmids were transfected using a Lipofectamine^®^ 3000 kit (Invitrogen, L3000015). A total of 3 × 10^5^ cells were inoculated into 6-well plates and cultured in an incubator. When the cell confluence reached 80%, 2.5 µg of plasmid was added to each well of a 6-well plate for transfection. The subsequent experiments were performed after the cells were incubated for 48 h.

### RNA stability assay

A total of 3 × 10^5^ cells were inoculated in 6-well plates and cultured for 24 h, and when the cell confluence reached 90%, actinomycin D (Act D: 2 µg/ml) was added; the total RNA was extracted from the cells after treatment for 0, 4, 8 and 12 h, and gene expression was measured via RT‒qPCR.

### Fluorescence in situ hybridisation (FISH)

The subcellular localisation of circKIAA1797 was analysed using a FISH kit (RiboBio, C10910). A total of 1 × 10^4^ cells were added to each of the two slides, and the cells were incubated for 12 h to achieve 40% confluence. After fixation, permeabilization, and dehydration, the cells on one slide were treated with RNase R for 15 min as the treatment group, and the cells on the other slide were not treated with RNase R as the control group. The cells on the two slides were then hybridised with a circKIAA1797-specific FISH probe overnight at 42 °C, followed by washes with sodium citrate (SSC) and staining with 4’,6-diamidino-2-phenylindole (DAPI). The fluorescence signals were captured with a laser confocal microscope (Zeiss, LSM800).

### Nuclear–cytoplasmic fractionation

Nuclear–cytoplasmic fractionation assays were performed using a PARIS™ kit (Invitrogen, AM1921). A total of 2.5 × 10^6^ cells were collected, and then the nuclear and cytoplasmic RNA were isolated using kits. β-Actin or GAPDH was used as the cytoplasmic marker, and U6 was used as the nuclear marker. RT‒qPCR was performed to assess the distribution of circKIAA1797 in the nucleus and cytoplasm.

### RNase R digestion assay

After total RNA was collected, 1 µg of total RNA was placed in each of two PCR tubes (200 µl), which were labelled the treatment group and the control group. Three units of RNase R was added to the treatment group, while no RNase R was added to the control group. Both groups were placed in a 37 °C water bath for 10 min, after which RT‒qPCR was performed to detect the expression of circKIAA1797. Due to the RNase R treatment, the internal reference gene (linear) was digested, and the RNA samples from the two groups were obtained from the same tube, ensuring the homogeneity of the samples before treatment. Therefore, the CT values obtained by qPCR were not normalised to any internal reference, and only the difference in CT values between the control group and the treatment group was compared. Cellular gDNA was extracted using a gDNA extraction kit (Invitrogen, K1820), washed, purified and processed for quantification. Convergent primers and divergent primers specific for circKIAA1797 were designed. After RNase R treatment, the gDNA samples were subjected to PCR amplification, followed by agarose gel electrophoresis.

### Differential analysis of oligo dT primer and random primer reverse transcription products

After total cellular RNA was collected, reverse transcription was performed with the GoScript™ Reverse Transcription System Kit (Promega, A5002) using oligo(dT) primers and random primers, and qPCR was subsequently performed to detect circKIAA1797 expression in the reverse transcription products.

### 8-oxo-7,8-dihydroguanosine RNA Immunoprecipitation (o8G RIP)

The experiments were carried out using a methylated RNA immunoprecipitation kit (IEMed, K368). After 2 × 10^7^ cells were collected and total RNA was extracted, an o8G-specific antibody (Sigma, MAB3560) was conjugated to Protein A/G magnetic beads to pull down the target RNA from the total RNA, which was eluted and purified according to the instructions. RT‒qPCR experiments were subsequently performed, and the PCR products were analysed by agarose gel electrophoresis. For the normalisation of circKIAA1797 expression levels in o8G RIP–qPCR experiments, we measured GAPDH levels in total RNA input samples collected prior to immunoprecipitation rather than in IP or IgG samples. The relative enrichment of circKIAA1797 in the o8G RIP samples was calculated using the 2^−ΔΔCT^ method, where ΔCT represents the difference between the CT values of circKIAA1797 and the input GAPDH, followed by normalisation to the input group.

### Crosslinking and Immunoprecipitation (CLIP)

The experiments were performed using a CLIP kit (IEMed, K319). According to the circKIAA1797 sequence, 50 bp primers were designed, and 2 × 10^7^ cells were collected and crosslinked under a UV lamp at 254 nm and 0.15 J/cm^2^ for 10 min. A specific antibody was used to bind to Protein A/G magnetic beads and pull down the target RNAs, which were subsequently digested and purified. Reverse transcription was performed via the add-tail method, and qPCR experiments were conducted for circKIAA1797 via a qPCR assay with the protein binding region. For the normalisation of circKIAA1797 expression levels in CLIP‒qPCR experiments, we measured U6 levels in total RNA input samples collected prior to immunoprecipitation rather than in IP or IgG samples. The relative enrichment of circKIAA1797 in the CLIP samples was calculated using the 2^−ΔΔCT^ method, where ΔCT represents the difference between the CT values of circKIAA1797 and the input U6, followed by normalisation to the input group. The antibodies used in this study were as follows: anti-8-oxoguanine (Sigma, MAB3560), anti-YBX1 (Proteintech, 20339-1-AP), and anti-STAT1 (Proteintech, 10144-2-AP).

### ROS detection assay

The ROS levels in the cells were detected using a reactive oxygen species detection kit (Beyotime, S0033S). A total of 2 × 10^5^ cells were inoculated in 6-well plates and cultured for 24 h. After treatment with NAC or H_2_O_2_ for 2 h, 1 ml of DCFH-DA at a final concentration of 10 µmol/l was added to each well and incubated for 25 min at 37 °C in a cell culture incubator. The cells were subsequently washed with serum-free medium three times, the cells were collected, and the levels of ROS in the cells were detected using a flow cytometer (Beckman Coulter, Miami, USA).

### Immunofluorescence (IF) staining

A total of 1 × 10^4^ cells were inoculated on cell culture slides, cultured for 12 h until the cell density reached 60%, fixed with formaldehyde for 15 min, washed three times with 1× PBS, incubated with the primary antibody overnight at 4°C on a shaker, incubated with the corresponding fluorescent dye-conjugated secondary antibody, and incubated for 2 h at room temperature in the dark; the nuclei were stained with DAPI. Fluorescence was captured using a laser confocal microscope (Zeiss, LSM800) for analysis. The antibodies used in this study were as follows: anti-DLAT (Proteintech, 1:2000, 68303-1-Ig), anti-o8G (Abcam, 1:100, ab206461), anti-FDX1 (Proteintech, 1:500, 12592-1-AP), anti-LIPT1 (Biorbyt, 1:100, orb513404), anti-rabbit IgG (Cell Signaling Technology, 1:1000, 8890S) and anti-mouse IgG (Cell Signaling Technology, 1:1000, 4408S).

### RNA binding protein Immunoprecipitation (RIP)

The RIP experiments were performed using a RIP kit (IEMed, K303). A total of 2 × 10^7^ cells were collected, antibody–Protein A/G magnetic bead complexes were added to the lysed cell mixture according to the instructions, incubated for 1 h at room temperature on a shaker, and thermally eluted at 75 °C for 10 min. The purified RNA products were collected, and RT‒qPCR was performed to assess the enrichment of circKIAA1797. For the normalisation of circKIAA1797 expression levels in RIP–qPCR experiments, we measured GAPDH levels in total RNA input samples collected prior to immunoprecipitation rather than in IP or IgG samples. The relative enrichment of circKIAA1797 in the RIP samples was calculated using the 2^−ΔΔCT^ method, where ΔCT represents the difference between the CT values of circKIAA1797 and the input GAPDH, followed by normalisation to the input group. The antibodies used in this study were as follows: anti-YBX1 (Proteintech, 20339-1-AP), anti-STAT1 (Proteintech, 10144-2-AP), and anti-ANT2 (SAB, 29214).

### Cell viability

The experiments were performed using a Cell Counting Kit-8 (Dojindo, CK04). A total of 4 × 10^3^ cells were inoculated in 96-well plates and cultured for 24 h. Transfection was performed when the cell density reached 50%. Forty-eight hours after transfection, the CCK-8 reagent was mixed with the culture medium at a ratio of 1:10, and the absorbance value was detected at 450 nm after 1.5 h of incubation in a 37 °C incubator.

### Cell cycle analysis

The experiments were performed using a cell cycle detection kit (KeyGen Biotech, KGA512). A total of 1.5 × 10^5^ cells were inoculated into 6-well plates and cultured for 24 h. Transfection was performed when the cell density reached 40%. Forty-eight hours later, the cells were collected, washed with 1× PBS, fixed with 70% ethanol overnight, digested with RNase A at 37 °C for 30 min, and counted using a CytoFLEX flow cytometer (Beckman Coulter, Miami, USA) after PI staining.

### Apoptosis analysis

The experiments were conducted using the Annexin V-FITC/PI Double Staining Apoptosis Detection Kit (KeyGen Biotech, KGA107). The cell seeding protocol and transfection conditions were the same as those used for the cell cycle experiments. Forty-eight hours after transfection, the cells were collected, washed with 1× PBS, and counted using a CytoFLEX flow cytometer (Beckman Coulter, Miami, USA) after double staining with FITC and PI.

### 5-Ethynyl-2-deoxyuridine (EdU) incorporation assay

The experiments were performed using a Cell-Light EdU Apollo 567 In Vitro Kit (RiboBio, C10310-1). A total of 4 × 10^3^ cells were inoculated in 96-well plates and cultured for 24 h. Transfection was performed when the cell density reached 40%. Forty-eight hours after transfection, the EdU solution was diluted at a ratio of 1:1000, 100 µl was added to each well for EdU labelling, and cell fixation, Apollo staining, and DNA staining were subsequently performed according to the instructions. The cells were imaged using an EVOS^®^ FL automated imaging system, and the cell proliferation was analysed with ImageJ software.

### Transwell migration assay

A total of 1.5 × 10^5^ cells were inoculated into 6-well plates and cultured for 24 h, and transfection was performed when the cell density reached 40%. After the upper migration chamber (Corning, 3422) and the lower migration chamber were equilibrated with 400 µl and 700 µl of serum-free/double-antibody-free medium, respectively, for 2 h, 400 µl of the cell suspension (containing 8 × 10^3^ cells) was added to the upper chamber. After 24 h of incubation, the cells were fixed with 4% paraformaldehyde for 15 min and stained with crystal violet; the cells remaining in the upper chamber were removed, and the cells that migrated to the lower chamber were imaged using an EVOS^®^ FL automated imaging system and analysed with ImageJ software.

### Wound healing assay

A total of 1.5 × 10^5^ cells were inoculated into 6-well plates and cultured for 24 h. Transfection was performed when the cell density reached 40%. Forty-eight hours after transfection, a pipette tip was used to make straight and uniform scratches (presented in the form of a 9-cell grid) on the cells at the bottom of the 6-well plate. At 0 h and 48 h, the cells were imaged using a microscope (Olympus, Tokyo, Japan).

### Immunohistochemistry (IHC)

Animal tissues were fixed, embedded in paraffin and sectioned. The sections were then incubated with specific antibodies and stained with haematoxylin (Servicebio, G1077). Images were acquired under a light microscope (Leica, Mannheim, Germany) at 200× and 400× magnifications. The antibodies used in this study were as follows: anti-Bcl2 (Affinity, 1:100, AF6139), anti-cyclin D1 (Servicebio, 1:1000, GB111372), anti-FDX1 (Proteintech, 1:100, 12592-1-AP), anti-Ki67 (Servicebio, 1:1000, GB111499), anti-LIPT1 (Biorbyt, 1:1000, orb451223), and anti-RhoA (Servicebio, 1:1000, GB115177).

### Reduced glutathione (GSH) assay

The experiments were performed using a reduced glutathione (GSH) assay kit (Nanjing Jiancheng Bioengineering Institute, A006-2-1). A total of 1 × 10^5^ cells were inoculated in 6-well plates and cultured for 24 h. The cell density was allowed to reach 40% before transfection. Forty-eight hours after transfection, the cells were collected and homogenized; blank wells, standard wells and assay wells were established according to the instructions; the assay reagents were added in order; the samples were incubated for 5 min; and the absorbance at 405 nm was detected.

### Tagged RNA affinity purification (TRAP)

The experiments were performed using a TRAP kit (BersinBio, Bes5106). The GST-MS2 fusion expression vector and circKIAA1797-MS2 stem‒loop structure tandem repeat vector used for the experiments were constructed by IEMed Biomedical Technology (Guangzhou, China) [43, 44]. For TRAP experiments, cells were seeded in two 15 cm culture dishes (2 × 10^7^ cells per dish) and cotransfected with MS2-circKIAA1797 and MS2-GST plasmids at a ratio of 2:1 (12 µg:6 µg per dish). Forty-eight hours after transfection, the cells were washed twice with PBS and lysed in 1.7 ml of lysis buffer supplemented with protease inhibitors, RNase inhibitor, and DTT. The cell lysates were subsequently centrifuged at 10,000×g for 15 min at 4℃. The supernatant was incubated with prewashed glutathione (GSH) magnetic beads for 3 h at 4℃ with gentle rotation. After washes with NT2 buffer and DNase I treatment, the RNA‒protein complexes were eluted. For the protein analysis, the bound proteins were eluted for 2 h at 37℃ with protein elution buffer containing DTT and then silver stained. LC‒MS was commissioned to IEMed Biomedical Technology (Guangzhou, China) for testing.

### Western blot

Total cellular proteins were obtained by lysing cells in homemade cell lysis buffer (1 M Tris-HCl [pH 7.4], 10% SDS and 100 mM Na_3_VO_4_). The samples were crushed using an ultrasonic cell crusher (Ningbo Scientz Biotechnology, Scientz-IID). The protein concentration was determined with the Pierce™ BCA Protein Assay Kit (Thermo Fisher Scientific, 23227). Proteins in the samples were separated via sodium dodecyl sulfate‒polyacrylamide gel electrophoresis (SDS‒PAGE) on 8‒15% gels and transferred to polyvinylidene difluoride (PVDF) membranes after 40–120 min at a constant current of 200 mA. The membranes were blocked for 1 h with 5% skim milk (diluted in TBST), and the membranes were incubated with primary antibodies overnight at 4 °C on a shaker. Subsequently, signals were developed using a chemiluminescence imager (ChemiScope S6), and protein grayscale values were analysed using ImageJ. The antibodies used in this experiment were as follows: anti-FDX1 (Proteintech, 1:500, 12592-1-AP), anti-LIPT1 (Biorbyt, 1:400, orb513404), anti-DLAT (Proteintech, 1:30000, 68303-1-Ig), anti-LIAS (Proteintech, 1:1000, 11577-1-AP), anti-TOM20 (Proteintech, 1:10000, 66777-1-lg), anti-lipoic acid (Abcam, 1:500, ab58724), anti-STAT1 (Proteintech, 1:2000, 10144-2-AP), anti-ANT2 (SAB, 1:1000, 29214), anti-BAX (Boster, 1:1000, BA0315-2), anti-Bcl2 (Affinity, 1:1000, AF6139), anti-rabbit IgG (Cell Signaling Technology, 1:5000, 8890S), and anti-mouse IgG (Cell Signaling Technology, 1:5000, 4408S).

### Chromatin Immunoprecipitation (ChIP)

The experiments were performed using a ChIP detection kit (BersinBio, Bes5001). A total of 4 × 10^7^ cells were collected and crosslinked with 1% formaldehyde for 10 min at room temperature. The nuclei were lysed by ultrasonication; STAT1-specific antibodies (Proteintech, 10144-2-AP) were added to form antibody‒magnetic bead‒DNA complexes; the DNA was enriched by elution, decrosslinking, and purification; and the enrichment efficiency was detected by qPCR. For normalisation of circKIAA1797 expression levels in ChIP‒qPCR experiments, we measured GAPDH levels in total RNA input samples collected prior to immunoprecipitation rather than in IP or IgG samples. The relative enrichment of circKIAA1797 in the IP samples was calculated using the 2^−ΔΔCT^ method, where ΔCT represents the difference between the CT values of circKIAA1797 and the input GAPDH, followed by normalisation to the input group.

### Chromatin isolation by RNA purification (ChIRP)

The experiments were performed using a ChIRP assay kit (BersinBio, Bes5104). A total of 8 × 10^7^ cells were collected and subjected to crosslinking with 1% formaldehyde at room temperature for 10 min. The samples were subsequently lysed and homogenised by ultrasonication, and the homogenised samples were mixed with a biotin-labelled circKIAA1797 complementary probe and incubated for 3 h. Magnetic bead–antibody complexes were added; RNA and DNA were enriched by incubation, elution and purification; and the enrichment efficiency was assessed by qPCR. For the normalisation of circKIAA1797 expression levels in ChIRP-qPCR experiments, we measured GAPDH levels in total RNA input samples collected prior to immunoprecipitation rather than in circRNA or lacZ samples. The relative enrichment of circKIAA1797 in the ChIRP samples was calculated using the 2^−ΔΔCT^ method, where ΔCT represents the difference between the CT values of circKIAA1797 and the input GAPDH, followed by normalisation to the input group.

### Coimmunoprecipitation (Co-IP) assay

The experiments were performed using a Co-IP kit (IEMed, K235). A total of 2 × 10^7^ cells were collected, crosslinked with 1% formaldehyde at room temperature for 10 min, heat denatured at 95 °C for 15 min, and cooled naturally to room temperature in a metal bath. Antibody–Protein A/G magnetic beads were added to enrich the target proteins, which were subsequently eluted and purified, after which the enrichment efficiency was detected by Western blot. The antibodies used in this experiment were as follows: anti-FDX1 (Proteintech, 12592-1-AP), anti-LIPT1 (Biorbyt, orb513404), and anti-lipoic acid (Abcam, ab58724).

### JC-1 apoptosis detection

The experiments were performed using a JC-1 Apoptosis Detection Kit (KeyGen Biotech, KGA604). A total of 1 × 10^5^ cells were inoculated in 6-well plates and cultured for 24 h. The cell density was allowed to reach 30% before transfection. Forty-eight hours after transfection, JC-1 working solution was added according to the instructions, and the cells were incubated in a cell culture incubator for 20 min and washed twice. Then, fluorescence was observed by fluorescence microscopy and assessed via fluorescence zymography at 485 nm, and changes in the membrane potential were detected by flow cytometry.

### Determination of the intracellular copper content

Intracellular copper levels were measured using inductively coupled plasma‒mass spectrometry (ICP‒MS). After the cells were collected, we commissioned the ICP‒MS experiment to Shanghai Weipu Testing Technology Group Co., Ltd., for completion. A549 cells with stable transfection of circKIAA1797 NC, shRNA1, and shRNA2 were cultured until reaching 1.6 × 10⁸ cells per group. Cells were harvested by trypsinization, washed three times with ice-cold PBS, and collected by centrifugation at 1000×g for 5 min at 4℃. For sample digestion, cell pellets were transferred to 50 ml centrifuge tubes and digested with a mixture of 2 ml HNO_3_, 6 ml HCl, 1 ml HF, and 1 ml H_2_O_2_, then diluted to 15 ml with ultra-pure water (18.2 MΩ·cm). The mixture was heated until complete digestion was achieved (clear solution). The ICP-MS analysis was performed under the following conditions: RF power 1550 W, plasma gas flow 15 L/min, auxiliary gas flow 0.9 L/min, and carrier gas flow 0.95 L/min. The sampling depth was set at 8.0 mm, using a MicroMist concentric nebulizer with a spray chamber temperature of 2℃. The 63Cu isotope was monitored with 45Sc (10 ppb) as internal standard. Calibration standards (0, 0.5, 1, 5, 10, 50 and 100 ppb) were prepared by serial dilution of copper standard solution. Quality control samples were analyzed every 10 samples, maintaining recovery rates between 95 and 105% and relative standard deviation (RSD) below 5%. Total copper concentrations were normalized to cell number.

### Kyoto encyclopedia of genes and genomes (KEGG) and network of cancer genes (NCG) analyses

The KEGG analysis was performed using the R software (v.4.2.2) package clusterProfiler (v.4.5.0) through Hiplot Pro (https://hiplot.com.cn/), a comprehensive web service for biomedical data analysis and visualisation. The NCG analysis was performed using the R software (v.4.1.3) package “DOSE” (v4.0.0) through Hiplot Pro (https://hiplot.com.cn/). For the KEGG and NCG analyses of the differentially expressed circRNAs, we used the host genes of all the differentially expressed circRNAs (including those upregulated and downregulated) for analysis to ensure comprehensiveness.

### Receiver operating characteristic (ROC) curve analysis

The diagnostic value of circRNA expression was evaluated by constructing receiver operating characteristic (ROC) curves. ROC curves were generated with GraphPad Prism 9.0 (San Diego, USA) by plotting sensitivity against 1-specificity. The area under the ROC curve (AUC) with 95% confidence intervals was calculated to assess the diagnostic performance, where an AUC value of 1.0 indicates perfect discrimination and 0.5 indicates no discriminative value.

### Joint analysis of clinical data and TCGA database

We downloaded TCGA data (UCSC Xena names “TCGA Lung Adenocarcinoma (LUAD) (23 datasets)” and “TCGA Lung Squamous Cell Carcinoma (LUSC) (24 datasets)”) from the UCSC Xena website (https://xena.ucsc.edu/) [45]. We collated TCGA data with the demographic and clinical characteristics of the clinical data we collected, such as age, sex and smoking status. We used propensity score matching to reduce the impact of potential confounding factors. The propensity scores were calculated using logistic regression analysis, considering the aforementioned demographic and clinical characteristics [46, 47]. We performed 1:5 optimal pair matching. The matching was optimal in the sense that the sum of the absolute pairwise distances in the matched sample was as small as possible. The method functionally relies on the R package MatchIt. The advantages of optimal pair matching include that the matching order is not required to be specified and that extreme within-pair distances are unlikely to be large, unlike with nearest neighbour matching. However, as a subset selection method, optimal pair matching tends to perform similarly to nearest neighbour matching in that similar subsets of units are selected for matching. This matching process was performed using R software (version 4.2.2, R Foundation for Statistical Computing, Vienna, Austria). After matching was complete, we analysed the data using GraphPad Prism 9.0 (San Diego, USA).

### Statistical analyses

All the experiments performed in this study were repeated three times, and the experimental data are presented as the means ± standard deviations. The *t *test (for normally distributed data) or Wilcoxon rank sum test (for nonnormally distributed data) was used for comparisons between two sets of measurements. Pearson’s correlation analysis (for normally distributed data) or Spearman’s correlation analysis (for nonnormally distributed data) was performed to assess correlations between variables. The statistical analyses were conducted using SPSS 25.0 (IBM, Chicago, USA), and the graphs were generated using GraphPad Prism 9.0 (San Diego, USA). All tests were two-sided; *P* < 0.05 indicated a statistically significant difference. ns, not significant; *, *P* < 0.05.

## Results

### circKIAA1797 is significantly upregulated in lung cancer tissues

**Fig. 1:**
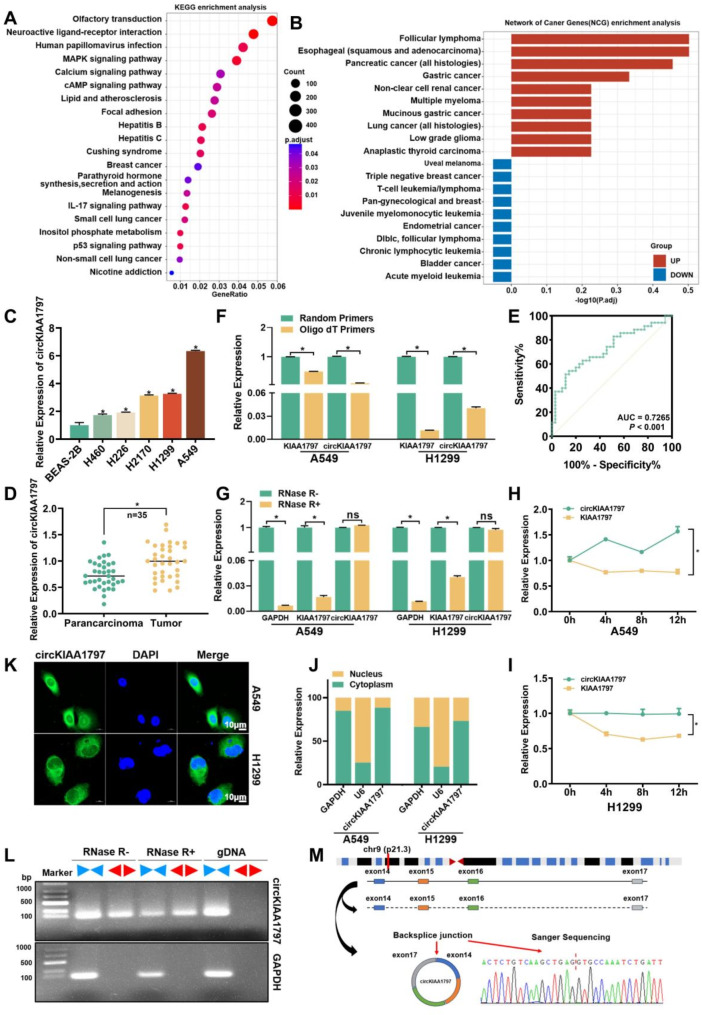
circKIAA1797 is significantly upregulated in lung cancer tissues and cells. (**A** and **B**) Bioinformatics analysis of circRNA high-throughput sequencing results, KEGG pathway analysis results and NCG enrichment analysis results. (**C**) Expression of circKIAA1797 in control cells (BEAS-2B) and lung cancer cell lines (H460, H226, H2170, H1299 and A549). (**D**) Expression of circKIAA1797 in 35 pairs of lung cancer tissues and paracancerous tissues. (**E**) Receiver operating characteristic (ROC) curve of circKIAA1797. (**F**) Total RNA from A549 and H1299 cells was reverse transcribed with random primers and oligo (dT) primers, respectively. qPCR was performed to compare the reverse transcription efficiency of KIAA1797 and circKIAA1797. (**G**) A549 and H1299 cellular RNA was treated with RNase R for 10 min. A qPCR assay was performed to compare circKIAA1797 expression with mRNA digestion. (**H** and **I**) A549 and H1299 cells were treated with Act D for 0, 4, 8, or 12 h. qPCR assays were performed to compare the half-life of circKIAA1797 with that of KIAA1797. (**J**) Results of the nuclear–cytoplasmic fractionation assay of A549 and H1299 cells. (**K**) Results of the FISH assay of A549 and H1299 cells. (**L**) A549 total RNA was divided into two groups: one group was not treated with RNase R, and one group was treated with RNase R. The gDNA of A549 cells was extracted at the same time. The samples from the three groups were amplified by PCR using divergent primers and convergent primers for circKIAA1797, and the amplified products were subjected to agarose gel electrophoresis. (**M**) circKIAA1797 gene location map and Sanger sequencing results

We identified circRNAs that play key roles in lung cancer development by analysing preconstructed circRNA high-throughput sequencing data (Data S1 [43, 44]). Kyoto Encyclopedia of Genes and Genomes (KEGG) pathway enrichment analysis and Network of Cancer Genes (NCG) enrichment analysis (Fig. 1A and B) of the differentially expressed circRNAs revealed that these differentially expressed circRNAs were closely associated with multiple important cancer-related pathways. The 10 circRNAs that were most significantly upregulated (Supplementary Fig. 1A) according to the sequencing results were subsequently examined in lung cancer cells via RT‒qPCR. circKIAA1797 was significantly upregulated in lung cancer cell lines (H460, H226, H2170, H1299, and A549) compared with BEAS-2B cells (Fig. 1C). Furthermore, circKIAA1797 was detected in 35 pairs of lung cancer tissue samples. The expression of circKIAA1797 was also significantly upregulated in lung cancer tissues compared with paracancerous tissues (Fig. 1D). A receiver operating characteristic (ROC) curve analysis was performed to assess the diagnostic efficacy of circKIAA1797 in a lung cancer screen. The area under the curve (AUC) was 0.7265, with a *P* value less than 0.001, indicating that circKIAA1797 has the potential to be a diagnostic marker for lung cancer (Fig. 1E). Moreover, an analysis of lung cancer population data revealed that the circKIAA1797 expression level was positively correlated with the smoking status and tumour stage (Supplementary Fig. 1B and 1C).

For a comprehensive understanding of circKIAA1797, we queried the UCSC Genome Browser website. We found that circKIAA1797 is an exonic circRNA formed by reverse splicing of exons 14–17 of the KIAA1797 pre-mRNA with a length of 465 nt. Based on the circKIAA1797 reverse splicing site, specific primers were designed, and qPCR and Sanger sequencing were performed to clarify the circKIAA1797 circularisation site (Fig. 1M). Since circRNAs usually do not contain poly(A) tails, qPCR was performed using oligo(dT) primers and random primers reverse transcription products. circKIAA1797 was less efficiently reverse transcribed than the linear KIAA1797 mRNA when oligo(dT) primers were used (Fig. 1F). Compared with the linear RNA, the circRNA was more conserved and stable. Treatment of total RNA samples with the linear digestive enzyme RNase R revealed that circKIAA1797 was more resistant to RNase R digestion than were the linear GAPDH and KIAA1797 mRNAs (Fig. 1G). Moreover, we treated cells with actinomycin D (Act D), and total RNA was collected at 0, 4, 8, and 12 h. RT‒qPCR was performed to detect circKIAA1797 and KIAA1797 mRNA expression, and the results revealed that the half-life of circKIAA1797 was longer than that of the KIAA1797 mRNA (Fig. 1H and I). Subsequently, circKIAA1797-specific convergent and divergent primers were designed, and PCR amplification was performed on three sets of samples (RNase R-untreated RNA, RNase R-treated RNA, and gDNA), which were subjected to agarose gel electrophoresis. The divergent primer amplification product (circRNA) was more resistant to RNase R digestion (Fig. 1L) than the convergent primer amplification product (linear RNA). Based on the above experimental results, we found that circKIAA1797 is indeed circular and highly expressed in lung cancer tissues and cells. Nuclear–cytoplasmic fractionation and FISH experiments were subsequently performed to explore the subcellular localisation of circKIAA1797, and circKIAA1797 was distributed mainly in the cytoplasm (Fig. 1J and K, Supplementary Fig. 1D).

### The o8G reader YBX1 increases circKIAA1797 stability

**Fig. 2:**
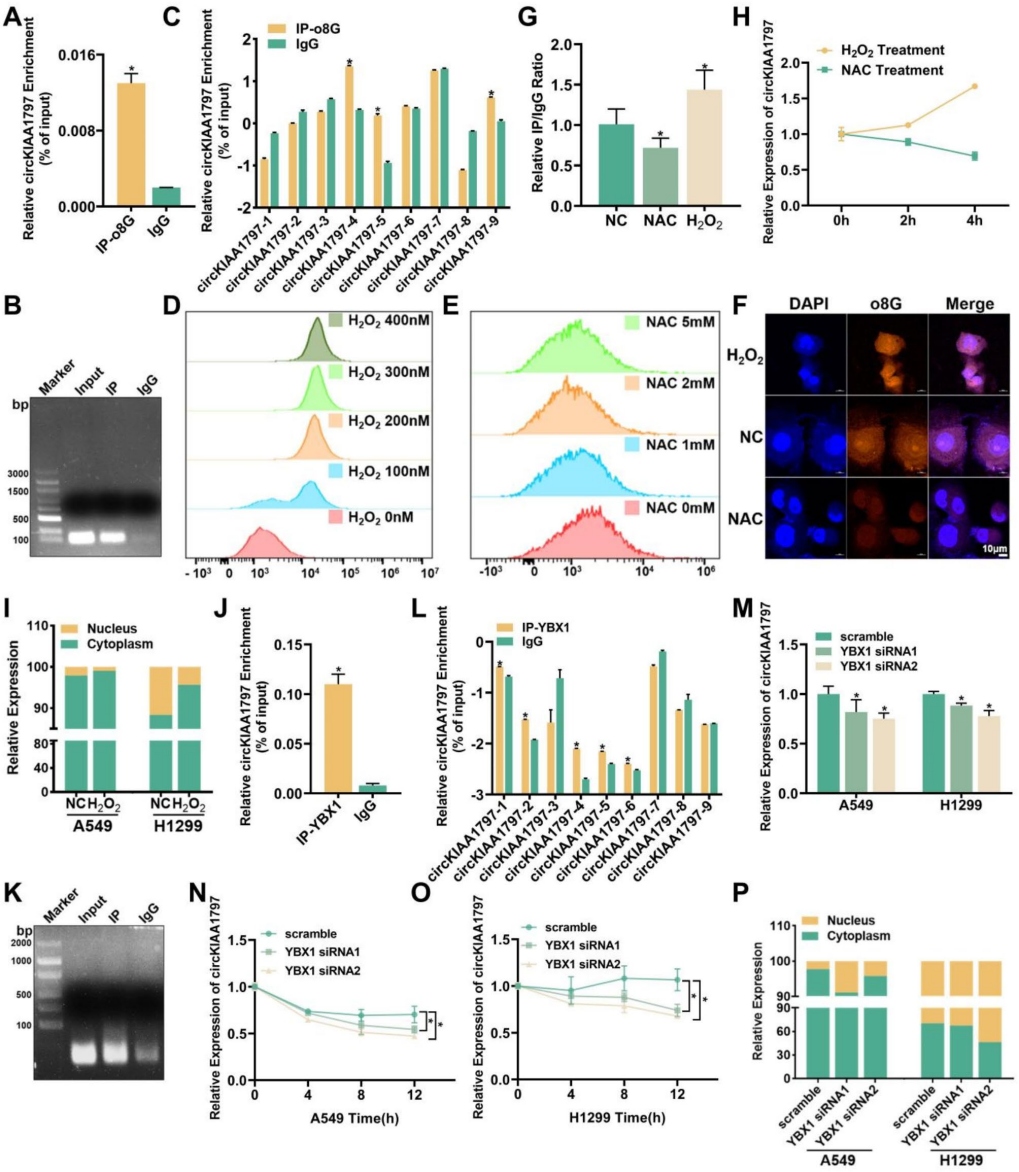
YBX1 acts as an o8G reader to increase circKIAA1797 stability. (**A** and **B**) Results of circKIAA1797 o8G RIP-qPCR and agarose gel electrophoresis. (**C**) Results of circKIAA1797 o8G-modified CLIP-qPCR. (**D**) Detection of intracellular ROS levels by flow cytometry after the treatment of cells with 0 nM, 100 nM, 200 nM, 300 nM and 400 nM H_2_O_2_. (**E**) Flow cytometry detection of intracellular ROS levels after the treatment of cells with 0 mM, 1 mM, 2 mM, or 5 mM H_2_O_2_^.^ (**F**) Three groups of samples were established, namely, the untreated NC control group, H_2_O_2_ -treated group, and NAC-treated group, and IF experiments were performed using o8G-specific antibodies. (**G**) Results of circKIAA1797 o8G RIP-qPCR in three groups of cells subjected to different treatments. (**H**) qPCR detection of circKIAA1797 expression after the treatment of cells with 2 mM NAC and 200 nM H_2_O_2_ for 0, 2 and 4 h. (**I**) Changes in the nuclear–cytoplasmic distribution of circKIAA1797 in A549 and H1299 cells were detected by a nuclear–cytoplasmic fractionation assay following H_2_O_2_ treatment. (**J** and **K**) Detection of circKIAA1797 binding to YBX1 by RIP-qPCR and agarose gel electrophoresis. (**L**) Results of CLIP-qPCR for the detection of circKIAA1797 binding to YBX1. (**M**) qPCR detection of circKIAA1797 expression after YBX1 was silenced. (**N** and **O**) After YBX1 was silenced, Act D experiments were conducted, and qPCR assays were performed to compare the half-life of circKIAA1797. (**P**) After YBX1 was silenced, a nuclear–cytoplasmic fractionation assay was conducted, and qPCR assays were performed to compare the changes in the nuclear–cytoplasmic distribution of circKIAA1797

In the above experiments, we found that circKIAA1797 was highly expressed in lung cancer tissues and cells. In addition to the traditional RNA splicing mechanism, RNA modification is also an important pathway for regulating RNA function and expression. Our research group performed a screen via IP experiments (Supplementary Fig. 2A) and revealed the presence of the o8G modification on circKIAA1797 (Fig. 2A and B). Subsequently, CLIP experiments were performed, and the o8G modification occurred mainly in segments 4, 5 and 9 of circKIAA1797 (Fig. 2C). The generation of o8G modifications is reportedly due to ROS attack; for this reason, we treated cells with H_2_O_2_ and NAC to promote and inhibit ROS accumulation in subsequent experiments. A ROS assay kit was used to detect ROS levels in the presence of different concentrations of H_2_O_2_ and NAC, and 200 nM H_2_O_2_ and 2 mM NAC were selected for subsequent experiments (Fig. 2D and E). First, IF experiments were conducted using cells treated with H_2_O_2_ and NAC, and the results confirmed that H_2_O_2_ increased the o8G modification level in cells, whereas NAC decreased the o8G modification level in cells (Fig. 2F). Second, three groups of samples (from the NAC-treated group, H_2_O_2_-treated group and control group) were subjected to o8G RIP experiments at the same time to detect the efficiency of circKIAA1797 enrichment, and compared with that in the untreated group, the efficiency of circKIAA1797 enrichment was higher in the H_2_O_2_-treated group, whereas the efficiency of circKIAA1797 enrichment was lower in the NAC-treated group (Fig. 2G). Next, the cells were treated with H_2_O_2_ or NAC for 0, 2 and 4 h, and circKIAA1797 expression was detected by RT‒qPCR. circKIAA1797 expression decreased in the NAC-treated group but increased in the H_2_O_2_-treated group in a time-dependent manner (Fig. 2H). Finally, the results of nuclear–cytoplasmic fractionation also suggested that circKIAA1797 expression in the cytoplasm increased after H_2_O_2_ treatment (Fig. 2I). These results indicate that the o8G modification promotes the expression and nuclear export of circKIAA1797 in lung cancer cells.

During RNA modification, the reader is mainly responsible for recognising and interpreting RNA molecules and translating the modification signals into functional intracellular effects [48]. To date, only two proteins, YBX1 and AUF1, have been identified as RNA o8G modification readers [26, 49]. Through bioinformatic predictions, we found that YBX1 had a stronger binding affinity for circKIAA1797 than AUF1 did (Supplementary Fig. 2B). YBX1, also known as Y-box binding protein 1, is a multifunctional DNA/RNA-binding protein containing a highly conserved cold shock domain (CSD) [50]. It plays crucial roles in various cellular processes, including transcriptional regulation [51], RNA splicing [52], DNA repair [53], and the cellular stress response [54]. Moreover, YBX1 has been reported to be aberrantly expressed in multiple cancers and closely associated with tumour progression and metastasis [50, 55, 56]. Therefore, we focused on YBX1 as an RNA o8G modification reader. Then, RIP experiments were conducted using a YBX1-specific antibody, and the results revealed that YBX1 may bind to circKIAA1797 (Fig. 2J and K). Subsequent CLIP experiments also revealed that YBX1 may bind to regions 1, 2, 4, 5, and 6 on circKIAA1797 (Fig. 2L). Next, we designed and synthesised small interfering RNAs (siRNAs) targeting YBX1, and after YBX1 was silenced (Supplementary Fig. 2C), RT‒qPCR experiments were performed. The expression of circKIAA1797 was reduced after YBX1 was silenced (Fig. 2M). Act D stability experiments revealed that the half-life of circKIAA1797 decreased after YBX1 was silenced (Fig. 2N and O). Nuclear–cytoplasmic fractionation experiments revealed that the distribution of circKIAA1797 in the cytoplasm was reduced after YBX1 was silenced (Fig. 2P). The above experimental results suggest that YBX1, as an o8G reader, can increase circKIAA1797 stability and promote its expression; moreover, YBX1 can promote circKIAA1797 nuclear export and enhance its regulatory activity.

### circKIAA1797 significantly promotes lung cancer development in vitro

**Fig. 3:**
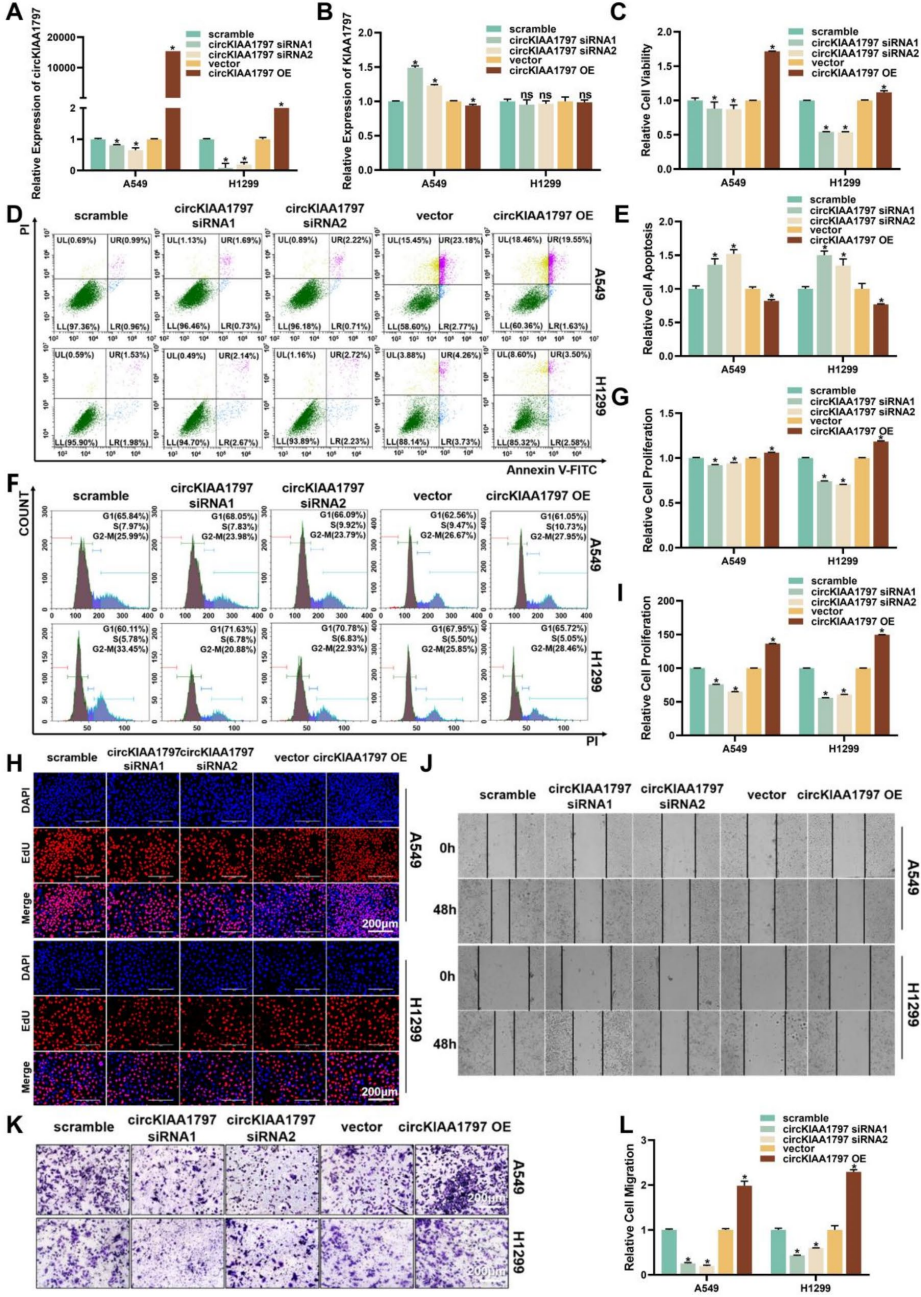
circKIAA1797 significantly promotes lung cancer progression-related behaviours in vitro. (**A**) The efficiency of transient circKIAA1797 silencing and overexpression was verified in A549 and H1299 cells. (**B**) Transient circKIAA1797 silencing and overexpression in A549 and H1299 cells were followed by qPCR to detect its parental gene efficiency. (**C**) Cell viability was determined by performing a CCK-8 assay after the transient silencing or overexpression of circKIAA1797. (**D** and **E**) Flow cytometry was used to detect apoptosis, and the statistical results are shown. (**F** and **G**) Flow cytometry detection of the cell cycle distribution and statistical results are shown. (**H** and **I**) EdU detection of cell proliferation and statistical results are shown. (**J**) Cell migration results from the wound healing assay are shown. (**K** and **L**) Transwell migration assays were performed to detect cell migration, and the statistical results are shown

Many studies have shown that differentially expressed circRNAs are the molecular basis of tumorigenesis and cancer progression. For this reason, we explored the biological function of circKIAA1797 in lung cancer cells in vitro. After the successful construction of a circKIAA1797 transient transfection system (Fig. 3A), qPCR revealed that the expression of the parent gene was not affected by circKIAA1797 silencing or overexpression in the same manner as that of the circRNA (Fig. 3B). Studies on the biological function of circKIAA1797 were subsequently performed to detect the functions of circKIAA1797 in regulating viability, apoptosis, proliferation, migration and other functions. CCK-8 experiments revealed that cell viability was decreased upon circKIAA1797 silencing and increased following circKIAA1797 overexpression (Fig. 3C). The EdU assay results suggested that cell proliferation was diminished after circKIAA1797 was silenced (Fig. 3H and I). The flow cytometry analysis of cell cycle progression and apoptosis showed that circKIAA1797 silencing suppressed cell proliferation and promoted apoptosis, whereas circKIAA1797 overexpression promoted cell proliferation and suppressed apoptosis (Fig. 3D to G). We elucidated the biological function of circKIAA1797 more comprehensively by performing wound healing and transwell migration assays to evaluate cell migration. The results showed that cell migration was reduced upon circKIAA1797 silencing and increased following circKIAA1797 overexpression (Fig. 3J to L). In summary, circKIAA1797 significantly promotes the development of lung cancer in vitro.

### circKIAA1797 significantly promotes lung cancer progression in vivo

**Fig. 4:**
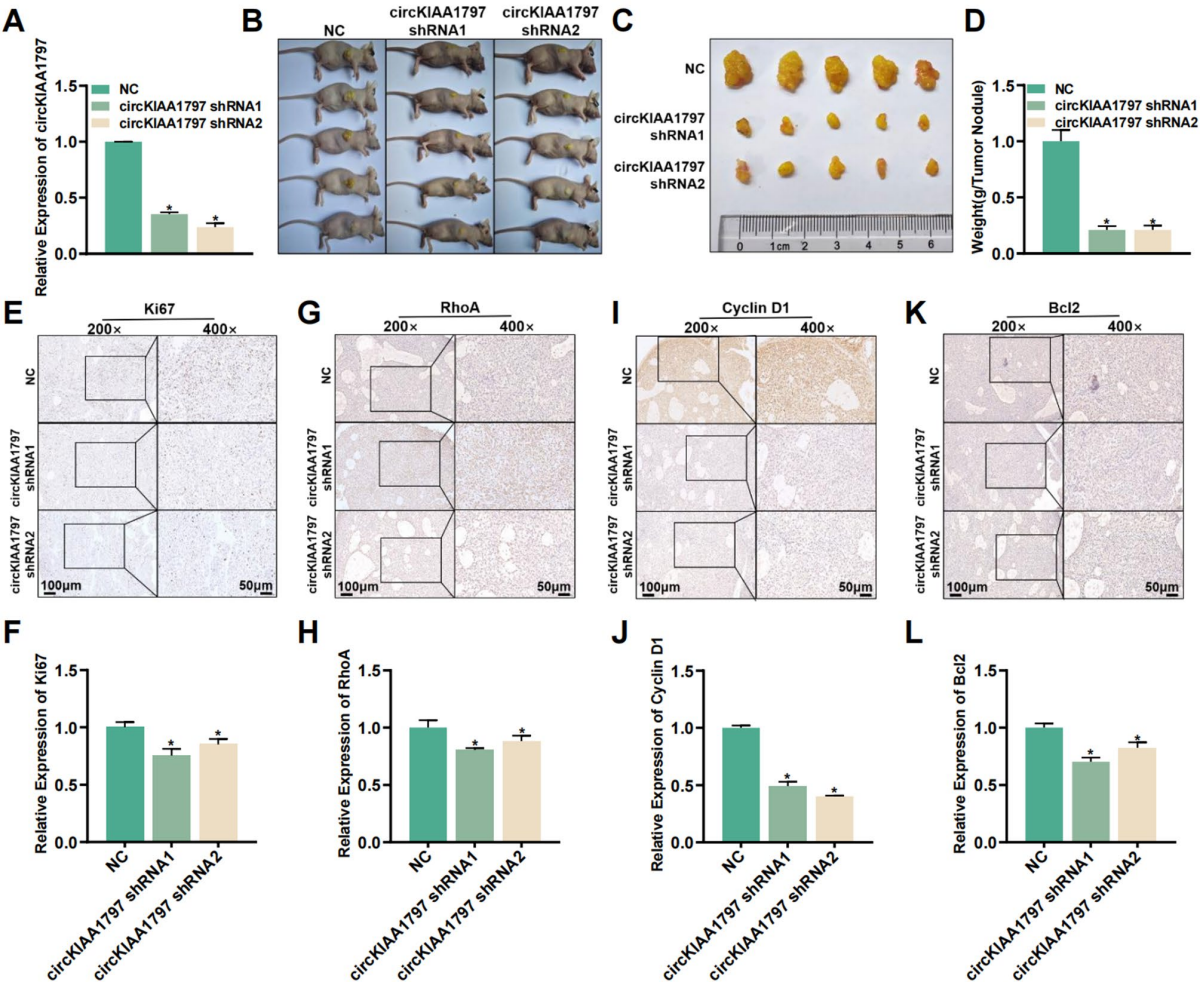
circKIAA1797 significantly promotes lung cancer progression in vivo. (**A**) qPCR was performed to detect the efficiency of stable silencing of circKIAA1797 in lentivirus-infected A549 cells. (**B**) A subcutaneous tumour formation assay in nude mice was performed using a cell line in which circKIAA1797 was stably silenced. (**C** and **D**) Tumour size in nude mice and the statistical results. (**E** to **L**) Immunohistochemical analysis of Ki67, RhoA, Cyclin D1, and Bcl2 protein expression in nude mouse tumours and quantitative analysis of the results

In the above studies, we explored the biological functions of circKIAA1797 in vitro. Next, a subcutaneous tumour formation assay in nude mice was performed to investigate the biological function of circKIAA1797 in vivo. First, we constructed A549 cells with stable circKIAA1797 silencing via lentiviral transduction (Fig. 4A). After a cell line with stable circKIAA1797 silencing was successfully constructed, these cells and control cells were subcutaneously injected into the right dorsal subcutis of nude mice. After subcutaneous tumour formation, the mice were observed for 28 days. At the end of the observation period, the nude mice were sacrificed, and the tumour tissues were subsequently removed, weighed and photographed for recording (Fig. 4B and C). The statistical analysis of the tumour weights revealed that tumour growth was slower in the group with stable circKIAA1797 silencing (Fig. 4D). We further investigated the biological function of circKIAA1797 in vivo by performing immunohistochemical experiments. In tumour tissues with stable silencing of circKIAA1797, the expression levels of the proliferation-associated protein Ki67, the cycle-associated protein Cyclin D1, the migration-associated protein RhoA and the apoptosis-associated protein Bcl2 were reduced (Fig. 4E to L). In conclusion, circKIAA1797 significantly promotes lung cancer progression in vivo.

### circKIAA1797 regulates the process of cuproptosis

**Fig. 5:**
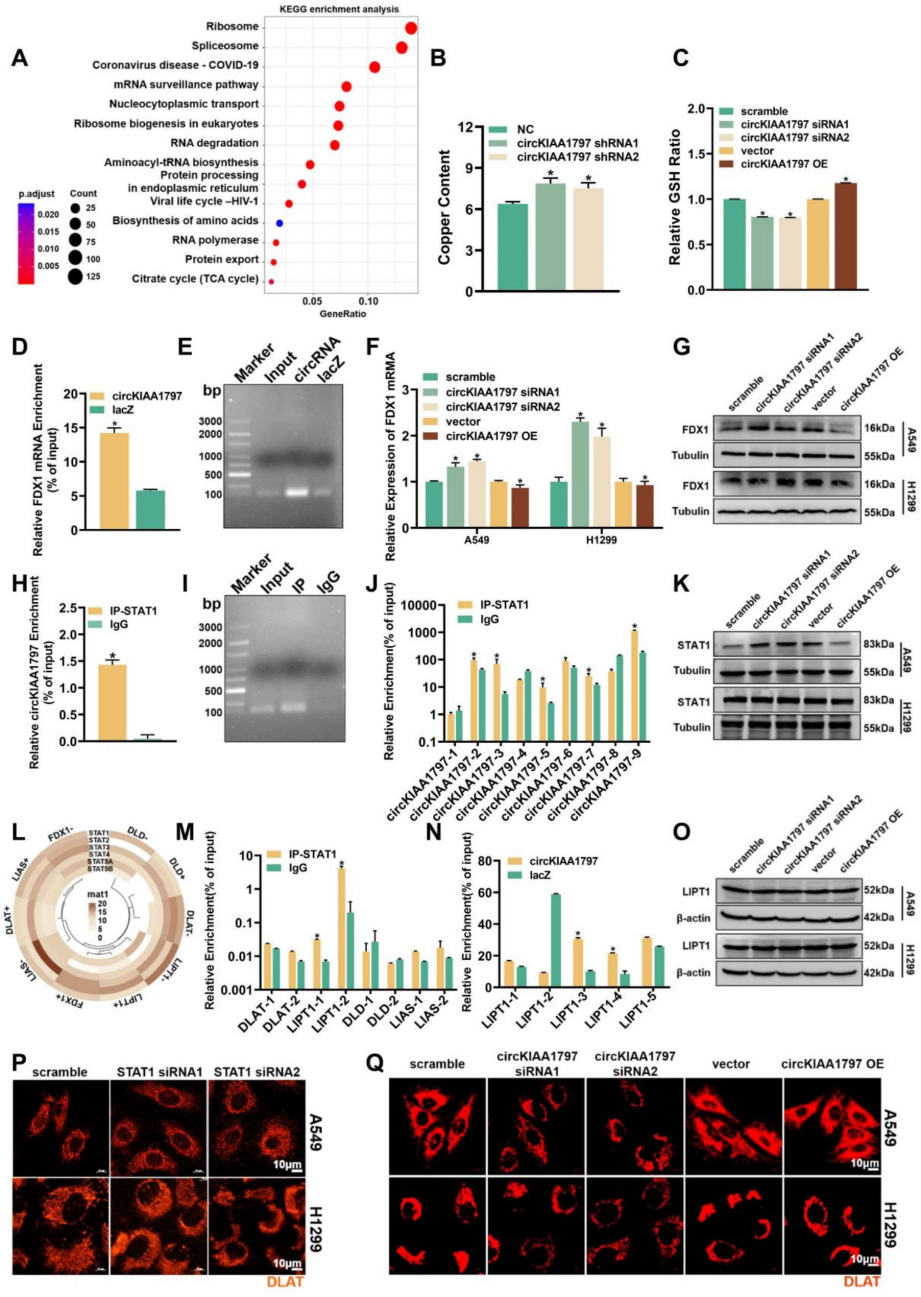
circKIAA1797 inhibits cuproptosis by suppressing FDX1 and LIPT1 expression. (**A**) KEGG pathway enrichment analysis of proteins downstream of circKIAA1797. (**B**) ICP‒MS detection of copper in cells after the stable silencing of circKIAA1797. (**C**) Reduced glutathione (GSH) levels in cells were detected after the transient silencing and overexpression of circKIAA1797. (**D** and **E**) Detection of the direct binding of circKIAA1797 to the FDX1 mRNA by ChIRP-qPCR and agarose gel electrophoresis. (**F**) Changes in FDX1 mRNA levels detected by qPCR after the transient silencing and overexpression of circKIAA1797. (**G**) Changes in FDX1 protein levels detected by Western blot after the transient silencing and overexpression of circKIAA1797. (**H** and **I**) Detection of circKIAA1797 binding to STAT1 by RIP-qPCR and agarose gel electrophoresis. (**J**) CLIP-qPCR results showing that circKIAA1797 binds to STAT1. (**K**) Western blot analysis of STAT1 protein expression after the transient silencing or overexpression of circKIAA1797. (**L**) The JASPAR website was used to predict the ability of the STAT family to bind to the FDX1, DLD, DLAT, LIPT1, and LIAS promoter regions. (**M**) ChIP‒qPCR results showed STAT1 binding to the DLAT, LIPT1, DLD, and LIAS promoter regions. (**N**) ChIRP–qPCR results showed the circKIAA1797-mediated regulation of LIPT1 transcription through the STAT1 protein. (**O**) Western blot analysis of LIPT1 protein expression after the transient silencing or overexpression of circKIAA1797. (**P**) DLAT oligomerization was detected by IF staining after the transient silencing of STAT1 in A549 and H1299 cells. (**Q**) DLAT oligomerization was detected by IF staining after the transient silencing and overexpression of circKIAA1797 in A549 and H1299 cells

Through the above functional experiments, we found that circKIAA1797 significantly promoted the development of lung cancer both in vivo and in vitro. We conducted TRAP-MS experiments (Supplementary Fig. 3A) and bioinformatic analyses of mass spectrometry results to further elucidate the molecular mechanism by which circKIAA1797 promotes the development of lung cancer (Data S2). The KEGG pathway analysis revealed that circKIAA1797 was closely associated with the tricarboxylic acid (TCA) cycle (Fig. 5A). These results suggest that cuproptosis occurs through the direct binding of copper to lipoylated components of the TCA cycle. Therefore, we predicted the binding of circKIAA1797 to 10 cuproptosis-associated proteins (FDX1, DLAT, DLST, LIAS, ATP7A, ATP7B, SLC31A1, GCSH, LIPT1, and DLD) via the *cat*RAPID website (http://service.tartaglialab.com/) [57]. These results suggested that circKIAA1797 may bind to multiple cuproptosis-associated proteins (Supplementary Fig. 3B). We performed ICP‒MS experiments to further elucidate the relationship between circKIAA1797 and cuproptosis. The results suggested that copper ion levels were higher in cells with stable circKIAA1797 silencing than in control cells (Fig. 5B). The results of the GSH assay suggested that GSH levels were decreased in circKIAA1797-silenced cells, whereas GSH levels were increased following the overexpression of circKIAA1797 (Fig. 5C). Based on the above experiments, we conclude that circKIAA1797 regulates the level of cuproptosis in lung cancer cells.

The vast majority of circRNAs are not directly involved in protein synthesis but exert their regulatory effects at the cellular function level. circRNAs can participate in downstream regulation in a variety of ways, including by acting as miRNA sponges, by directly binding RNAs, and by acting as protein sponges. We therefore suggest that the regulation of circKIAA1797 in cuproptosis is also diverse. On the one hand, we identified the possibility of the direct binding of circKIAA1797 to the FDX1 mRNA, a key regulator of cuproptosis, based on IntaRNA website (http://rna.informatik.uni-freiburg.de/) predictions (Supplementary Fig. 3C) [58]. A circKIAA1797-specific ChIRP probe was subsequently designed, and ChIRP experiments were conducted to reveal the direct binding of circKIAA1797 to the FDX1 mRNA (Fig. 5D and E). Finally, RT‒qPCR and Western blot experiments were performed to detect FDX1 mRNA and protein levels, and both the FDX1 mRNA and protein levels were increased upon the silencing of circKIAA1797, whereas the FDX1 mRNA and protein levels were decreased following the overexpression of circKIAA1797 (Fig. 5F and G, Supplementary Fig. 3D). On the other hand, when we reviewed the related literature, we found that key proteins such as LIAS, LIPT1, and DLD are lipidation-related enzymes and that the STAT family is the key family that controls lipid metabolism [59]. Considering these results and the TRAP-MS results, we selected STAT1, which had the highest binding score, as the research object. RIP and CLIP experiments were conducted, and the results suggested that STAT1 binds to segments 2, 3, 5, 7, and 9 of circKIAA1797 (Fig. 5H to J). Western blot analysis revealed that STAT1 expression increased when circKIAA1797 was silenced, whereas circKIAA1797 overexpression decreased STAT1 expression (Fig. 5K and Supplementary Fig. 3E). The binding site of STAT1 to the promoter regions of several cuproptosis-related proteins (FDX1, LIAS, DLAT, LIPT1 and DLD) was subsequently predicted using the JASPAR website (https://jaspar.elixir.no/) (Fig. 5L) [60]. Specific primers were designed for the binding sequences, and STAT1 could bind to the LIPT1 promoter region. ChIRP and ChIP experiments were performed, and circKIAA1797 targeted the transcription factor STAT1 and affected the transcription of the fatty acyltransferase LIPT1 (Fig. 5M and N). RT‒qPCR and Western blot experiments were performed to detect the RNA and protein levels, and silencing circKIAA1797 increased LIPT1 mRNA and protein levels, whereas the overexpression of circKIAA1797 decreased LIPT1 mRNA and protein levels (Fig. 5O, Supplementary Fig. 3F and 3 K). Finally, IF experiments suggested that the extent of DLAT oligomerization increased after FDX1 (Supplementary Fig. 3G) or LIPT1 was overexpressed. The level of DLAT oligomerization increased after the silencing of circKIAA1797 and decreased after the overexpression of circKIAA1797. DLAT oligomerization decreased after STAT1 was silenced (Fig. 5P and Q). In conclusion, the above experiments reveal that circKIAA1797 affects cuproptosis by inhibiting the expression of the cuproptosis-associated proteins FDX1 and LIPT1 through mechanisms such as decreasing the stability of FDX1 by binding to the FDX1 mRNA and inhibiting the transcription of LIPT1 by binding to the transcription factor STAT1.

### circKIAA1797 promotes DLAT lipoylation through the inhibition of FDX1 expression

**Fig. 6:**
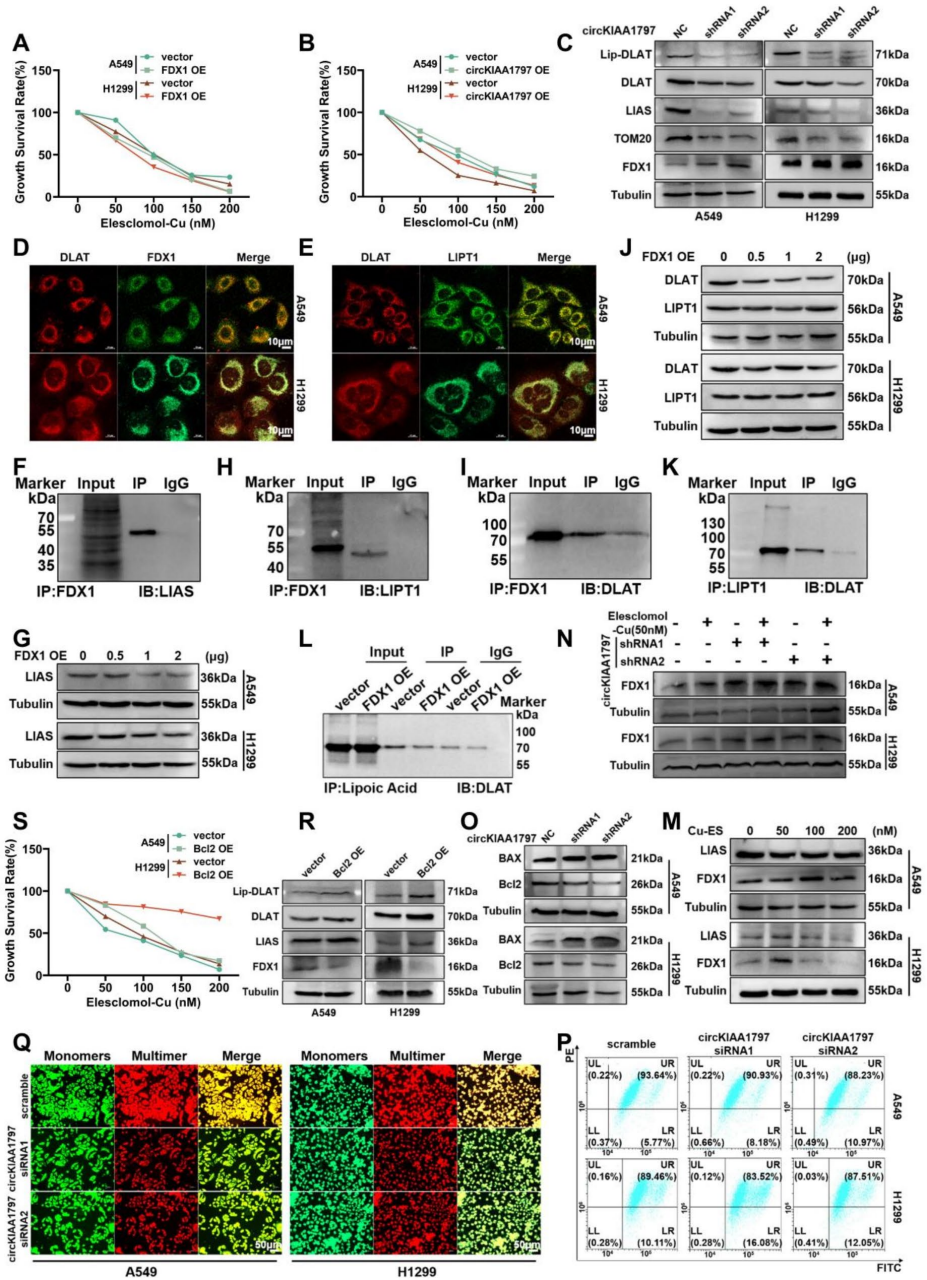
Exploration of the mechanism of cuproptosis induction. (**A**) After the transient overexpression of FDX1, a CCK-8 assay was used to detect changes in cellular resistance to cuproptosis. (**B**) After the transient overexpression of circKIAA1797, a CCK-8 assay was performed to detect changes in cellular resistance to cuproptosis. (**C**) Western blot analysis of Lip-DLAT, DLAT, LIAS, TOM20, and FDX1 protein expression after the stable silencing of circKIAA1797. (**D**) Immunofluorescence staining showed the colocalisation of the DLAT and FDX1 proteins. (**E**) Immunofluorescence staining was performed to detect the colocalisation of DLAT with the LIPT1 protein. (**F**) Western blot results of the Co-IP assay used to detect FDX1 binding to LIAS. (**G**) Western blot analysis of LIAS protein expression levels after the gradient overexpression of FDX1. (**H**) Western blot results of the Co-IP assay for detecting FDX1 binding to LIPT1. (**I**) Western blot results of Co-IP experiments for detecting FDX1 binding to DLAT. (**J**) Gradient overexpression of FDX1 followed by Western blot detection of DLAT and LIPT1 protein expression levels. (**K**) Western blot results of the Co-IP assay used to detect LIPT1 binding to DLAT. (**L**) After the overexpression of FDX1, Co-IP experiments were performed to detect changes in the ability of lipoic acid to bind DLAT. (**M**) Cells were treated with 0 nM, 50 nM, 100 nM and 200 nM elesclomol-Cu, and Western blot was performed to examine the protein expression of LIAS and FDX1. (**N**) circKIAA1797 stably transfected cells were treated with 50 nM elesclomol-Cu, and FDX1 protein expression was examined by Western blot. (**O**) Western blot analysis of BAX and Bcl2 protein expression in cell lines with stable circKIAA1797 silencing. (**P**) Flow cytometry detection of changes in the mitochondrial membrane potential after the transient silencing of circKIAA1797. (**Q**) Fluorescence microscopy comparing the changes in JC monomer and multimer levels after the transient silencing of circKIAA1797. (**R**) After the overexpression of Bcl2, Western blot assays were performed to detect Lip-DLAT, DLAT, LIAS, and FDX1 protein expression. (**S**) After the overexpression of Bcl2, a CCK-8 assay was performed to detect changes in cellular resistance to cuproptosis

In the above experiments, we elucidated the regulatory relationships between circRNAs and several cuproptosis-related proteins. Next, we performed CCK-8 experiments after overexpressing circKIAA1797 or FDX1. Compared with that in the control group, cell viability in the circKIAA1797-overexpressing group was higher, and cell viability in the FDX1-overexpressing group was lower after treatment with different concentrations of elesclomol-Cu (Fig. 6A and B). Cuproptosis-associated protein expression was subsequently examined in stably silenced circKIAA1797 cells. Western blot experiments revealed that the silencing of circKIAA1797 resulted in increased expression of iron‒sulfur protein (FDX1), whereas the expression of pyruvate dehydrogenase complex isoform (DLAT), lipoylated pyruvate dehydrogenase complex isoform (Lip-DLAT), lipoic acid synthase (LIAS), and mitochondrial outer membrane protein (TOM20) decreased (Fig. 6C, Supplementary Fig. 4F to 4 J). We further clarified the role of circKIAA1797 in the regulation of cuproptosis by performing immunohistochemical experiments. The results revealed that the expression of FDX1 and LIPT1 increased in tissues after the stable silencing of circKIAA1797 (Supplementary Fig. 4K to 4 N). The above tissue assay results were consistent with the results of cell-based experiments.

Currently, the mainstream view is that LIPT2 transfers lipid groups to glycine-cleaved H protein (GCSH), which is catalysed by LIAS, and then transfers them to DLAT via LIPT1 to activate the TCA cycle (Supplementary Fig. 4A); cuproptosis occurs in this process, but the regulatory mechanism is still unclear. We downloaded cuproptosis-related protein expression data from the Xena database and visualised the expression profiles after an analysis using SPSS software. The results suggested that FDX1 expression was correlated with DLAT, LIPT1 and LIAS expression (Supplementary Fig. 4B). The results of IF experiments revealed obvious colocalisation between DLAT and FDX1 and between DLAT and LIPT1 (Fig. 6D and E). Based on the above evidence, we further performed Co-IP experiments and found that FDX1 could bind to LIAS, DLAT and LIPT1 (Fig. 6F, H and I). Western blot experiments revealed that when cells were transfected with 0, 0.5, 1, 2 µg of the FDX1 overexpression plasmid, the expression of LIAS decreased with increasing FDX1 levels (Fig. 6G and Supplementary Fig. 4C), whereas the expression of DLAT and LIPT1 did not change (Fig. 6J, Supplementary Fig. 4D and 4E). According to the related literature and Co-IP experiments, LIPT1 bound to DLAT (Fig. 6K). The protein structures were subsequently retrieved from UniProt, and the full-length AlphaFold-predicted structures of ODP2 HUMAN (UniProt ID: P10515), ADX HUMAN (UniProt ID: P10109), and LIPT_HUMAN (UniProt ID: Q9Y234) were subsequently selected as protein structure files using HADDOCK to predict the structure of the ternary complex. The results suggested the occurrence of two-by-two interactions between the three compounds to form a stable ternary complex (Supplementary Fig. 4O). We performed Co-IP experiments to further explore the relationships among FDX1, DLAT and LIPT1, and the results suggested that the overexpression of FDX1 reduced the ability of lipoic acid to bind to DLAT (Fig. 6L). Based on the results of the above experiments, FDX1, LIPT1, and DLAT likely form a ternary complex, and FDX1 inhibits the lipoylation of DLAT via LIPT1. Overall, we believe that circKIAA1797 can activate the TCA cycle by inhibiting the expression of FDX1, promoting lipid-based transfer, and catalysing DLAT-mediated reactions (Supplementary Fig. 4Q).

### circKIAA1797 inhibits cuproptosis by promoting mitochondrial permeability transition pore (mPTP) closure

The optimal concentration of elesclomol-Cu for treatment was determined via Western blot, and the results showed that FDX1 protein expression peaked after treatment with a concentration of 50 nM (Fig. 6M). Therefore, 50 nM elesclomol-Cu was selected for subsequent experiments. Elesclomol-Cu treatment was administered the cell line with stably silenced circKIAA1797, and the degree of FDX1 upregulation was greater in the circKIAA1797-silenced group than in the control group (Fig. 6N and Supplementary Fig. 5E). Based on the results of this study, we hypothesised that silencing circKIAA1797 would improve the effect of elesclomol-Cu treatment. According to the related literature, cuproptosis may involve a disruption of the mitochondrial membrane and the release of cytochrome C, and the main channel involved is the mPTP [61]; therefore, we chose the mPTP as the research object. The results of the RIP experiments suggested that the mPTP constituent protein ANT2 could bind to circKIAA1797 (Supplementary Fig. 5A). Western blot analysis revealed that after stable silencing of circKIAA1797, the expression of the mPTP constitutive protein ANT2 (Supplementary Fig. 5B and 5 C) and the mPTP closing protein Bcl2 decreased, whereas the expression of the mPTP opening protein BAX increased (Fig. 6O, Supplementary Fig. 5F and 5G). JC-1 staining experiments were performed to further explore the regulation of the mPTP by circKIAA1797, circKIAA1797 silencing resulted in diminished JC-1 aggregation (indicated by red fluorescence) and depolarisation of the mitochondrial membrane potential (Δψ) (Fig. 6P and Q). Based on the above experiments, circKIAA1797 regulates the mPTP status (open vs. closed).

The opening of the mPTP leads to a significant increase in the permeability of the inner and outer mitochondrial membranes, allowing small molecules to freely traverse them, but the relationship between the mPTP and cuproptosis is not yet clear. For this reason, we overexpressed the mPTP-closing protein Bcl2 and performed Western blot experiments. The results revealed that the overexpression of Bcl2 decreased FDX1 protein expression and increased DLAT, Lip-DLAT, and LIAS protein expression, suggesting that the level of cuproptosis decreased (Fig. 6R, Supplementary Fig. 5H to 5 K). CCK-8 experiments revealed that the overexpression of Bcl2 resulted in increased cell viability, and the cells were more resistant to elesclomol-Cu treatment (Fig. 6S). The results of IF experiments also showed that the level of DLAT oligomerization decreased after Bcl2 treatment. Together, these findings indicate that circKIAA1797 can reduce the extent of cuproptosis by promoting mPTP closure.

### circKIAA1797 promotes lung cancer development by inhibiting cuproptosis

**Fig. 7:**
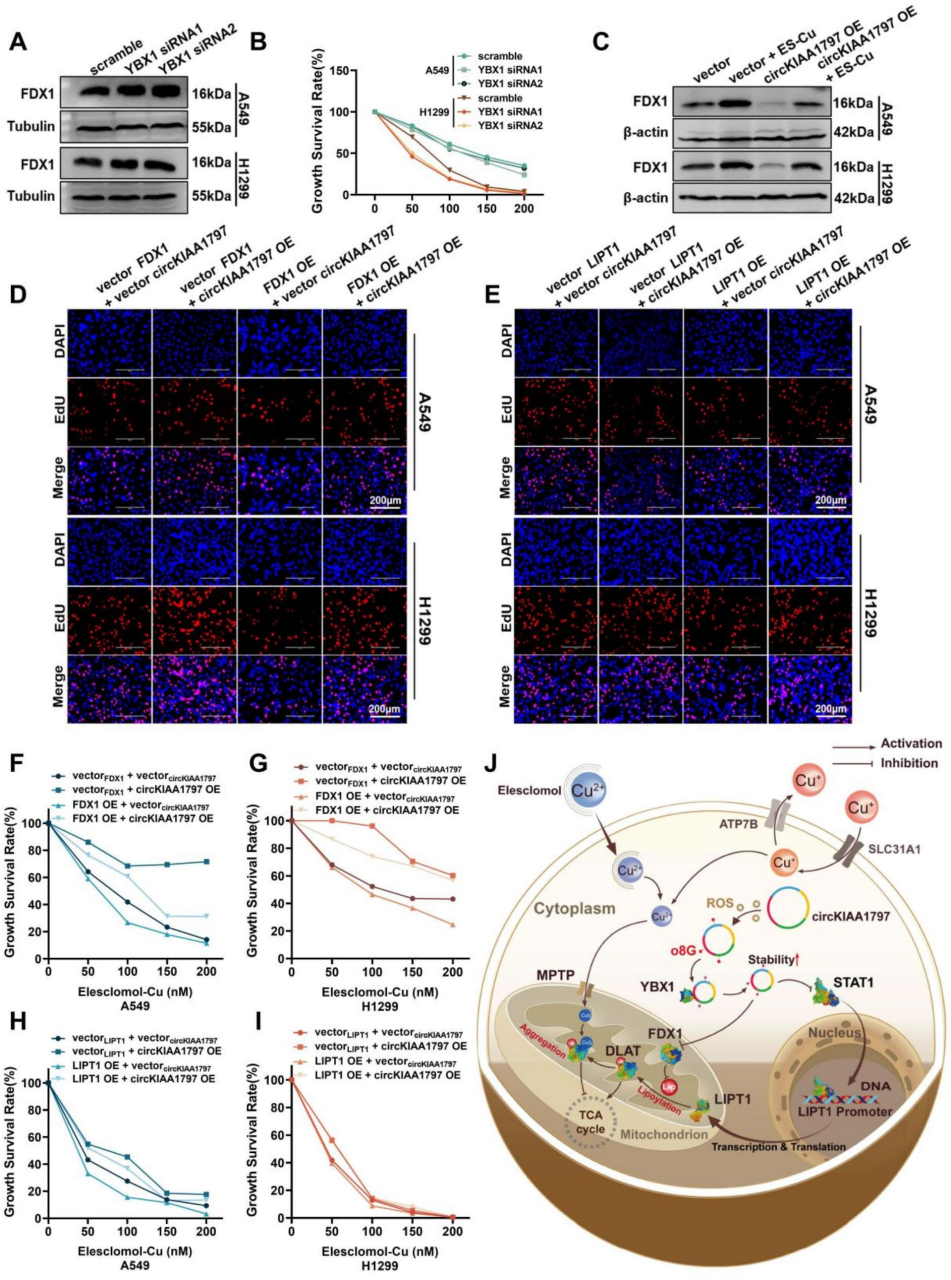
.circKIAA1797 promotes lung cancer development by inhibiting cuproptosis. (**A**) After the transient silencing of YBX1, Western blot analysis was performed to detect FDX1 protein expression. (**B**) After YBX1 was silenced, a CCK-8 assay was performed to detect changes in cellular resistance to cuproptosis. (**C**) After the transient overexpression of circKIAA1797, Western blot analysis was performed to detect FDX1 protein expression. (**D**) After the simultaneous overexpression of circKIAA1797 and FDX1, an EdU assay was performed to detect changes in cell proliferation. (**E**) After the simultaneous overexpression of circKIAA1797 and LIPT1, an EdU assay was performed to detect changes in cell proliferation. (**F** and **G**) After the simultaneous overexpression of circKIAA1797 and FDX1, a CCK-8 assay was performed to detect changes in cellular resistance to cuproptosis. (**H** and **I**) After the simultaneous overexpression of circKIAA1797 and LIPT1, a CCK-8 assay was conducted to detect changes in cellular resistance to cuproptosis. (**J**) The presence of o8G on circKIAA1797 and the o8G reader YBX1 increased circKIAA1797 stability and nuclear export. circKIAA1797 can inhibit FDX1 protein expression by decreasing the stability of the FDX1 mRNA and directly binding the transcription factor STAT1, inhibiting LIPT1 transcription, whereas circKIAA1797 promotes mPTP closure, ultimately inhibiting cuproptosis to promote lung cancer development

The above experiments revealed that the o8G reader YBX1 promotes circKIAA1797 expression and that circKIAA1797 is able to reduce the extent of cuproptosis by modulating key regulatory signalling pathways and the mPTP; however, whether YBX1 can affect cuproptosis is not yet known. Western blot experiments were performed and revealed that silencing YBX1 increased FDX1 protein expression (Fig. 7A and Supplementary Fig. 6A). The results of the CCK-8 assay revealed that, compared with those in the control group, the ability of cells to resist cuproptosis was weakened in the YBX1-silenced group (Fig. 7B). Based on the above experiments, we conclude that the circKIAA1797-bound o8G reader YBX1 can regulate cuproptosis. Subsequently, Western blot experiments were performed, and the results suggested that elesclomol-Cu reversed the decrease in the expression level of FDX1 caused by the overexpression of circKIAA1797 (Fig. 7C and Supplementary Fig. 5D).

We further explored whether circKIAA1797 affects the level of cellular cuproptosis by regulating FDX1, LIPT1 and the mPTP by designing circKIAA1797 rescue experiments in which FDX1, LIPT1 and the mPTP were targeted. We conducted EdU assays to detect cell viability. The overexpression of FDX1 or LIPT1 inhibited cell proliferation, whereas the overexpression of circKIAA1797 increased cell proliferation. The simultaneous overexpression of FDX1 or LIPT1 with circKIAA1797 reversed the increase in cell proliferation (Fig. 7D and E, Supplementary Fig. 6D and 6E). Cell viability was also assayed using a CCK-8 assay. The overexpression of either FDX1 or LIPT1 decreased cellular resistance to cuproptosis, whereas the overexpression of circKIAA1797 increased cellular resistance to cuproptosis. Simultaneous overexpression of FDX1 or LIPT1 with circKIAA1797 reversed cellular resistance to cuproptosis (Fig. 7F to I). The overexpression of Bcl2 inhibited mPTP opening and increased cellular resistance to cuproptosis, whereas the silencing of circKIAA1797 decreased cellular resistance to cuproptosis. Simultaneous overexpression of Bcl2 and silencing of circKIAA1797 reversed the decrease in cellular resistance to cuproptosis (Supplementary Fig. 6B and 6 C). In summary, o8G-modified circKIAA1797 inhibits cellular cuproptosis through FDX1, LIPT1 and the mPTP and promotes lung cancer development (Fig. 7J).

## Discussion

Lung cancer is an important cause of cancer-related death, but effective measures for early diagnosis and treatment are still lacking [62]. Therefore, an urgent need exists to elucidate the molecular mechanisms involved in lung cancer development and explore new therapeutic targets to improve the diagnosis and prognosis of lung cancer patients. With the vigorous development of high-throughput sequencing technology, a special class of RNAs (circRNAs, which have a closed loop structure) has attracted widespread attention. Compared with other RNAs, circRNAs are more conserved, stable and specific [12]. Due to these characteristics, circRNAs are considered to have potential as tumour biomarkers and therapeutic targets for the early screening and subsequent treatment of tumours [63]. In this study, we found that circKIAA1797 is highly expressed in lung cancer cell lines and tissues through a combination of high-throughput sequencing and qPCR experiments. Xiaomei Zheng et al. reported that exosomal circKIAA1797 can inhibit the development of gastric cancer by regulating the miR-4429/PBX3 axis [64]. Unfortunately, Xiaomei Zheng et al. reported that the circRNA ID of circKIAA1797 is hsa_circ_0003537. A search of the circBank website (http://www.circbank.cn/) revealed that hsa_circ_0003537 is composed of exons 24, 25 and 26 of chromosome 9, whereas the circRNA ID of circKIAA1797 in our study was hsa_circ_0003295, which is composed of exons 14, 15, 16 and 17 of chromosome 9. Therefore, the two circRNA sequences are completely different. The function of circKIAA1797 (hsa_circ_0003295) in human lung cancer is not yet clear. Therefore, we performed in vivo and in vitro studies. The experimental results showed that circKIAA1797 (hsa_circ_0003295) can promote the development of lung cancer by modulating various functions, including cell viability, proliferation, and invasion. We are the first to identify that the o8G modification occurs on circKIAA1797, to clarify the biological function of o8G-modified circRNAs in lung cancer development, and to elucidate the underlying molecular mechanism involved in promoting lung cancer development. Our study provides not only a new perspective for the study of the mechanisms regulating circRNA expression but also new ideas for the diagnosis and treatment of lung cancer from an epigenetic perspective.

The o8G modification is an important type of oxidative modification of RNA. This modification was initially discovered on DNA, and since guanine has the lowest redox potential on DNA, 8-oxo-dG is the most common oxidised form produced by reacting with oxygen at the C8 position [65]. 8-oxo-dG is highly mutagenic, leading to the mutation of guanine to thymine during DNA replication. Compared with DNA molecules, RNA molecules are more susceptible to oxidative modifications, which may be related to the molecular characteristics of RNA such as a 2’-hydroxyl structure (which is more susceptible to reactions), single-stranded DNA (which lacks protein protection), and a lack of damage repair mechanisms [26]. o8G is the most abundant product of RNA oxidation and is susceptible to further oxidation and strand breaks. Due to the rapid turnover of RNA molecules, few reports related to the o8G modification of RNA have been published; however, not all RNAs are unstable, and many have long half-lives; for example, rRNAs and tRNAs have half-lives of several days. In general, circRNAs have a longer half-life than do rRNAs and tRNAs because of their special structure, which provides a theoretical basis for studying o8G modifications on circRNAs. During tumorigenesis, the rapid increase in intracellular ROS levels due to mitochondrial dysfunction [66], changes in the tumour microenvironment (hypoxia and inflammation) [67, 68], and rapid proliferation [69] and metabolic demands [70, 71] of cells provide a factual basis for studying RNA o8G modifications in tumours. In this study, H_2_O_2_ was used to regulate ROS levels and induce o8G modification of RNA; we expected the o8G modification to increase the stability of circKIAA1797, but the experimental results did not confirm our expectations. An increase in ROS levels, in addition to oxidative modifications, can also activate RNA degradation mechanisms and affect binding protein expression and activity [72–74]. Therefore, further explorations of the corresponding o8G modification “writers” and “erasers” are needed to achieve precise regulation. YBX1 and AUF1 have been reported to act as RNA o8G readers [49]. Since no TRAP-MS results were obtained for AUF1, we chose YBX1 as the focus of our study. The YBX1 gene encodes a highly conserved cold-shock structural domain protein with a wide range of nucleic acid-binding regions that participates in many cellular processes, including DNA repair [55], transcriptional regulation [75], and mRNA processing and stabilisation [76, 77]. YBX1 contains a nuclear localisation signal (NLS) and a nuclear export signal (NES) [78, 79], which are signal sequences that enable YBX1 to be dynamically regulated and transported between the nucleus and cytoplasm according to cellular needs. The survival analysis and immune cell infiltration analysis revealed that YBX1 promotes the progression of lung cancer (Supplementary Fig. 2D to 2 F). In addition, YBX1 can increase the stability of m5C-modified mRNAs by binding to these mRNAs, which in turn affects the protein expression level [80–82]. Based on these findings, we explored the stability and nuclear–cytoplasmic distribution of o8G-modified circKIAA1797, and the results showed that the increase in circKIAA1797 expression and distribution in the cytoplasm after the increase in the level of the o8G modification was caused by the binding of the o8G reader YBX1. The m6A reader YTHDC1 accelerates the translocation of m6A-modified circHPS5 from the nucleus to the cytoplasm and regulates the expression of HMGA2 through the sponge miR-370. These findings suggest that the increased cytoplasmic distribution of circKIAA1797 induced by the o8G reader YBX1 also enhances the regulatory role of circKIAA1797 in the cytoplasm. Studies of the o8G modification are still in the preliminary exploration stage. The o8G modification has been shown to alter miRNA targeting and affect miRNA stability and maturation [27, 28]; however, the effect of the o8G modification on circRNAs is still unclear. In addition to the effects of the o8G modification on circKIAA1797 stability and the nuclear–cytoplasmic distribution, as discussed above, we explored the impact of o8G on the binding ability of circRNAs. We performed ChIP, ChIRP and RIP experiments to test the ability of circKIAA1797 to directly bind to DNA, RNA and protein and found that after cells were treated with H_2_O_2_ and the level of the o8G modification increased, the ability of circKIAA1797 to bind to the FDX1 mRNA and the LIPT1 promoter region were greatly decreased, whereas the ability of circKIAA1797 to bind to the STAT1 protein increased (Supplementary Fig. 3H to 3 J). We believe that the loss of binding to RNA and DNA caused by the increased level of the o8G modification on circRNA is due to the o8G modification affecting base mutations, which induces circRNA to lose its original target recognition ability, thus affecting the gene expression pattern. The reason for the increase in the binding of circKIAA1797 to STAT1 caused by the o8G modification still needs to be further explored. In summary, this study revealed the existence of o8G modifications on circRNAs and revealed that YBX1 can act as an o8G modification reader to increase the stability and nuclear export of circKIAA1797, which provides a new perspective for studying the regulation of circRNA expression.

circRNAs are a special class of RNA molecules that mainly perform regulatory functions [83]. Therefore, in the exploration of downstream mechanisms, we used various approaches to determine whether circKIAA1797 can regulate cuproptosis. We found that circKIAA1797 exerts its molecular effects at three levels: RNA‒RNA interaction, RNA‒protein interaction, and RNA transcriptional regulation. At the cellular level, circKIAA1797 can directly regulate the cuproptosis signalling pathway and, by affecting the opening and closing of the mitochondrial mPTP, regulate the mitochondrial copper content, ultimately regulating cuproptosis. Currently, the underlying mechanisms of cuproptosis are very complicated and include the oxidative stress pathway, which is induced by excessive ROS-mediated damage to the cell structure; the mitochondrial pathway, which is caused by disruption of the mitochondrial membrane and release of cytochrome C; the endoplasmic reticulum pathway, which induces endoplasmic reticulum stress to activate the unfolded protein response (UPR); and the autophagy pathway, which triggers cellular self-digestion. With respect to the lipoylation process, previous studies suggested that LIPT2 transfers fatty acyl groups to the glycine-cleaved H protein GCSH, which is catalysed by LIAS, and that LIPT1 transfers fatty acyl groups to DLAT, thereby activating the TCA cycle [84]. In recent years, Todd R. Golub et al. reported that the FDX1 protein is necessary for cuproptosis to occur [34]. Peter Tsvetkov et al. reported that FDX1 binds directly to LIAS and promotes its role in cellular proteolipidation [85]. Based on these findings, we propose a new hypothesis that FDX1 can directly regulate LIPT1 and DLAT expression and participate in the process of lipoylation (Supplementary Fig. 4A). Therefore, based on the basis correlations of FDX1 expression with LIPT1 and DLAT expression identified by the bioinformatics analysis (Supplementary Fig. 4B), we conducted Co-IP experiments, which confirmed the existence of a direct binding relationship between FDX1, LIPT1 and DLAT. According to the Western blot-based validation experiments, no dose‒response relationship exists between LIPT1, DLAT and FDX1. We further explored the regulatory effect of FDX1 on these two genes by performing Co-IP experiments, which revealed that the overexpression of FDX1 resulted in a decrease in the enrichment of the lipid group Lip on DLAT. These results suggest that FDX1, LIPT1, and DLAT form a ternary complex that coregulates cuproptosis. This finding also explains the decrease in Lip-DLAT levels observed after an increase in FDX1 expression in cells undergoing compensatory cuproptosis. Considering that Todd R. Golub et al. reported that cuproptosis is caused by the oligomerization of Lip-DLAT upon binding to copper, we suggest that this decrease in Lip-DLAT levels caused by FDX1 (Supplementary Fig. 4Q) is another major cause of TCA cycle disorders [34]. In the regulation of the cuproptosis pathway by circKIAA1797, in addition to the key signalling pathways involved in cuproptosis mentioned above, we also conducted in-depth research on the influence of the mPTP on cuproptosis. Previous studies have shown that copper may trigger cell apoptosis by disrupting the mitochondrial membrane and releasing cytochrome C, while the opening of mPTPs can lead to the loss of the mitochondrial membrane potential and an imbalance of substance exchange inside and outside the mitochondria, which triggers a series of downstream responses, including cytochrome C release. Based on these findings, we propose the following hypothesis: the opening and closing of the mPTP may be regulated to maintain copper homeostasis in mitochondria. Bcl2 and BAX are key proteins involved in the regulation of apoptosis, and they play important roles in regulating the mPTP. Bcl2 is an antiapoptotic protein, and its main function is to inhibit apoptosis [86, 87]. Bcl2 is also capable of stabilising the mitochondrial membrane by interacting with other proteins on the outer mitochondrial membrane, preventing the mPTP from opening and closing, and inhibiting cytochrome C release. Therefore, we inhibited the opening of the mPTP by overexpressing Bcl2. Western blot analysis revealed that overexpression of Bcl2 decreased the expression of FDX1 and increased the expression of the LIAS, DLAT, and Lip-DLAT proteins, which indicated that the level of intracellular cuproptosis decreased. The results of the CCK-8 and IF experiments also showed that the overexpression of Bcl2 decreased the level of DLAT oligomerization and that the cells were more resistant to cuproptosis. These experimental results indicate that copper can enter and exit mitochondria through the mPTP and regulate cuproptosis. We also performed a preliminary exploration of the connection between RNA modification and cuproptosis. After the o8G reader YBX1 was silenced, the FDX1 protein level increased. We preliminarily hypothesised that the RNA o8G modification can promote cuproptosis, but further experiments are needed to confirm this finding. Finally, we collated the downstream regulatory relationships of circKIAA1797 in this study. In terms of circKIAA1797 regulation, we found that circKIAA1797 targets the FDX1 mRNA and reduces FDX1 protein expression and that circKIAA1797 also directly binds to the transcription factor STAT1 and inhibits LIPT1 expression by suppressing transcription; circKIAA1797 also indirectly regulates mPTP opening and closing through BAX and Bcl2. In this part of the study, “circKIAA1797-FDX1” and “circKIAA1797-STAT1-LIPT1” are two parallel lines of research derived from the idea that circRNAs can be regulated downstream at both the RNA level and the protein level. The “circKIAA1797-mPTP” line of research highlights the diverse regulation of circRNAs from a more macroscopic perspective (organelle level). Through molecular docking and Co-IP experiments, we found that FDX1-LIPT1-DLAT combine to form a functional complex with FDX1 as the core, in which FDX1 inhibits the ability of lipoic acid to bind to DLAT by LIPT1. In summary, this study is the first to show that the FDX1-LIPT1-DLAT ternary complex can exacerbate TCA cycle disorders by inhibiting DLAT activation, that the opening and closing of the mPTP can regulate cuproptosis, and that circKIAA1797 can regulate cuproptosis through these two pathways.

In conclusion, the o8G reader YBX1 increases circKIAA1797 stability and cytoplasmic distribution, and circKIAA1797 affects cuproptosis levels by regulating the FDX1-LIPT1-DLAT ternary complex and the opening and closing of the mPTP, which ultimately promotes the development of lung cancer. These new findings not only enrich our knowledge of the regulation of circRNA expression but also provide new insights into the epigenetic mechanisms that influence the tumour cell fate.

## Electronic supplementary material

Below is the link to the electronic supplementary material.

Supplementary Material 1

Supplementary Material 2

Supplementary Material 3

Supplementary Material 4

Supplementary Material 5

Supplementary Material 6

Supplementary Material 7

## Data Availability

Sequencing raw data in this study can be found online from Sequence Read Archive (SRA) (PRJNA971588).

## Abbreviations

Act D: Actinomycin D
ANT2: Adenine Nucleotide Translocator 2
ATCC: American Type Culture Collection
AUC: Area Under Curve
AUF1: AU-rich Element Binding Protein 1
BAX: Bcl2-Associated X Protein
Bcl2: B-cell Lymphoma 2
CCK-8: Cell Counting Kit-8
ChIP: Chromatin Immunoprecipitation
ChIRP: Chromatin Isolation by RNA Purification
CLIP: Crosslinking Immunoprecipitation
circRNA: Circular RNA
Co-IP: Co-immunoprecipitation
EdU: 5-Ethynyl-2’-deoxyuridine
FISH: Fluorescence In Situ Hybridization
FDX1: Ferredoxin 1
GSH: Glutathione
IARC: International Agency for Research on Cancer
IHC: Immunohistochemistry
IF: Immunofluorescence
Ki67: Ki-67 Antigen
LIPT1: Lipoyltransferase 1
lncRNA: Long Non-coding RNA
mPTP: mitochondrial permeability transition pore
mRNA: Messenger RNA
miRNA: Micro RNA
ncRNA: Non-coding RNA
o8G: 8-oxo-7,8-dihydroguanosine
PBS: Phosphate Buffered Saline
PVDF: Polyvinylidene Fluoride
qPCR: Quantitative Polymerase Chain Reaction
RhoA: Ras Homolog Family Member A
RIP: RNA Immunoprecipitation
RNase A: Ribonuclease A
RNase R: Ribonuclease R
ROC: Receiver Operating Characteristic
ROS: Reactive Oxygen Species
SDS: Sodium Dodecyl Sulfate
shRNA: Short Hairpin RNA
siRNA: Small Interfering RNA
STAT1: Signal Transducer and Activator of Transcription 1
TBST: Tris Buffered saline-tween
TRAP: Tagged RNA Affinity Purification
TCA: Tricarboxylic Acid
YBX1: Y-box binding protein 1
