# Supplementary material for: o8G-modified circKIAA1797 promotes lung cancer development by inhibiting cuproptosis
Source: J Exp Clin Cancer Res. 2025 Apr 2;44:110. doi: 10.1186/s13046-025-03365-z (PMC11963662; doi:10.1186/s13046-025-03365-z)

Full Uncropped Gels

Figure 1L

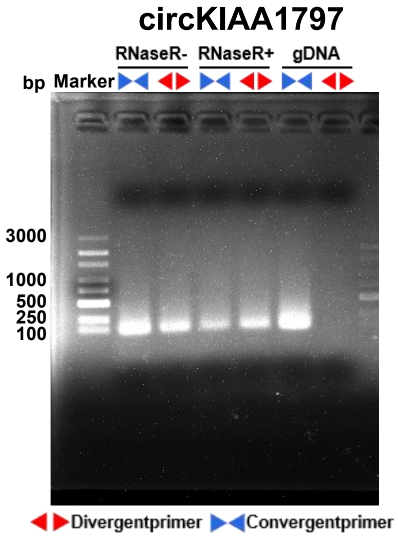

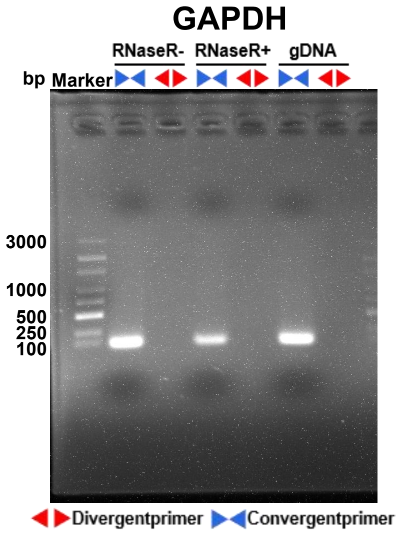

Figure 2B

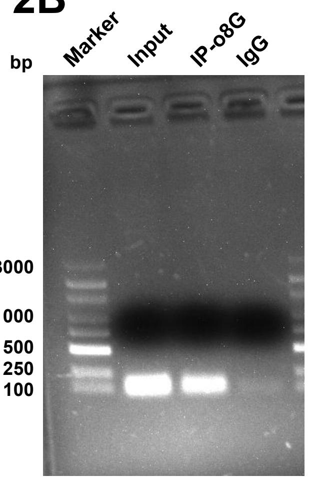

Figure 2K

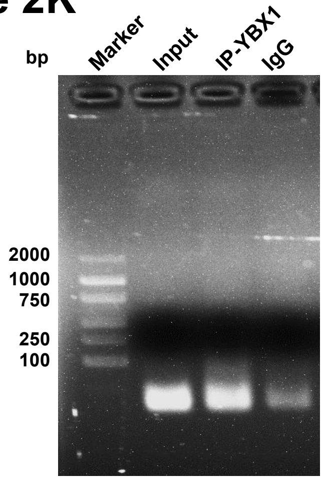

Figure 5E

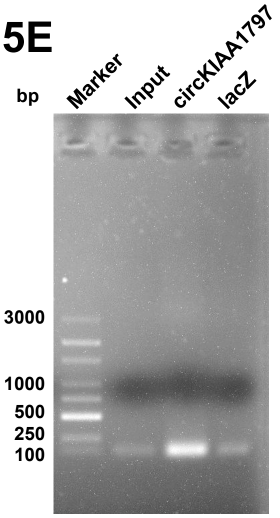

Figure 5I

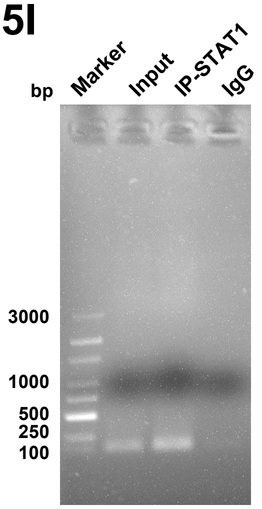

Full Blots Images

Figure 5G

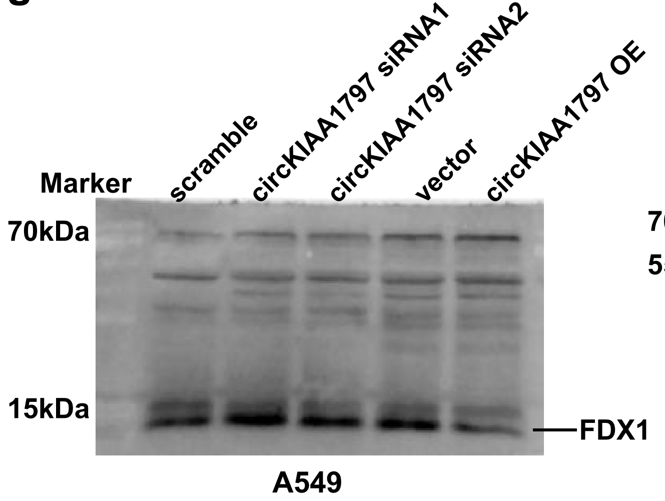

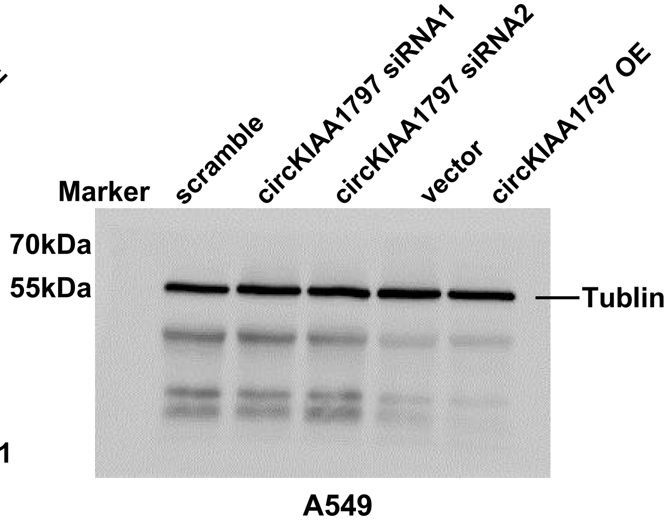

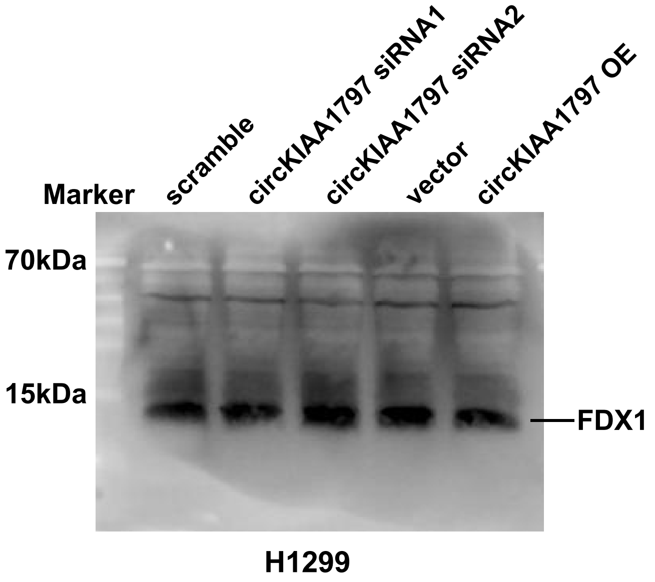

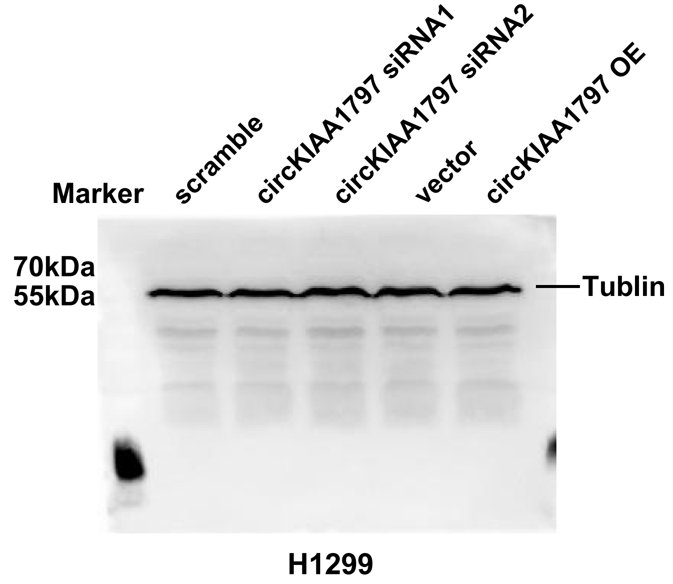

Figure 5K

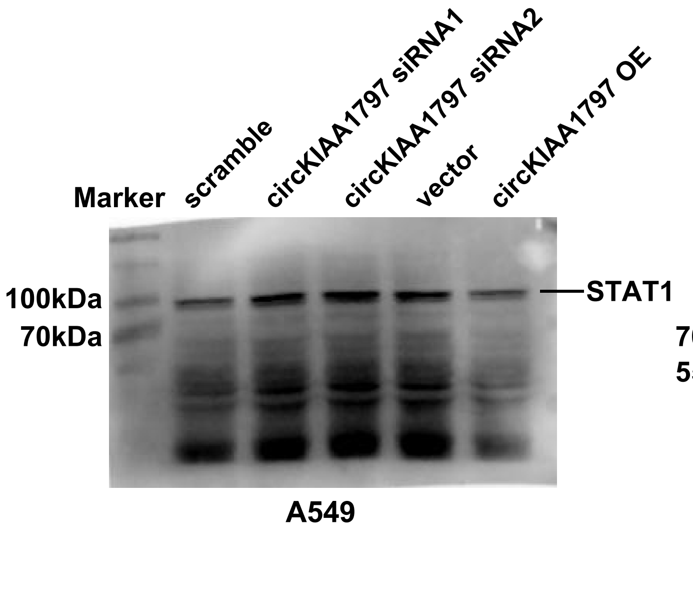

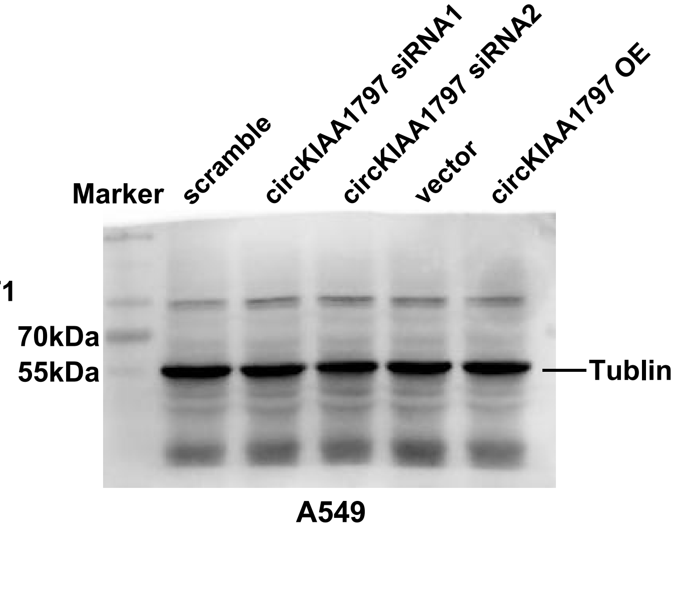

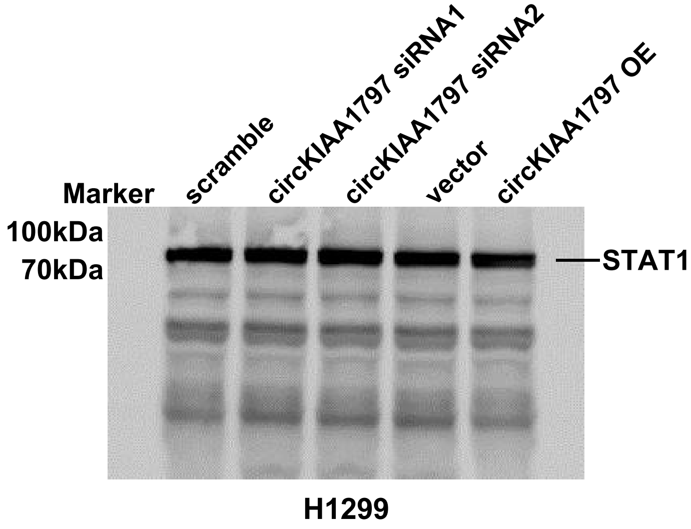
Marker
scramble
circKIAA1797 siRNA1
circKIAA1797 siRNA2
vector
circKIAA1797 OE
100kDa
70kDa
STAT1
H1299

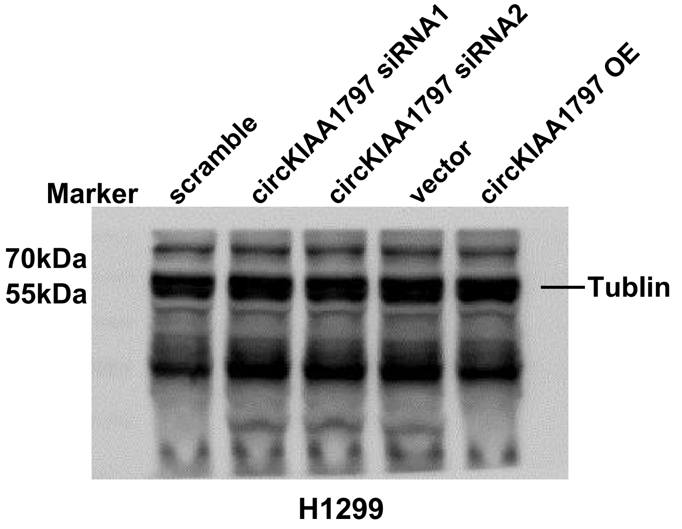
Marker
scramble
circKIAA1797 siRNA1
circKIAA1797 siRNA2
vector
circKIAA1797 OE
70kDa
55kDa
Tublin
H1299

Figure 5O

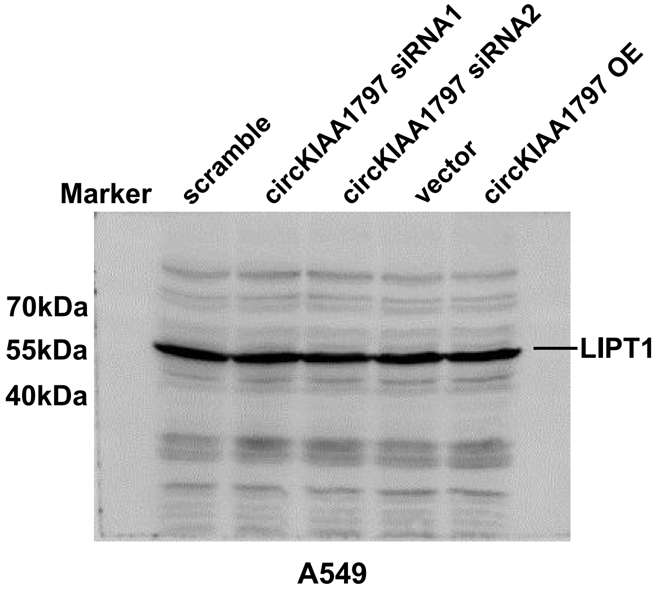
Marker
scramble
circKIAA1797 siRNA1
circKIAA1797 siRNA2
vector
circKIAA1797 OE
70kDa
55kDa
40kDa
LIPT1
A549

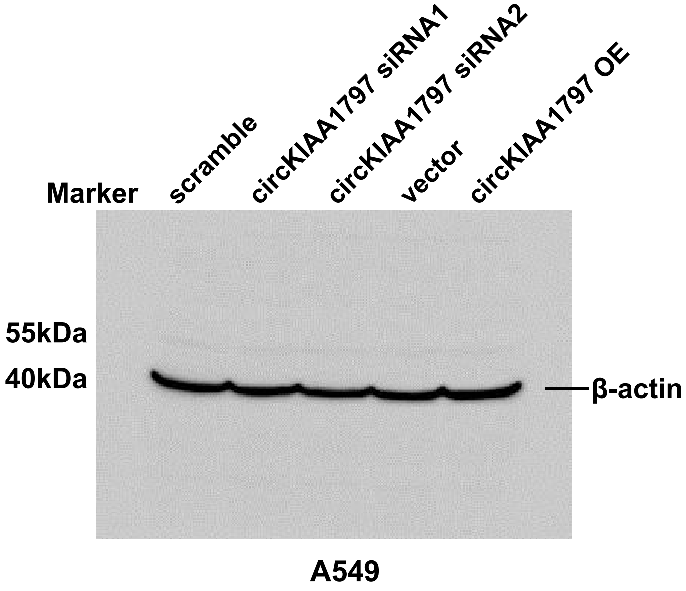
Marker
scramble
circKIAA1797 siRNA1
circKIAA1797 siRNA2
vector
circKIAA1797 OE
55kDa
40kDa
β-actin
A549

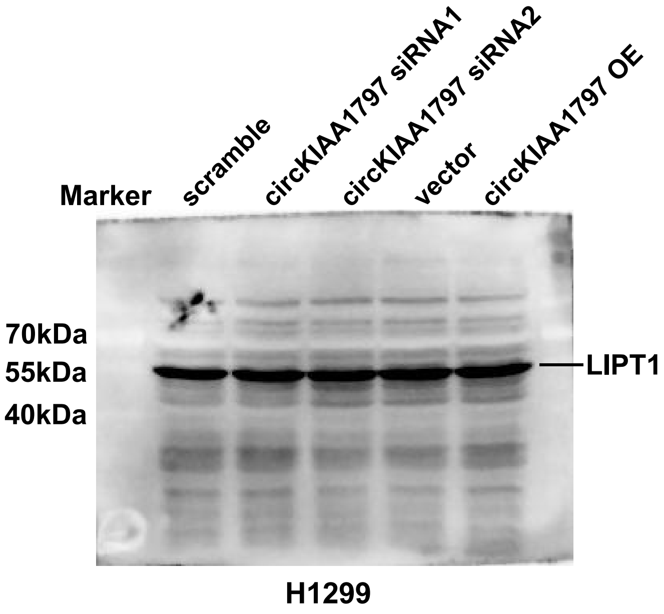
Marker
scramble
circKIAA1797 siRNA1
circKIAA1797 siRNA2
vector
circKIAA1797 OE
70kDa
55kDa
40kDa
LIPT1
H1299

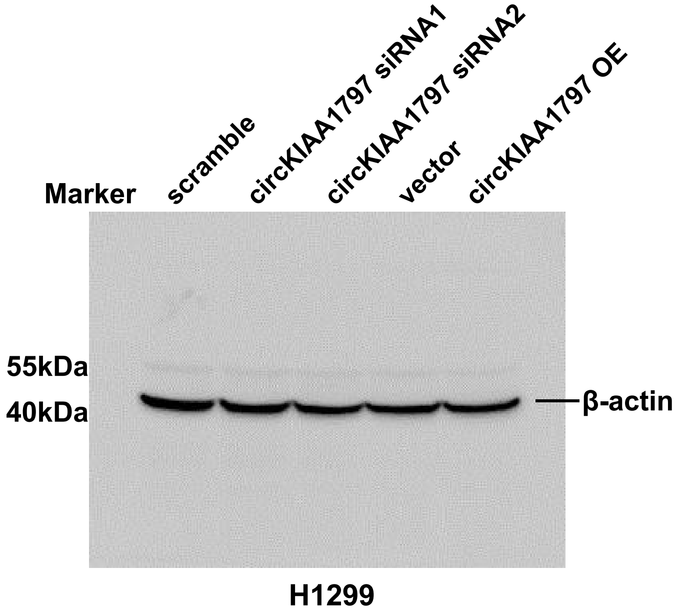
Marker
scramble
circKIAA1797 siRNA1
circKIAA1797 siRNA2
vector
circKIAA1797 OE
55kDa
40kDa
β-actin
H1299

Figure 6C

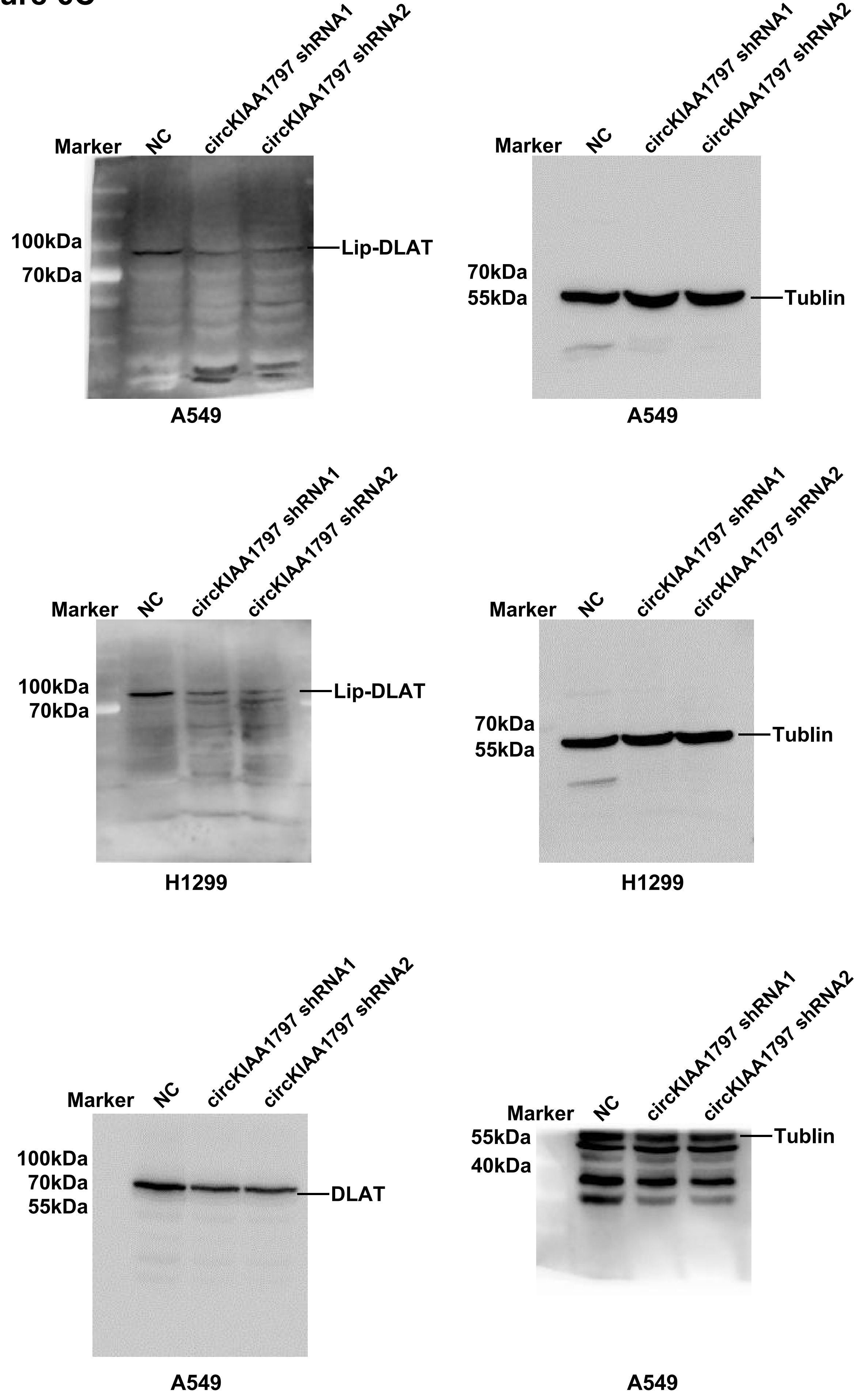

Marker
NC
circKIAA1797 shRNA1
circKIAA1797 shRNA2
100kDa
70kDa
Lip-DLAT
A549
55kDa
Tublin
H1299
DLAT
40kDa

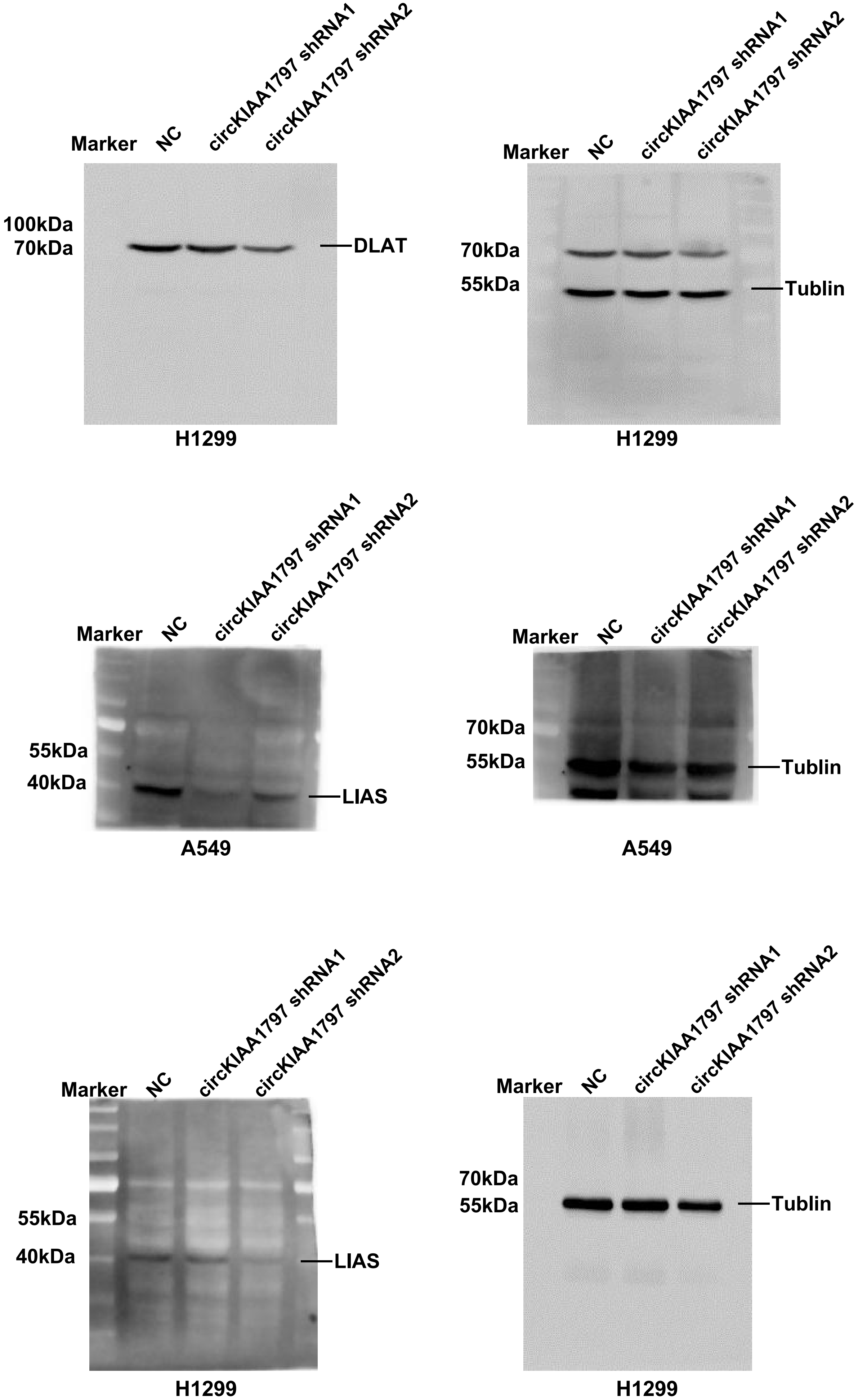
Marker
NC
circKIAA1797 shRNA1
circKIAA1797 shRNA2
100kDa
70kDa
DLAT
H1299
Marker
NC
circKIAA1797 shRNA1
circKIAA1797 shRNA2
70kDa
55kDa
Tublin
H1299
Marker
NC
circKIAA1797 shRNA1
circKIAA1797 shRNA2
55kDa
40kDa
LIAS
A549
Marker
NC
circKIAA1797 shRNA1
circKIAA1797 shRNA2
70kDa
55kDa
Tublin
A549
Marker
NC
circKIAA1797 shRNA1
circKIAA1797 shRNA2
55kDa
40kDa
LIAS
H1299
Marker
NC
circKIAA1797 shRNA1
circKIAA1797 shRNA2
70kDa
55kDa
Tublin
H1299

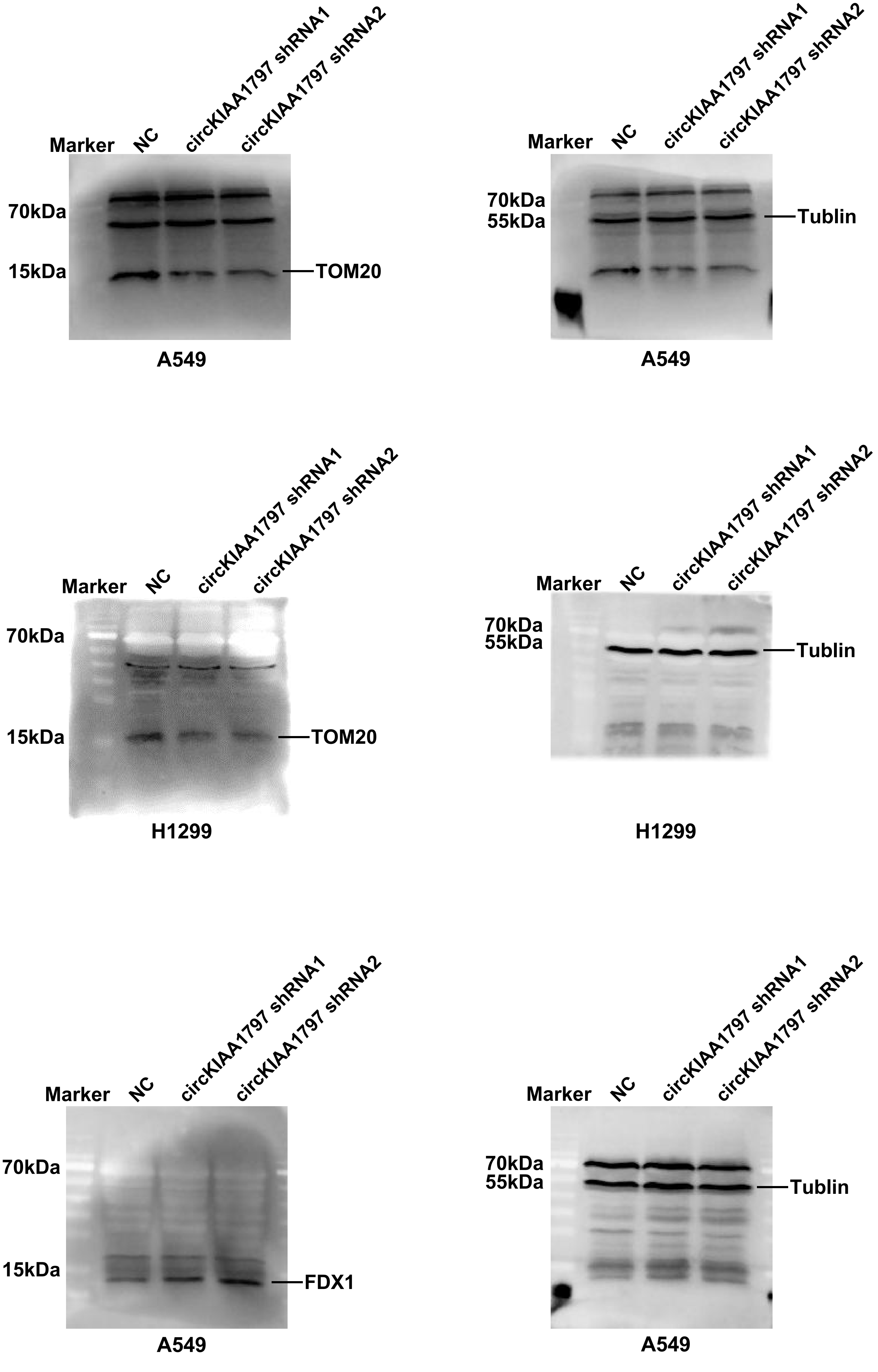

Marker
NC
circKIAA1797 shRNA1
circKIAA1797 shRNA2
70kDa
15kDa
TOM20
A549
Marker
NC
circKIAA1797 shRNA1
circKIAA1797 shRNA2
70kDa
55kDa
Tublin
A549
Marker
NC
circKIAA1797 shRNA1
circKIAA1797 shRNA2
70kDa
15kDa
TOM20
H1299
Marker
NC
circKIAA1797 shRNA1
circKIAA1797 shRNA2
70kDa
55kDa
Tublin
H1299
Marker
NC
circKIAA1797 shRNA1
circKIAA1797 shRNA2
70kDa
15kDa
FDX1
A549
Marker
NC
circKIAA1797 shRNA1
circKIAA1797 shRNA2
70kDa
55kDa
Tublin
A549

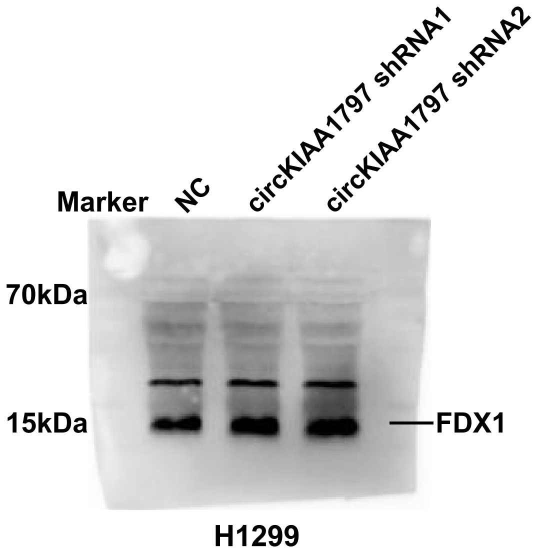
Marker
NC
circKIAA1797 shRNA1
circKIAA1797 shRNA2
70kDa
15kDa
FDX1
H1299

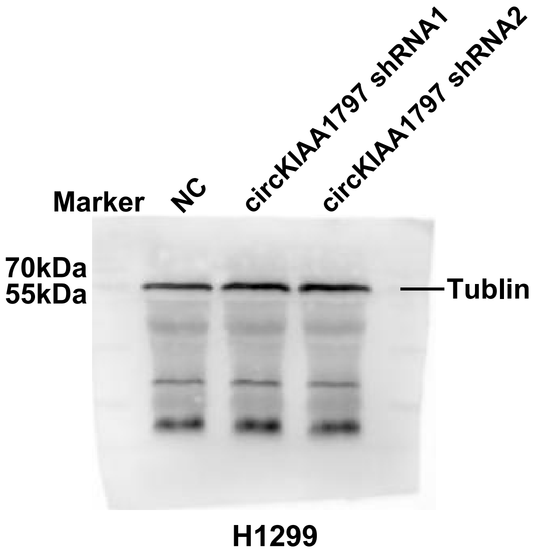
Marker
NC
circKIAA1797 shRNA1
circKIAA1797 shRNA2
70kDa
55kDa
Tublin
H1299

Figure 6J

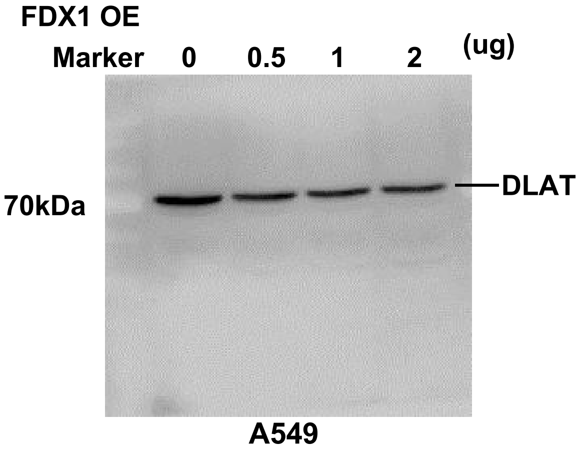
FDX1 OE
Marker
0
0.5
1
2
(ug)
70kDa
DLAT
A549

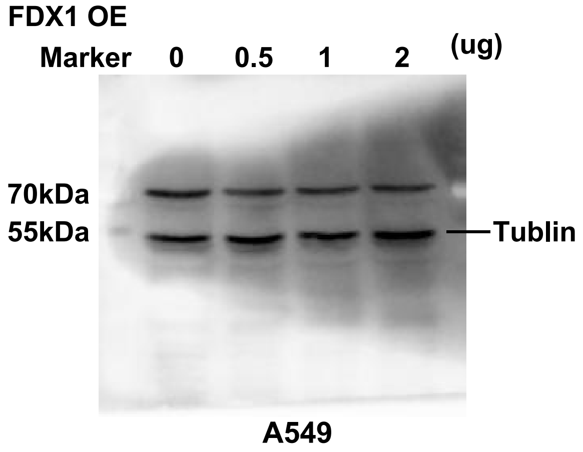
FDX1 OE
Marker
0
0.5
1
2
(ug)
70kDa
55kDa
Tublin
A549

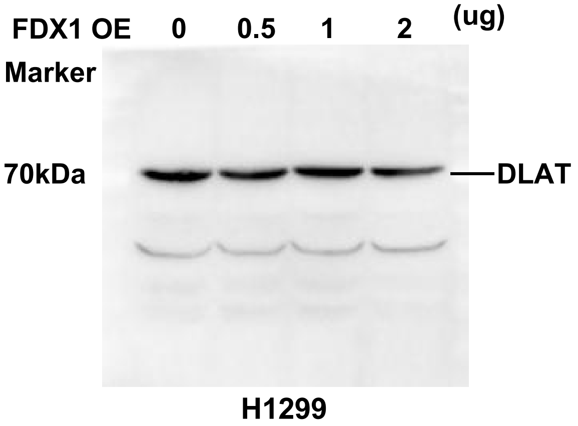
FDX1 OE
0
0.5
1
2
(ug)
Marker
70kDa
DLAT
H1299

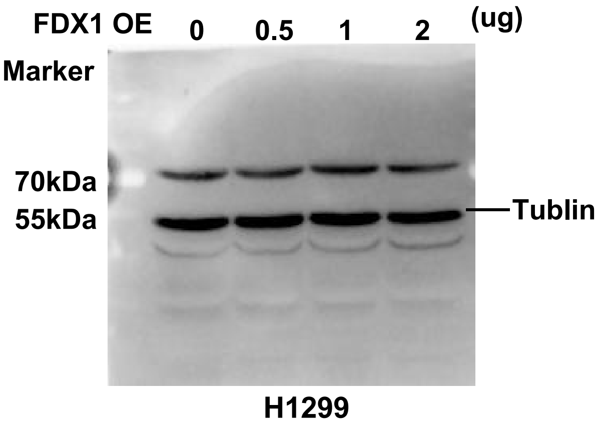
FDX1 OE
0
0.5
1
2
(ug)
Marker
70kDa
55kDa
Tublin
H1299

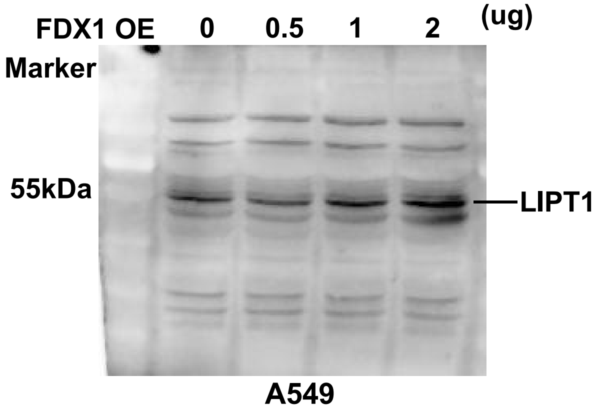

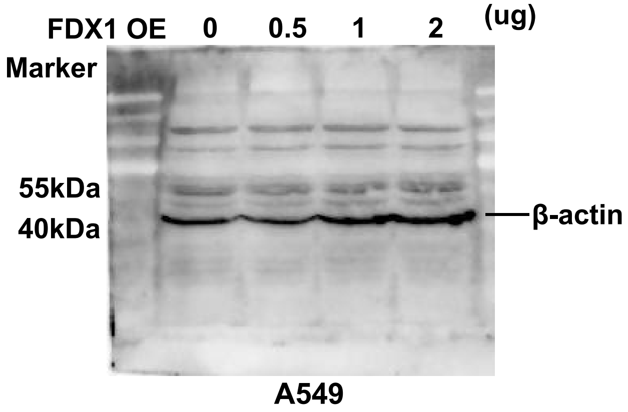

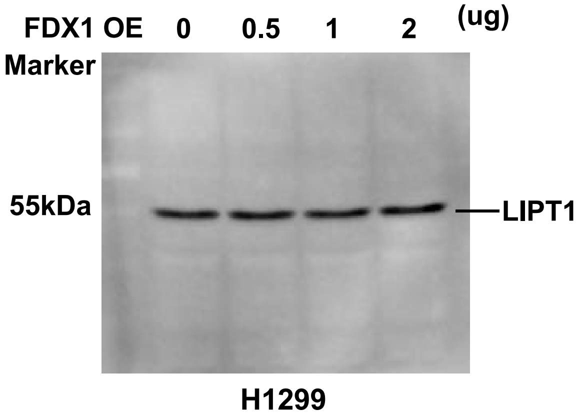

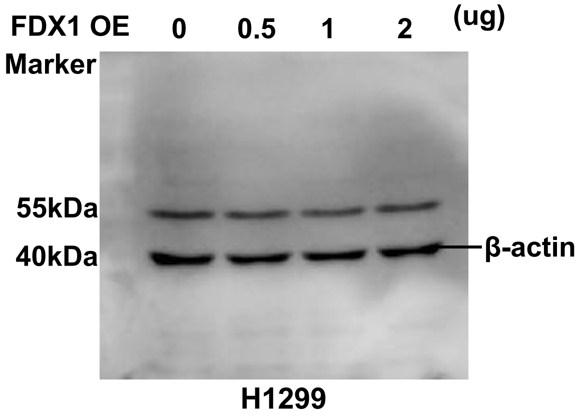

Figure 6F

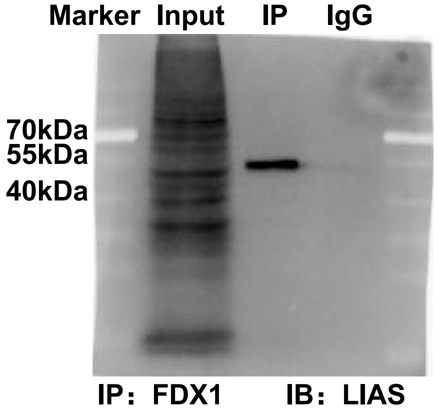

Figure 6H

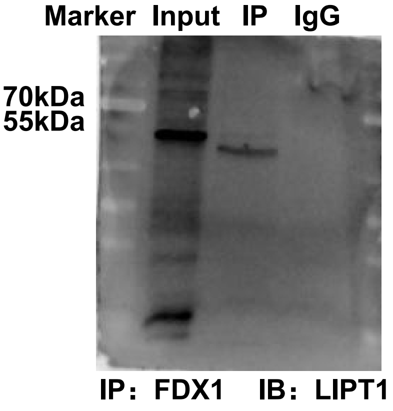

Figure 6I

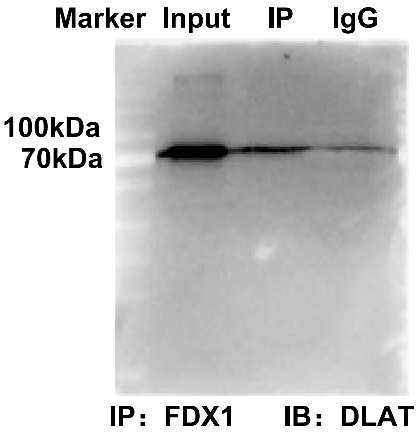

Figure 6K

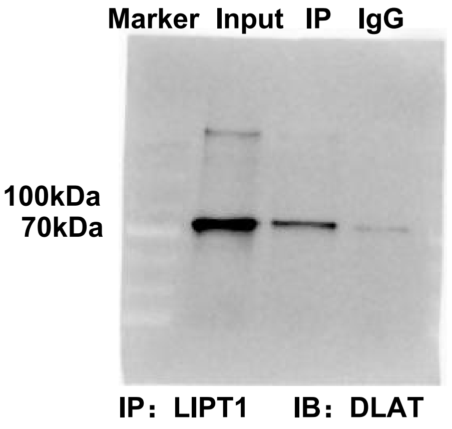

Figure 6G

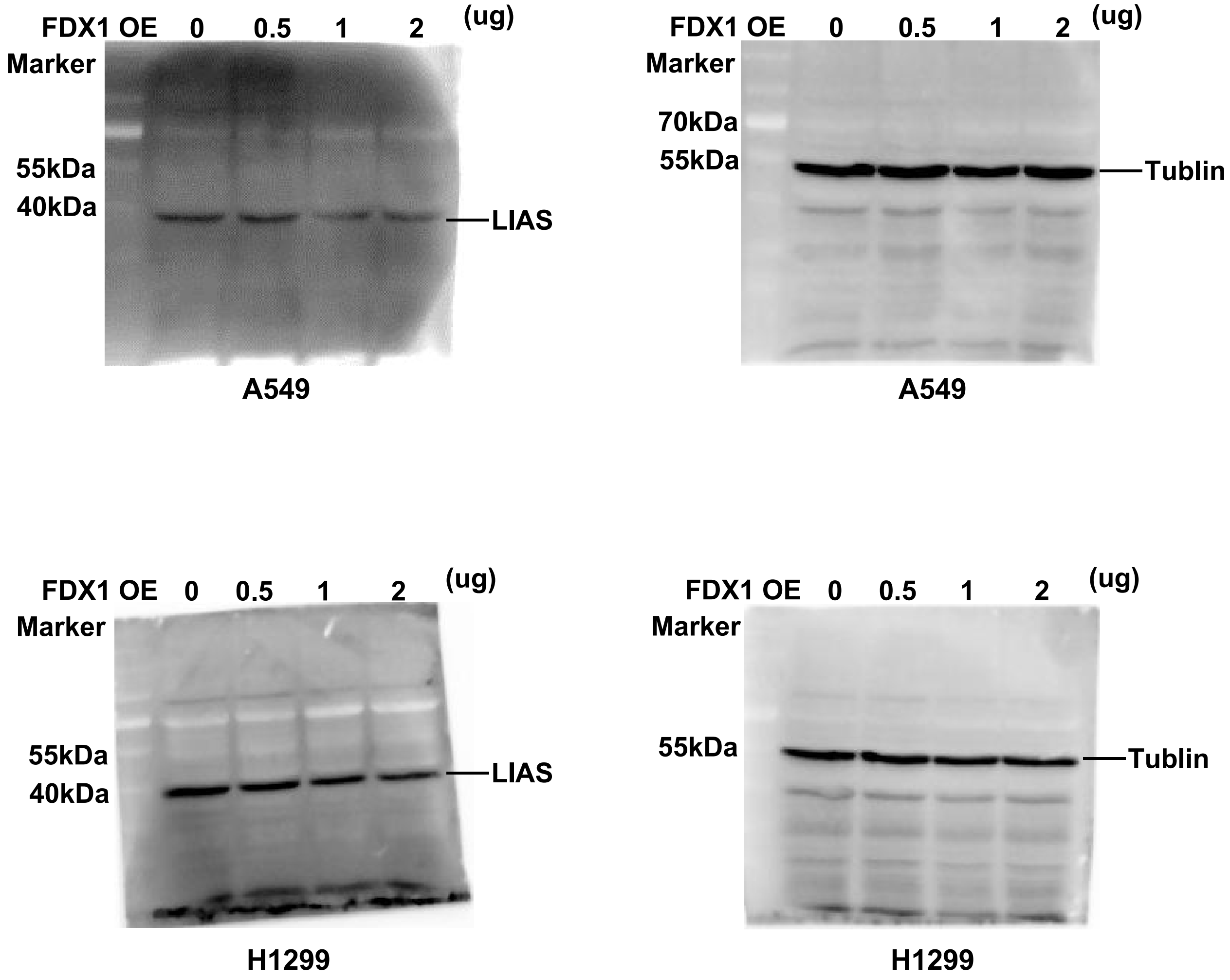

Figure 6L

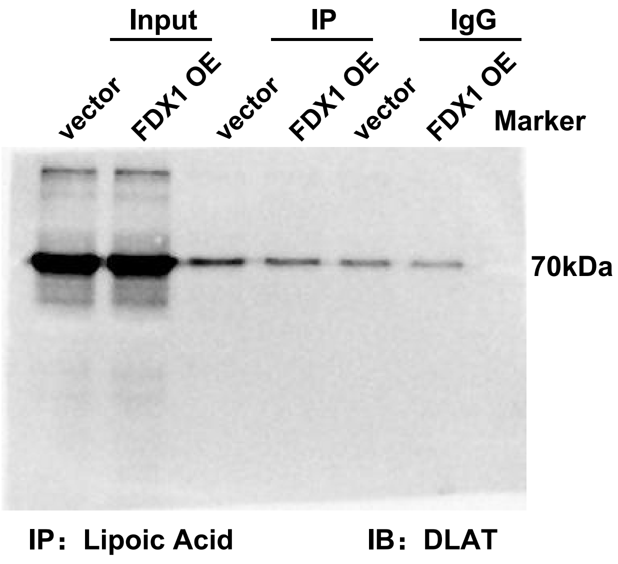

Figure 6N

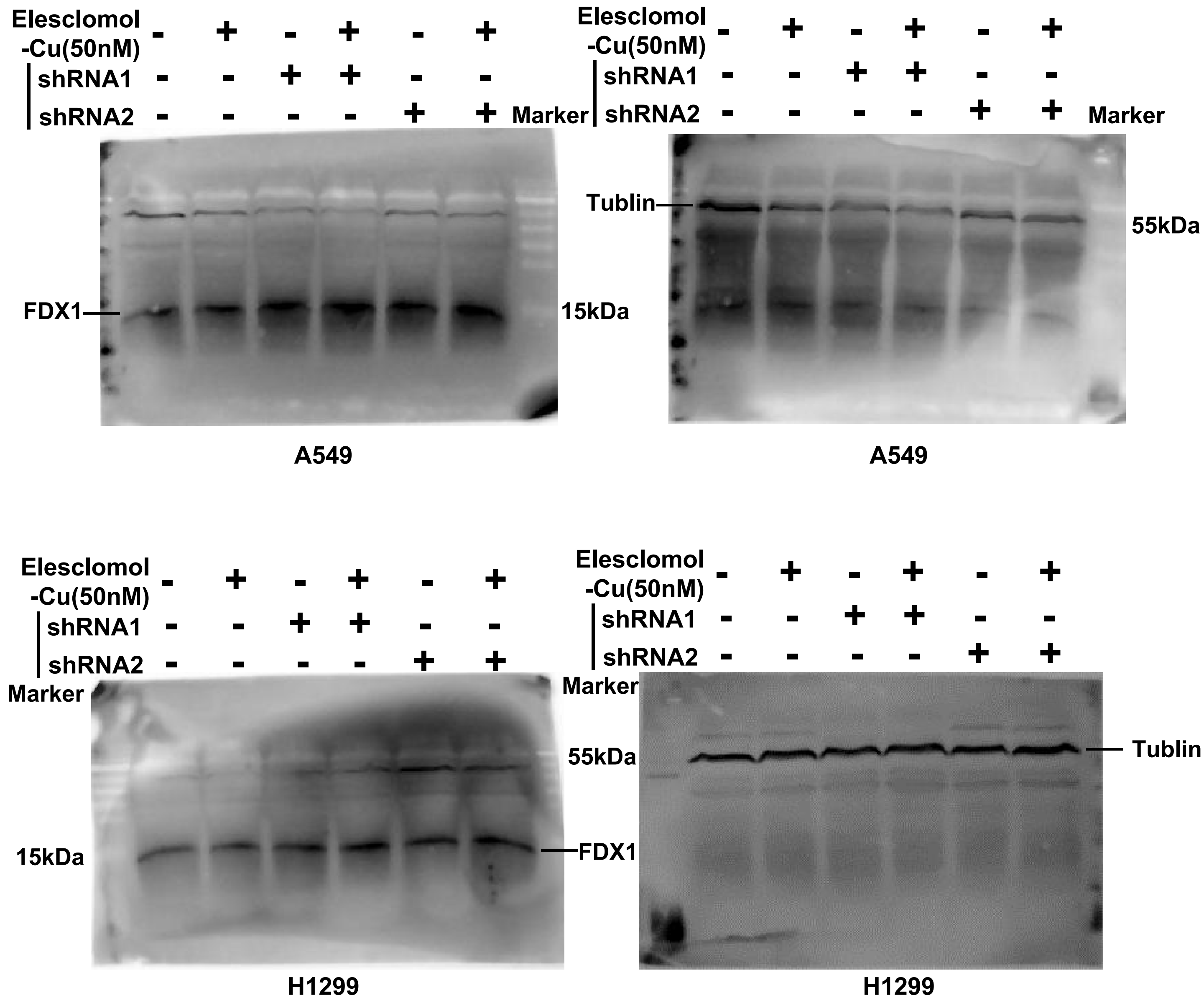

Figure 6R

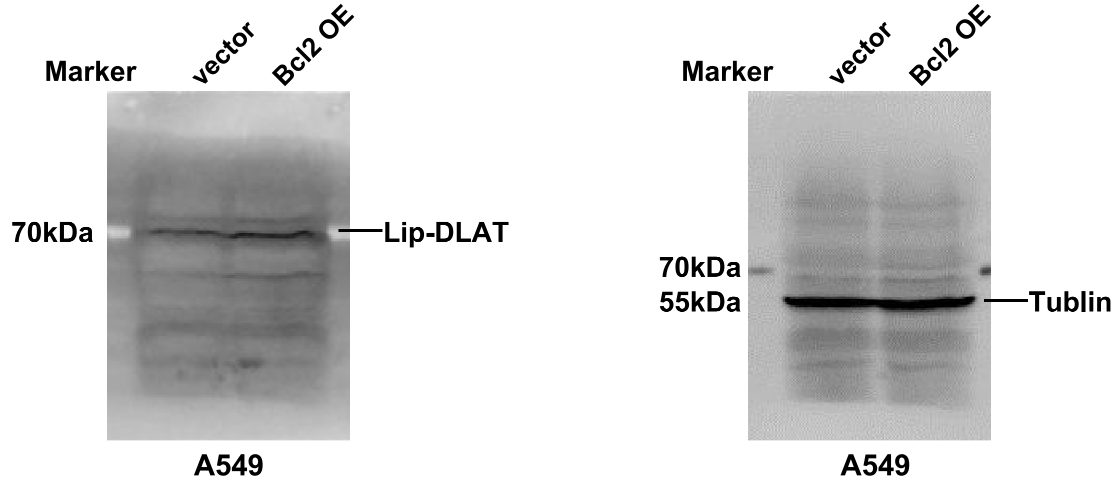

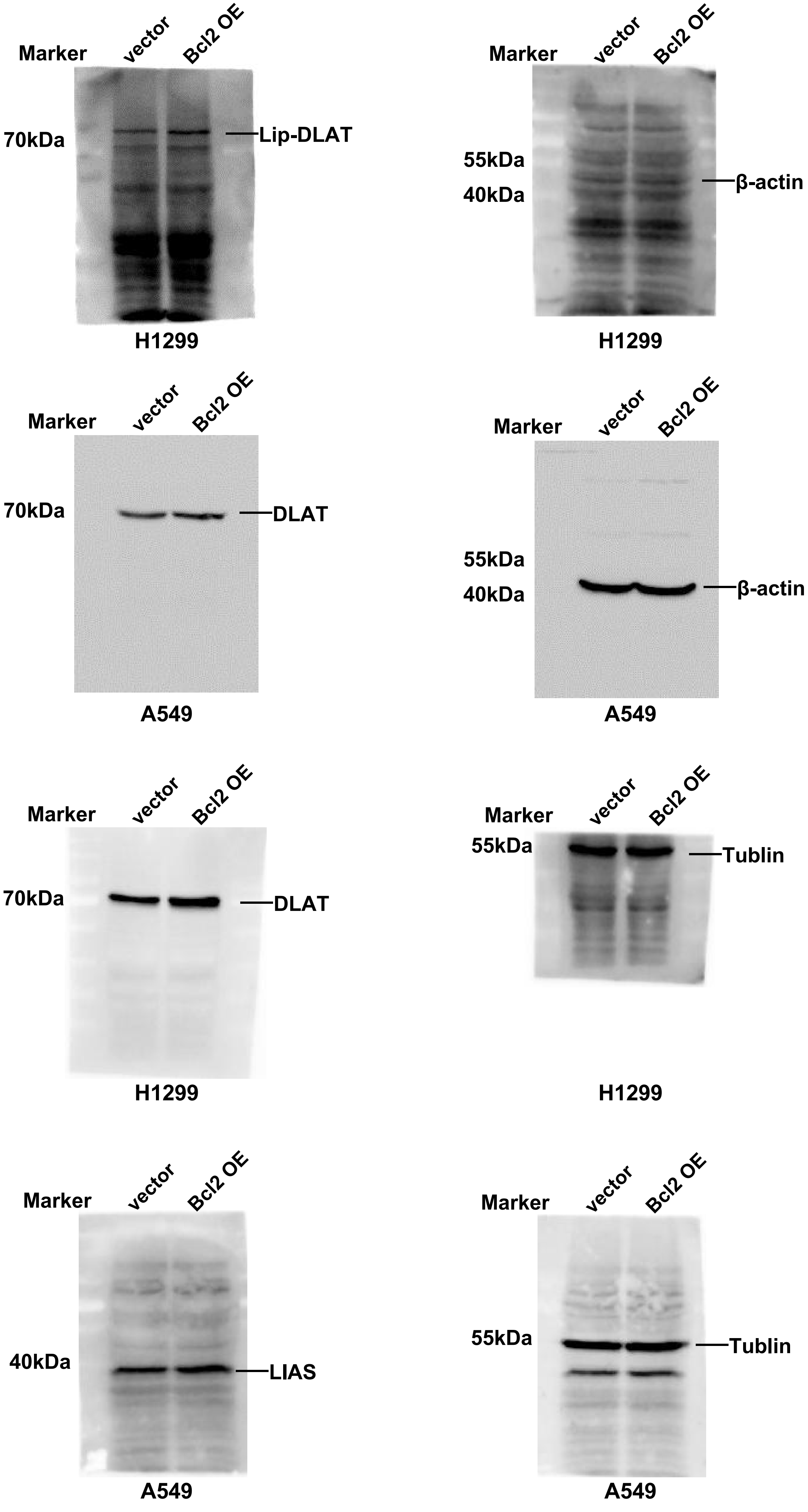
Marker
vector
Bcl2 OE
70kDa
Lip-DLAT
H1299
Marker
vector
Bcl2 OE
55kDa
40kDa
β-actin
H1299
Marker
vector
Bcl2 OE
70kDa
DLAT
A549
Marker
vector
Bcl2 OE
55kDa
40kDa
β-actin
A549
Marker
vector
Bcl2 OE
70kDa
DLAT
H1299
Marker
vector
Bcl2 OE
55kDa
Tublin
H1299
Marker
vector
Bcl2 OE
40kDa
LIAS
A549
Marker
vector
Bcl2 OE
55kDa
Tublin
A549

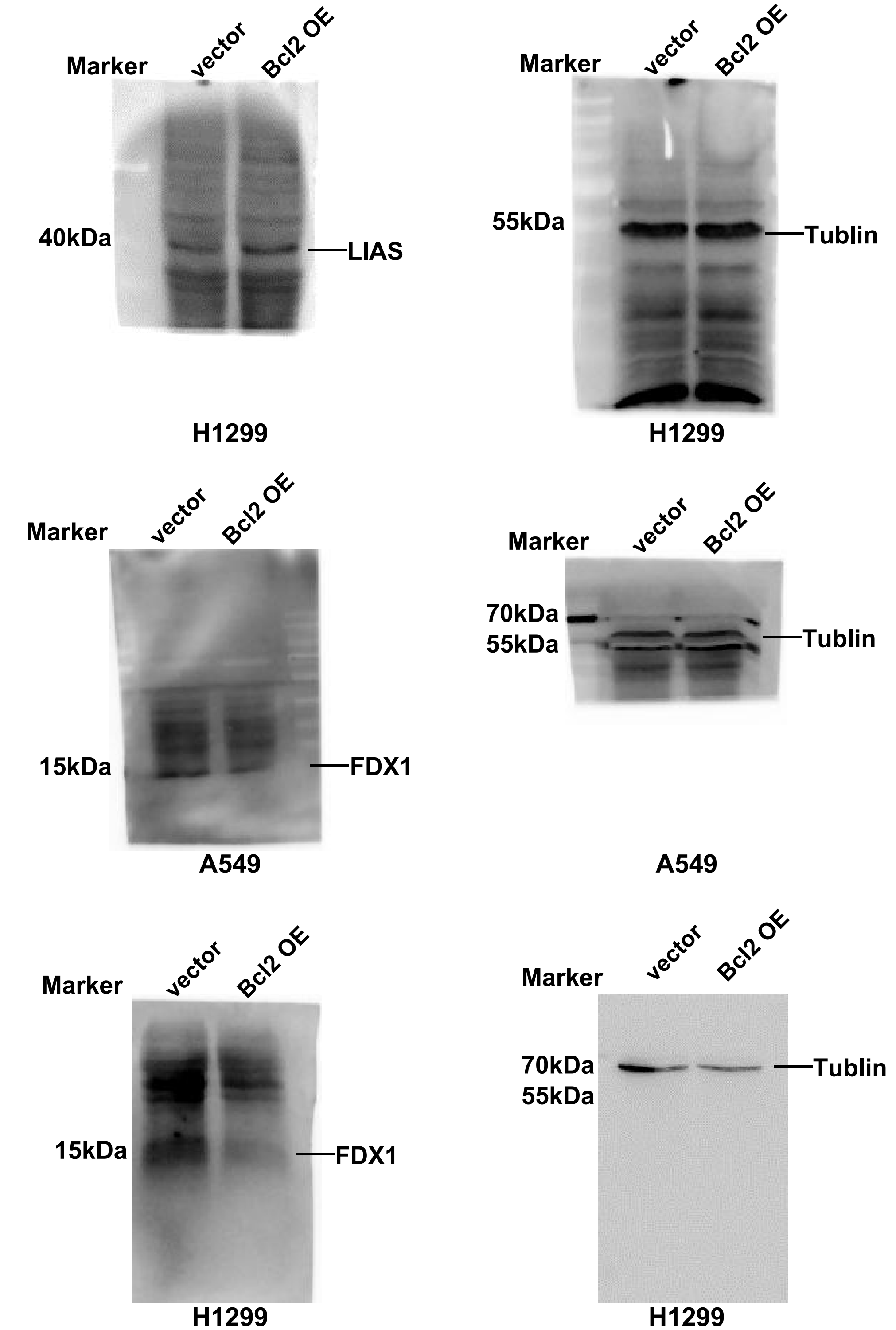
Marker
vector
Bcl2 OE
40kDa
LIAS
H1299
Marker
vector
Bcl2 OE
55kDa
Tublin
H1299
Marker
vector
Bcl2 OE
15kDa
FDX1
A549
Marker
vector
Bcl2 OE
70kDa
55kDa
Tublin
A549
Marker
vector
Bcl2 OE
15kDa
FDX1
H1299
Marker
vector
Bcl2 OE
70kDa
55kDa
Tublin
H1299

Figure 6O

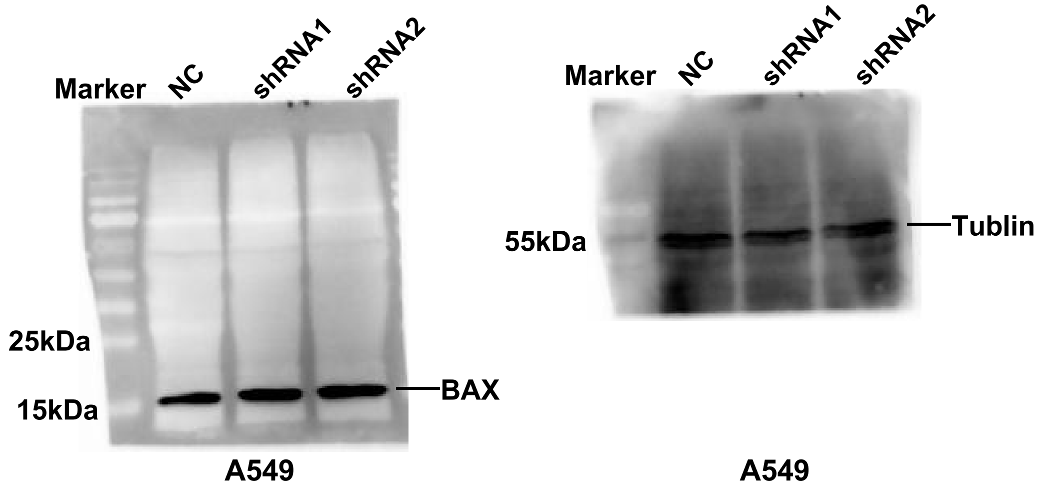
Marker
NC
shRNA1
shRNA2
25kDa
15kDa
BAX
A549
Marker
NC
shRNA1
shRNA2
55kDa
Tublin
A549

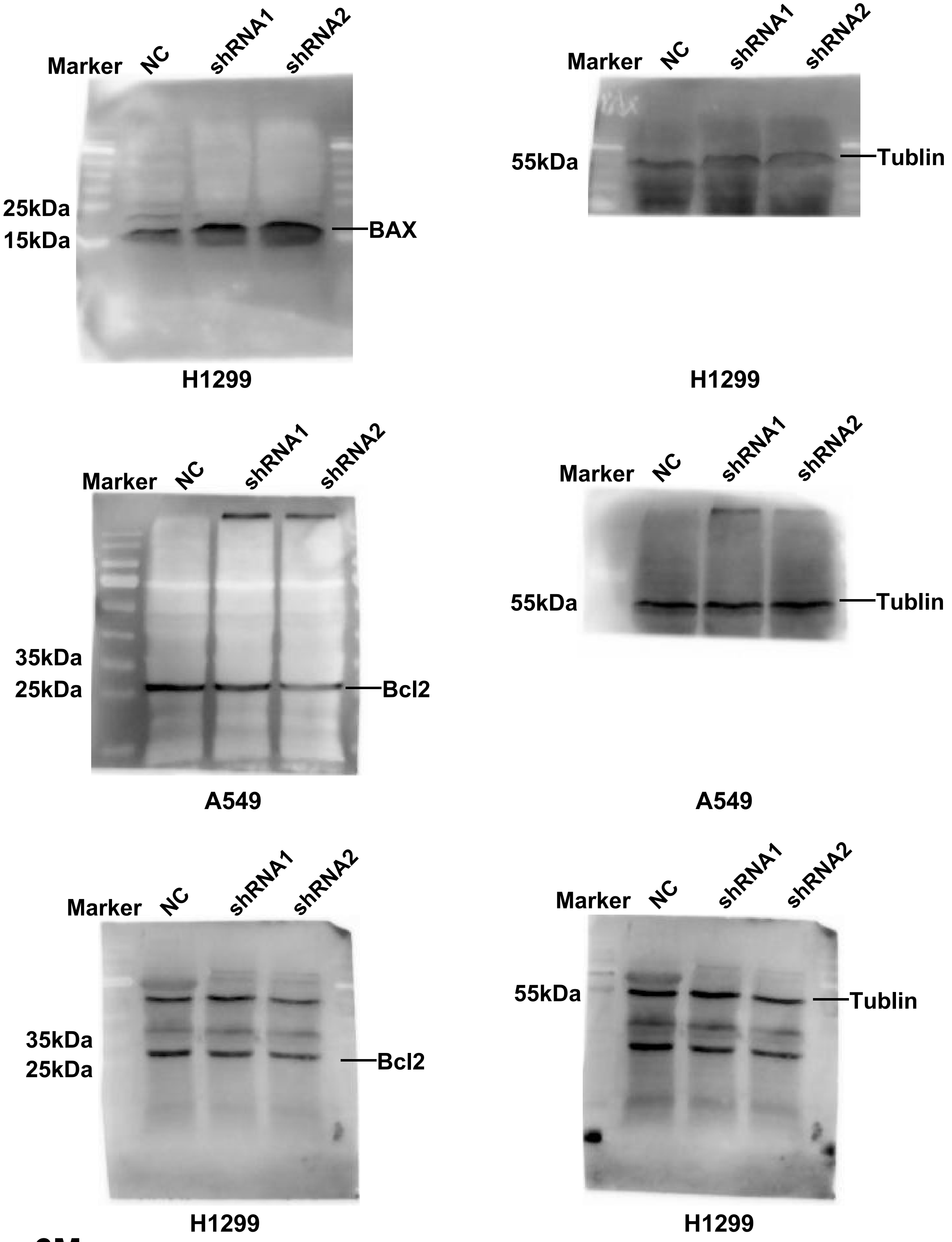
Marker
NC
shRNA1
shRNA2
25kDa
15kDa
BAX
H1299
Marker
NC
shRNA1
shRNA2
55kDa
Tublin
H1299
Marker
NC
shRNA1
shRNA2
35kDa
25kDa
Bcl2
A549
Marker
NC
shRNA1
shRNA2
55kDa
Tublin
A549
Marker
NC
shRNA1
shRNA2
35kDa
25kDa
Bcl2
H1299
Marker
NC
shRNA1
shRNA2
55kDa
Tublin
H1299

Figure 6M

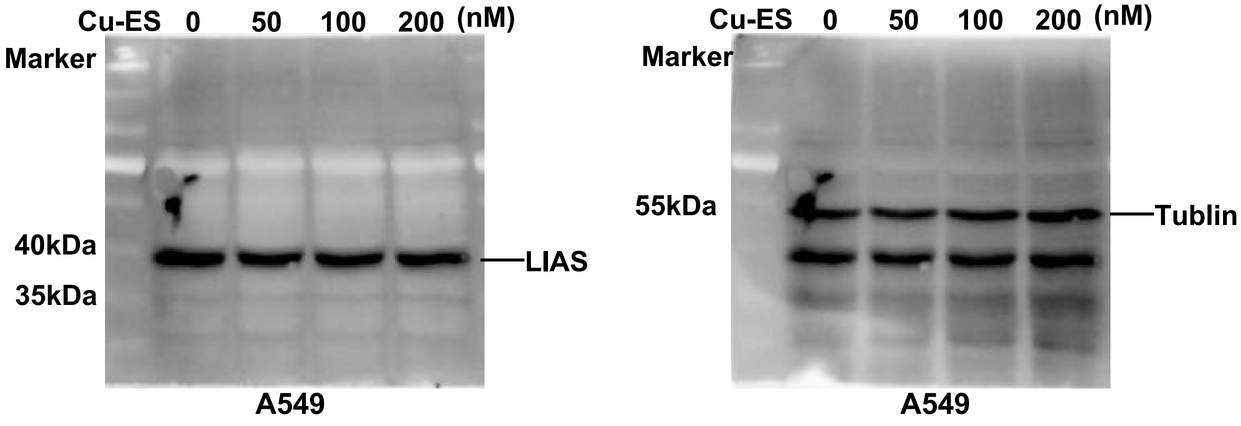
Cu-ES 0 50 100 200 (nM)
Marker
40kDa
35kDa
LIAS
A549
Cu-ES 0 50 100 200 (nM)
Marker
55kDa
Tublin
A549

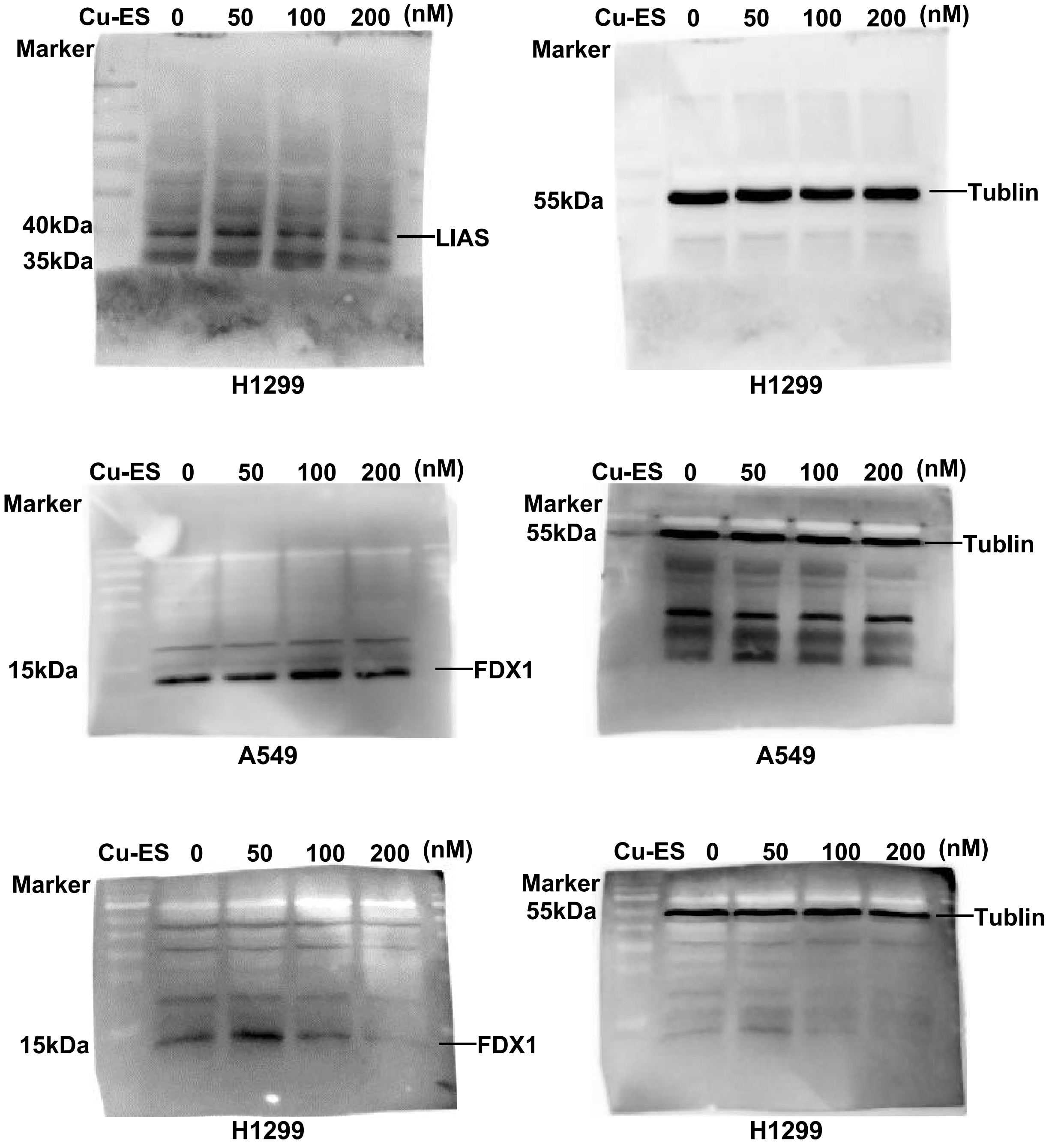
Cu-ES 0 50 100 200 (nM)
Marker
40kDa
35kDa
LIAS
H1299
Cu-ES 0 50 100 200 (nM)
Marker
55kDa
Tublin
H1299
Cu-ES 0 50 100 200 (nM)
Marker
15kDa
FDX1
A549
Cu-ES 0 50 100 200 (nM)
Marker
55kDa
Tublin
A549
Cu-ES 0 50 100 200 (nM)
Marker
15kDa
FDX1
H1299
Cu-ES 0 50 100 200 (nM)
Marker
55kDa
Tublin
H1299

Figure 7A

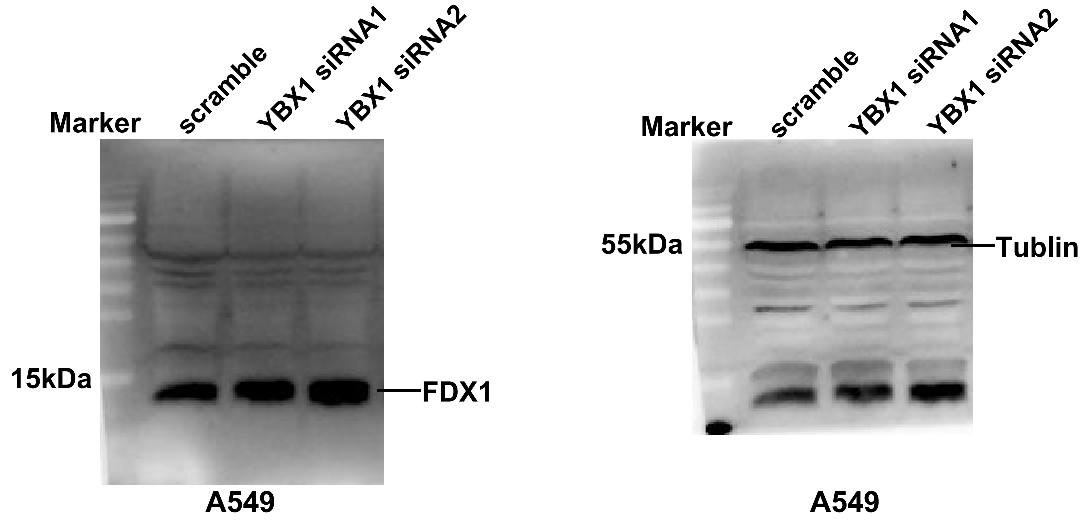
Marker
scramble
YBX1 siRNA1
YBX1 siRNA2
15kDa
FDX1
A549
Marker
scramble
YBX1 siRNA1
YBX1 siRNA2
55kDa
Tublin
A549

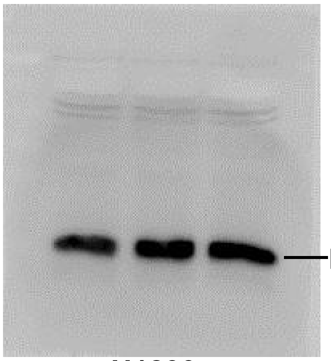
Marker
scramble
YBX1 siRNA1
YBX1 siRNA2
15kDa
FDX1
H1299

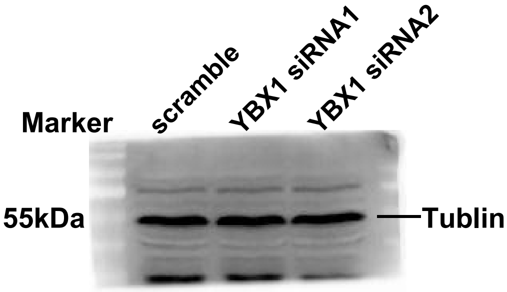
Marker
scramble
YBX1 siRNA1
YBX1 siRNA2
55kDa
Tublin
H1299

Figure 7C

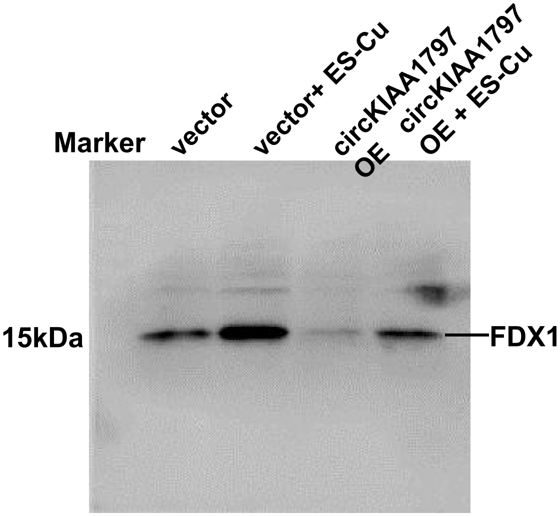
Marker
vector
vector+ ES-Cu
circKIAA1797 OE
circKIAA1797 OE + ES-Cu
15kDa
FDX1
A549

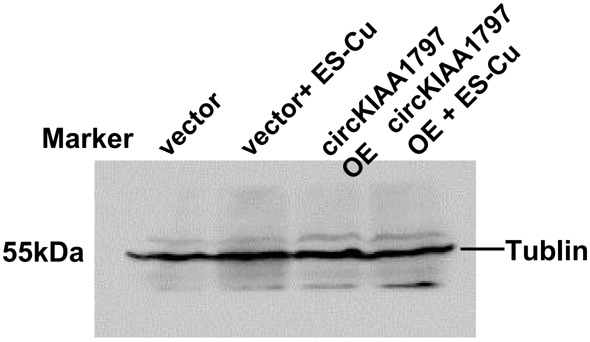
Marker
vector
vector+ ES-Cu
circKIAA1797 OE
circKIAA1797 OE + ES-Cu
55kDa
Tublin
A549

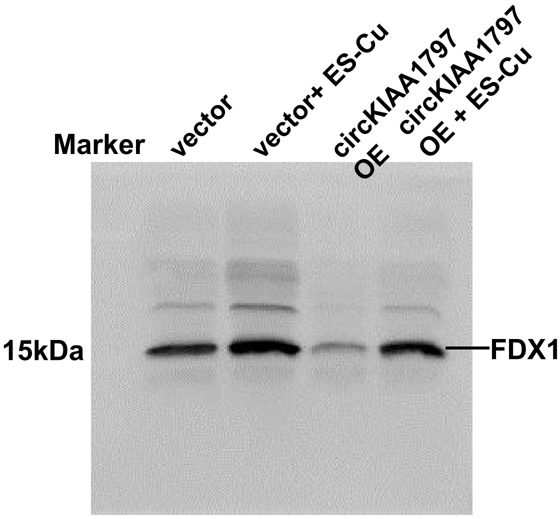
Marker
vector
vector+ ES-Cu
circKIAA1797 OE
circKIAA1797 OE + ES-Cu
15kDa
FDX1
H1299

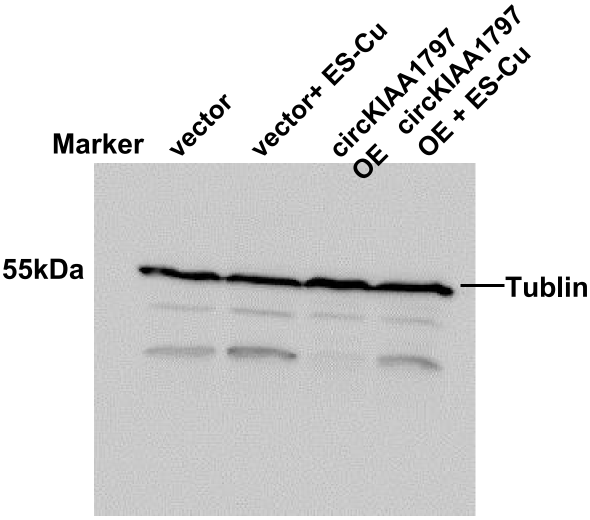
Marker
vector
vector+ ES-Cu
circKIAA1797 OE
circKIAA1797 OE + ES-Cu
55kDa
Tublin
H1299

Supplementary Figure 5B

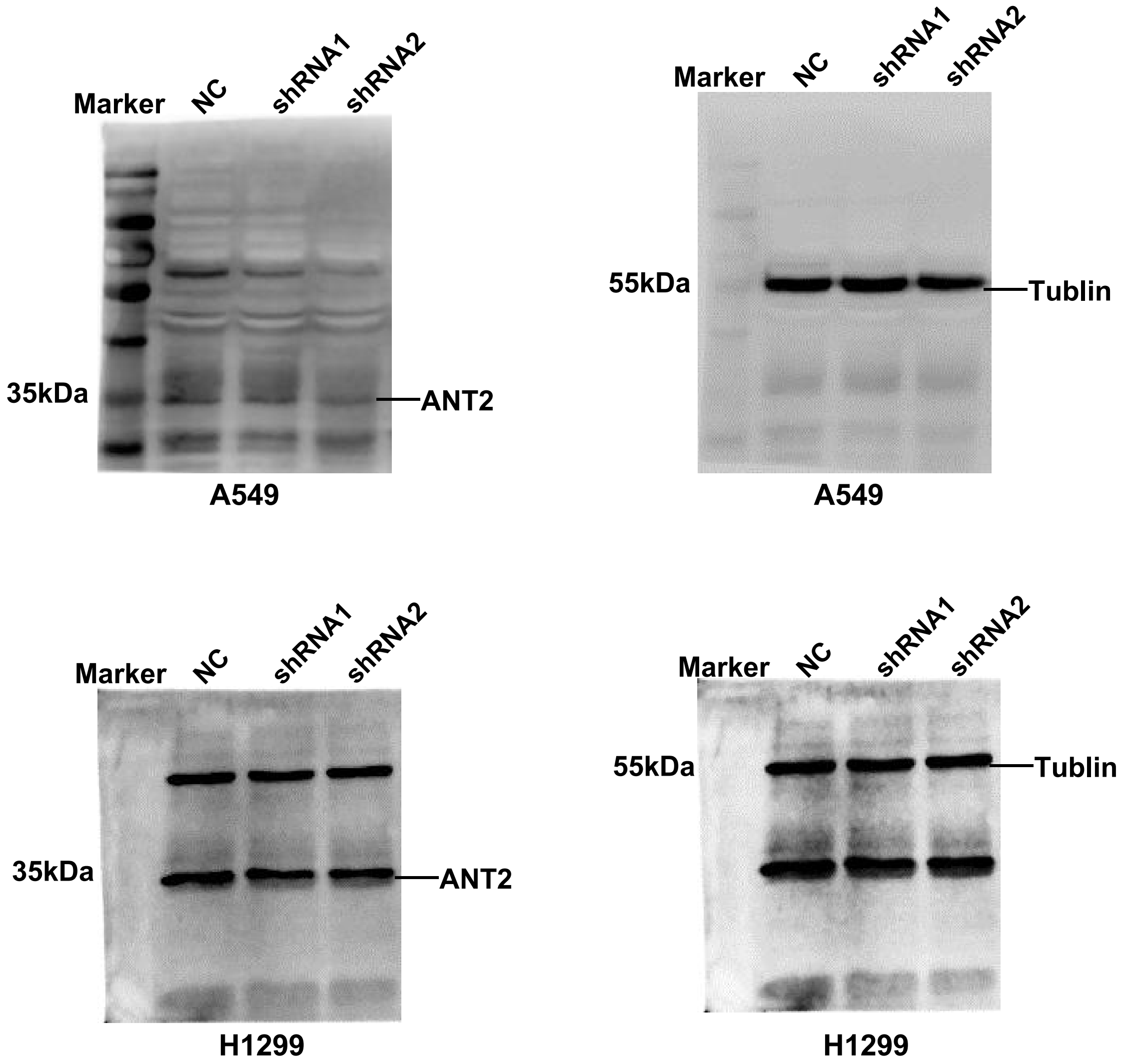

Supplement: Supplementary file 3 — Supplementary Material 3 [file 13046_2025_3365_MOESM3_ESM.pdf]
