# Supplementary material for: o8G-modified circKIAA1797 promotes lung cancer development by inhibiting cuproptosis
Source: J Exp Clin Cancer Res. 2025 Apr 2;44:110. doi: 10.1186/s13046-025-03365-z (PMC11963662; doi:10.1186/s13046-025-03365-z)
Supplement: Supplementary file 4 — Supplementary Material 4 [file 13046_2025_3365_MOESM4_ESM.docx]

**Table S1. Nucleic acid sequence information of the siRNA and PCR primer**

| **qPCR primer name** | **Sequence (5’-3’)** |
| --- | --- |
| circKIAA1797 (Forward) | TGAATGAATGCACCAAGCCT |
| circKIAA1797 (Reverse) | GGTTCCAGTGGTCTTCCCAAT |
| KIAA1797 (Forward) | CTGACAGCGTTTTTCCAGCAG |
| KIAA1797 (Reverse) | GGGCTAGTCGGAGTTTAGCAT |
| circKIAA1797 convergent (Forward) | CACTTTGCGCTTGCTGACAT |
| circKIAA1797 convergent (Reverse) | TCCCATTGGACTTCCTTGCC |
| GAPDH (Forward) | ATCAATGGAAATCCCATCACCA |
| GAPDH (Reverse) | GACTCCACGACGTACTCAGCG |
| U6 (Forward) | GGAACGATACAGAGAAGATTAGC |
| U6 (Reverse) | TGGAACGCTTCACGAATTTGCG |
| β-actin (Forward) | GCACAGAGCCTCGCCTT |
| β-actin (Reverse) | GTTGTCGACGACGAGCG |
| FDX1 (Forward) | TTCAACCTGTCACCTCATCTTTG |
| FDX1 (Reverse) | TGCCAGATCGAGCATGTCATT |
| LIPT1 (Forward) | GCTGGATGTGCAGGCTACC |
| LIPT1 (Reverse) | GCAATGGTGATAGGCAGTAGTC |
| BCL2 (Forward) | TTGCCAGCCGGAACCTATG |
| BCL2 (Reverse) | CGAAGGCGACCAGCAATGATA |
| STAT1 (Forward) | CAGCTTGACTCAAAATTCCTGGA |
| STAT1 (Reverse) | TGAAGATTACGCTTGCTTTTCCT |
| CLIP-circKIAA1797-1 | AGACCACTGGAACCTAT |
| CLIP-circKIAA1797-2 | TACCTAAGCTTGGTGTTC |
| CLIP-circKIAA1797-3 | AATAATACAACTACTTGGAACC |
| CLIP-circKIAA1797-4 | TCACTTTGCGCTTGCTGA |
| CLIP-circKIAA1797-5 | CCTGAACTGCAGCGTTTC |
| CLIP-circKIAA1797-6 | GGGCAAGGAAGTCCAATG |
| CLIP-circKIAA1797-7 | TGTAAGCAGAGGCCATAT |
| CLIP-circKIAA1797-8 | CAAGTGTTGAATGAATGCAC |
| CLIP-circKIAA1797-9 | ATTACAGGGTCTTCATGC |
| ChIP-DLAT-1 (Forward) | AGCTTTTCCAGAAAGCTTGGC |
| ChIP-DLAT-1 (Reverse) | CGCAGAGACGCGGGTTAAT |
| ChIP-DLAT-2 (Forward) | AATTAACCCGCGTCTCTGCG |
| ChIP-DLAT-2 (Reverse) | AGACGGGGCCTAAGAGATGA |
| ChIP-LIAS-1 (Forward) | ATAAACCACCTTCCAACGAGCA |
| ChIP-LIAS-1 (Reverse) | GCGGAGGGACTTCAATCACAT |
| ChIP-LIAS-2 (Forward) | ACGCTTGGAACGTGCAGTAA |
| ChIP-LIAS-2 (Reverse) | TCACCCCCAGTGTTTGTGTT |
| ChIP-LIPT1-1 (Forward) | GGGCCTTATGACCTTCGGTA |
| ChIP-LIPT1-1 (Reverse) | CGCAGGAGTGCTAAGTAAGGG |
| ChIP-LIPT1-2 (Forward) | TTCTCTTGCGCTTCAGGGG |
| ChIP-LIPT1-2 (Reverse) | TCGAGCTGGAAAAGTGCGTC |
| ChIP-DLD-1 (Forward) | AACATAGGCAGTAATTTCTAACCAG |
| ChIP-DLD-1 (Reverse) | ACATTTATTGGAAGTCTACCACCCA |
| ChIP-DLD-2 (Forward) | GGCCGCAGTTATCTCTCCTT |
| ChIP-DLD-2 (Reverse) | TCGACAGTAAAGCGTGACAAG |
| ChIRP-LIPT1-1 (Forward) | TCTGCGAATACCGTTTTTCCT |
| ChIRP-LIPT1-1 (Reverse) | AATGGCAGGTAACTCGAAAGGT |
| ChIRP-LIPT1-2 (Forward) | TCTGAACTGGGGCCTTATGAC |
| ChIRP-LIPT1-2 (Reverse) | GTAAGGGTCCTTCAGGGCGG |
| ChIRP-LIPT1-3 (Forward) | GCTAAGTAAGGGTCCTTCAGGG |
| ChIRP-LIPT1-3 (Reverse) | CCATCTGAACTGGGGCCTTAT |
| ChIRP-LIPT1-4 (Forward) | AAGTGAGTCCAAATGGCAGGTA |
| ChIRP-LIPT1-4 (Reverse) | CATCTGCGAATACCGTTTTTCCT |
| ChIRP-LIPT1-5 (Forward) | AAGGTGGGTGATTCTTCTGC |
| ChIRP-LIPT1-5 (Reverse) | GCTGGCTTTGTTGTTGTTTT |
| **siRNA name** | **Sequence (5’-3’)** |
| YBX1 siRNA1 (sense) | GGAGUUUGAUGUUGUUGAAGGTT |
| YBX1 siRNA2 (sense) | CAAGGAAGAUGUAUUUGUACATT |
| circKIAA1797 siRNA1 (sense) | GUCAAGCUGAGGUGCCAAATT |
| circKIAA1797 siRNA2 (sense) | CUGUCAAGCUGAGGUGCCATT |
| scramble (sense) | UUCUCCGAACGUGUCACGUTT |
| scramble (antisense) | ACGUGACACGUUCGGAGAATT |
| **Probe name** | **Sequence (5’-3’)** |
| FISH-circKIAA1797 | CACTCTGTCAAGCTGAGGTGCCAAATCTGATTCCAG |
| ChIRP-circKIAA1797-1 | GATTTGGCACCTCAGCTTGA |
| ChIRP-circKIAA1797-2 | TGTGGTTCCAAGTAGTTGTA |
| ChIRP-circKIAA1797-3 | TGCAATCAGTTTCTCCCATT |
| ChIRP-circKIAA1797-4 | GAGTGCATGAAGACCCTGTA |
| ChIRP-lacZ-1 | ACCGCATATGGTGCACTCTC |
| ChIRP-lacZ-2 | GCGAATGGCGCCTGATGCGG |
| ChIRP-lacZ-3 | GCGAATGGCGCCTGATGCGG |
| ChIRP-lacZ-4 | GCGTTACCCAACTTAATCGC |
