# Supplementary material for: o8G-modified circKIAA1797 promotes lung cancer development by inhibiting cuproptosis
Source: J Exp Clin Cancer Res. 2025 Apr 2;44:110. doi: 10.1186/s13046-025-03365-z (PMC11963662; doi:10.1186/s13046-025-03365-z)
Supplement: Supplementary file 5 — Supplementary Material 5 [file 13046_2025_3365_MOESM5_ESM.docx]

**Table S2. Clinical characteristics of the 35 pairs of lung cancer tissue samples**

| **Characteristics** | **Number of cases (n=35)** | **Percentage (%)** |
| --- | --- | --- |
| **Age (years)** |  |  |
| ≤60 | 12 | 34.29 |
| >60 | 23 | 65.71 |
| **Gender** |  |  |
| Male | 16 | 45.71 |
| Female | 19 | 54.29 |
| **Smoking history** |  |  |
| Yes | 11 | 31.43 |
| No | 24 | 68.57 |
| **Drinking history** |  |  |
| Yes | 7 | 20.00 |
| No | 28 | 80.00 |
| **Tumor size** |  |  |
| ≤3cm | 25 | 71.43 |
| >3cm | 7 | 20.00 |
| Missing | 3 | 8.57 |
| **T stage** |  |  |
| T1 | 26 | 74.28 |
| T2+T3+T4 | 6 | 17.15 |
| Missing | 3 | 8.57 |
| **N stage** |  |  |
| N0 | 28 | 80 |
| N1+N2+N3 | 4 | 11.43 |
| Missing | 3 | 8.57 |
| **M stage** |  |  |
| M0 | 31 | 88.57 |
| M1 | 1 | 2.86 |
| Missing | 3 | 8.57 |
| **TNM stage** |  |  |
| I | 26 | 68.57 |
| II+III+ IV | 8 | 22.86 |
| Missing | 3 | 8.57 |
