# Supplementary material for: o8G-modified circKIAA1797 promotes lung cancer development by inhibiting cuproptosis
Source: J Exp Clin Cancer Res. 2025 Apr 2;44:110. doi: 10.1186/s13046-025-03365-z (PMC11963662; doi:10.1186/s13046-025-03365-z)

# Supplementary Figure 1

## A

| Accession | log2FoldChange | regulation | significant | chr | exon Count | isoformName | Host gene |
|---|---|---|---|---|---|---|---|
| hsa_circ_0009133 | 2.89 | up | yes | chr4 | 6 | ENST00000296468 | MFSD8 |
| hsa_circ_0012144 | 2.72 | up | yes | chr1 | 3 | ENST00000372259 | ERI3 |
| hsa_circ_0006702 | 2.72 | up | yes | chr9 | 8 | ENST00000443024 | IARS1 |
| hsa_circ_0006956 | 2.72 | up | yes | chr10 | 2 | ENST00000372088 | CCSER2 |
| hsa_circ_0008225 | 2.72 | up | yes | chr10 | 3 | ENST00000397962 | ZMYND11 |
| hsa_circ_0001922 | 2.72 | up | yes | X | 4 | ENST00000342160 | HUWE1 |
| hsa_circ_0003295 | 2.53 | up | yes | chr9 | 4 | ENST00000380249 | KIAA1797 |
| hsa_circ_0003275 | 2.53 | up | yes | chr18 | 3 | ENST00000426216 | ATP9B |
| hsa_circ_0006168 | 2.53 | up | yes | chr4 | 3 | ENST00000649644 | CNOT6L |
| hsa_circ_0001573 | 2.31 | up | yes | chr6 | 5 | ENST00000379933 | RREB1 |

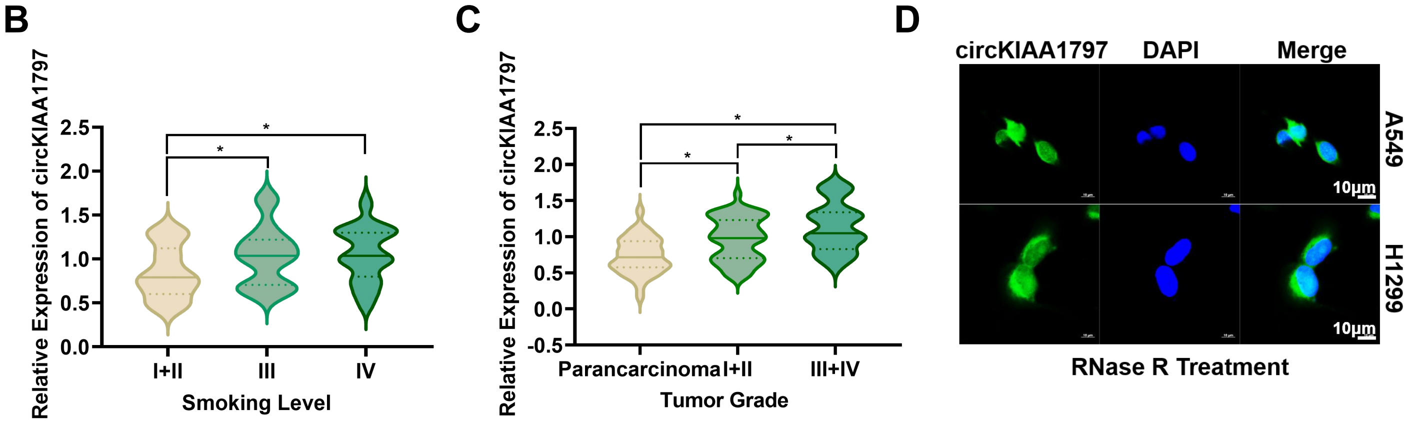

# Supplementary Figure 2

A

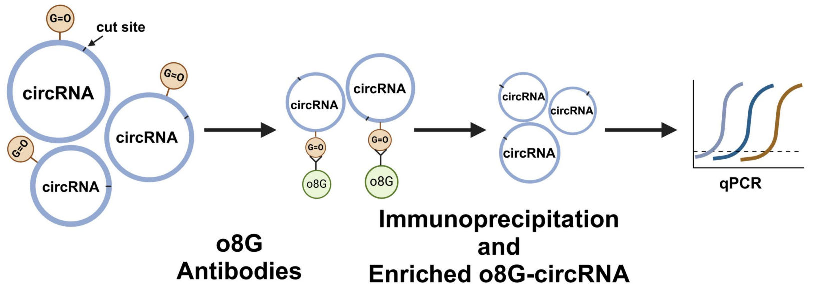


C

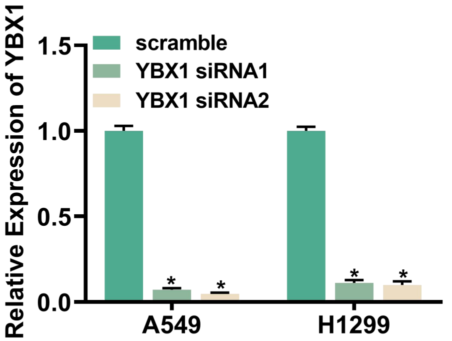


B

| Protein ID | RNA ID | Interaction Propensity | Z-score | Interaction Matrix | RBP propensity | RNA-Binding Domains | RNA-Binding Motifs | Conserved Interactions | Ranking |
|---|---|---|---|---|---|---|---|---|---|
| YBX1 | circKIAA1797 | 8.92 | -0.21 | table\|plot | 1 | 1 | 2 | 0/0 | |

| Protein ID | RNA ID | Interaction Propensity | Z-score | Interaction Matrix | RBP propensity | RNA-Binding Domains | RNA-Binding Motifs | Conserved Interactions | Ranking |
|---|---|---|---|---|---|---|---|---|---|
| AUF1 | circKIAA1797 | 6.65 | -0.35 | table\|plot | 1 | 5 | 0 | 0/0 | |

E

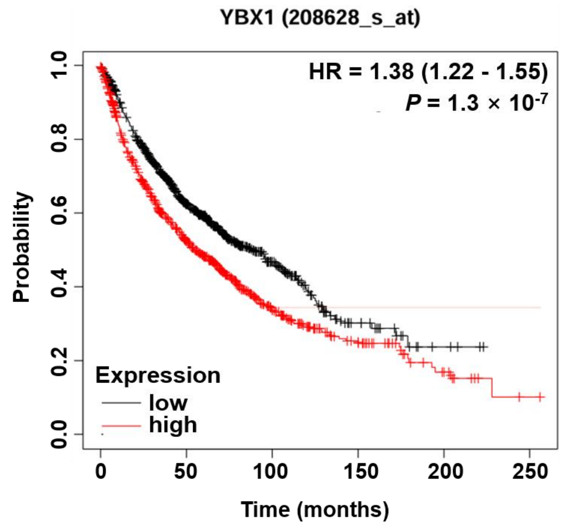


D

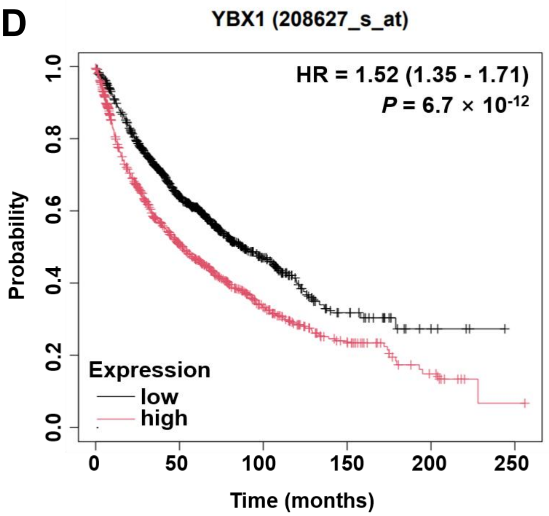


F

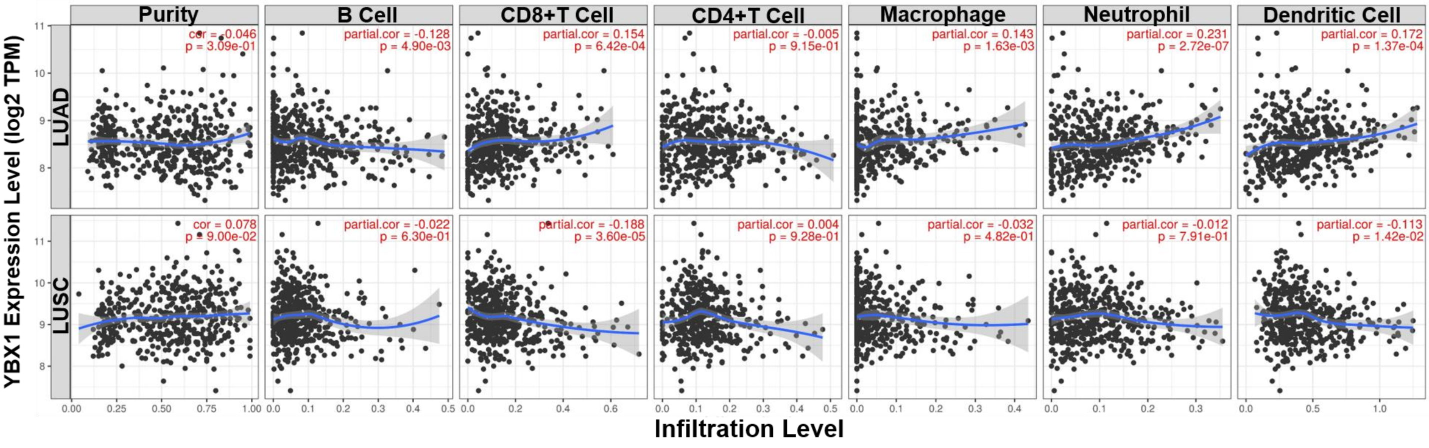

## Supplementary Figure 3

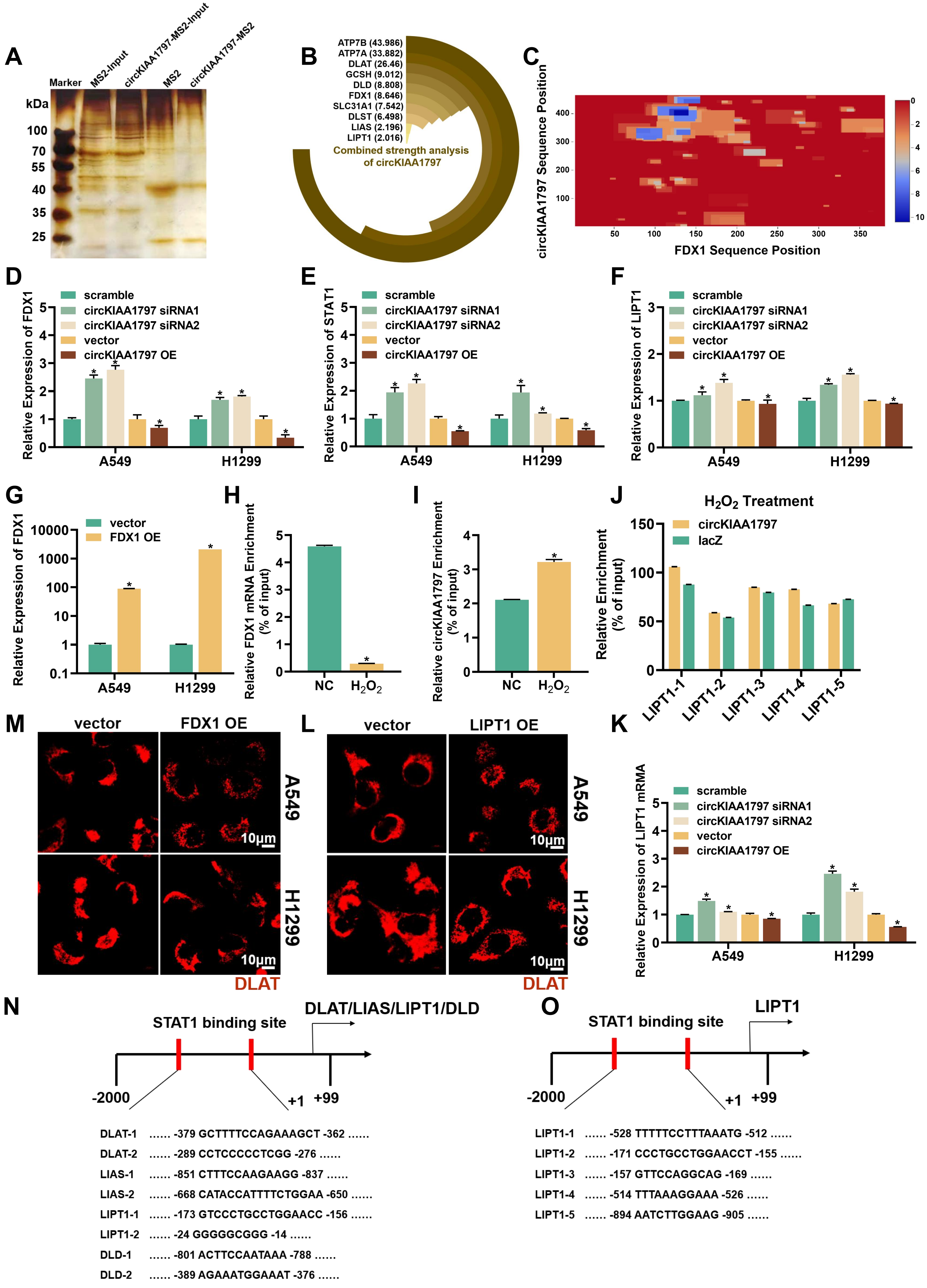

# Supplementary Figure 4

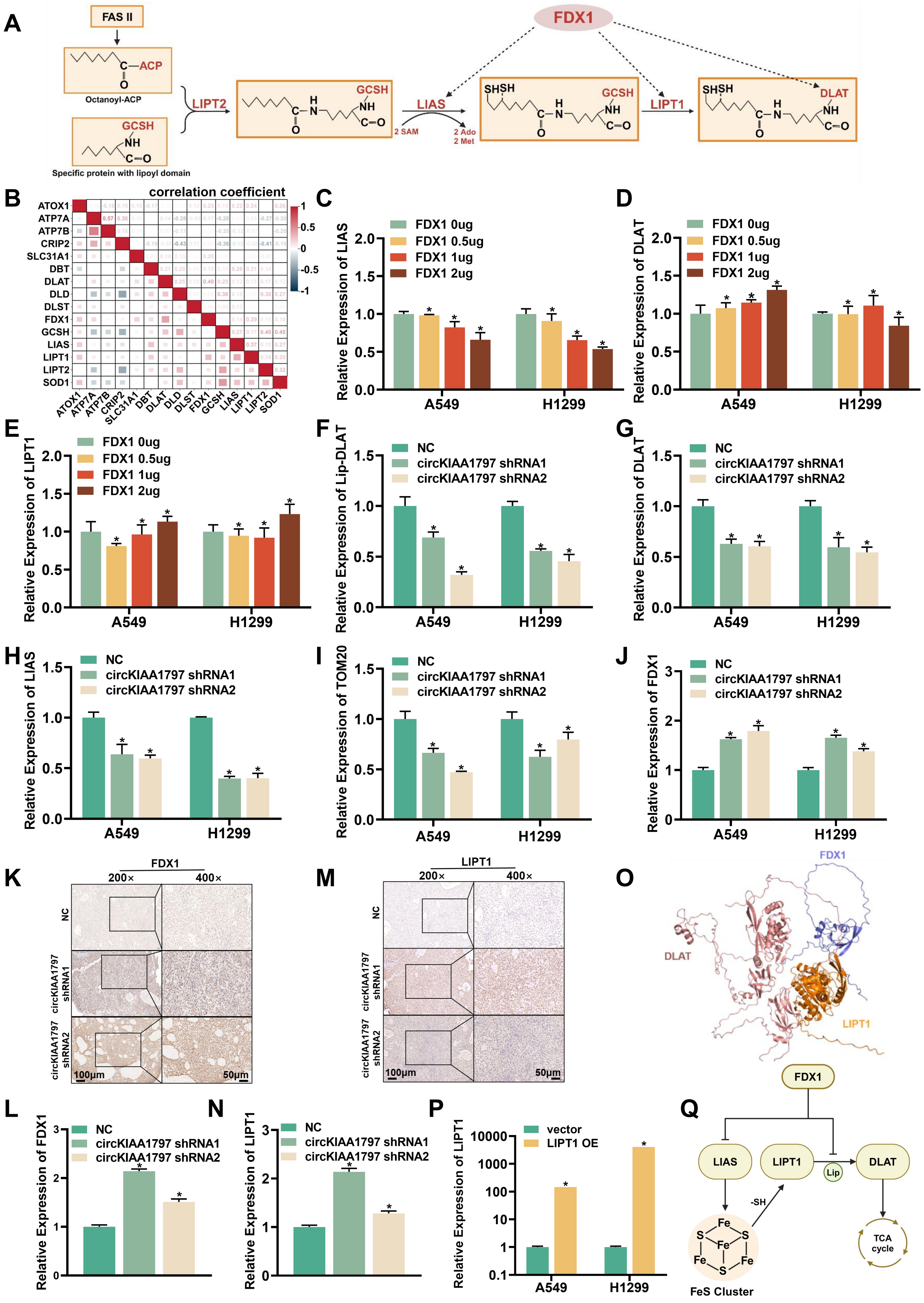

# Supplementary Figure 5

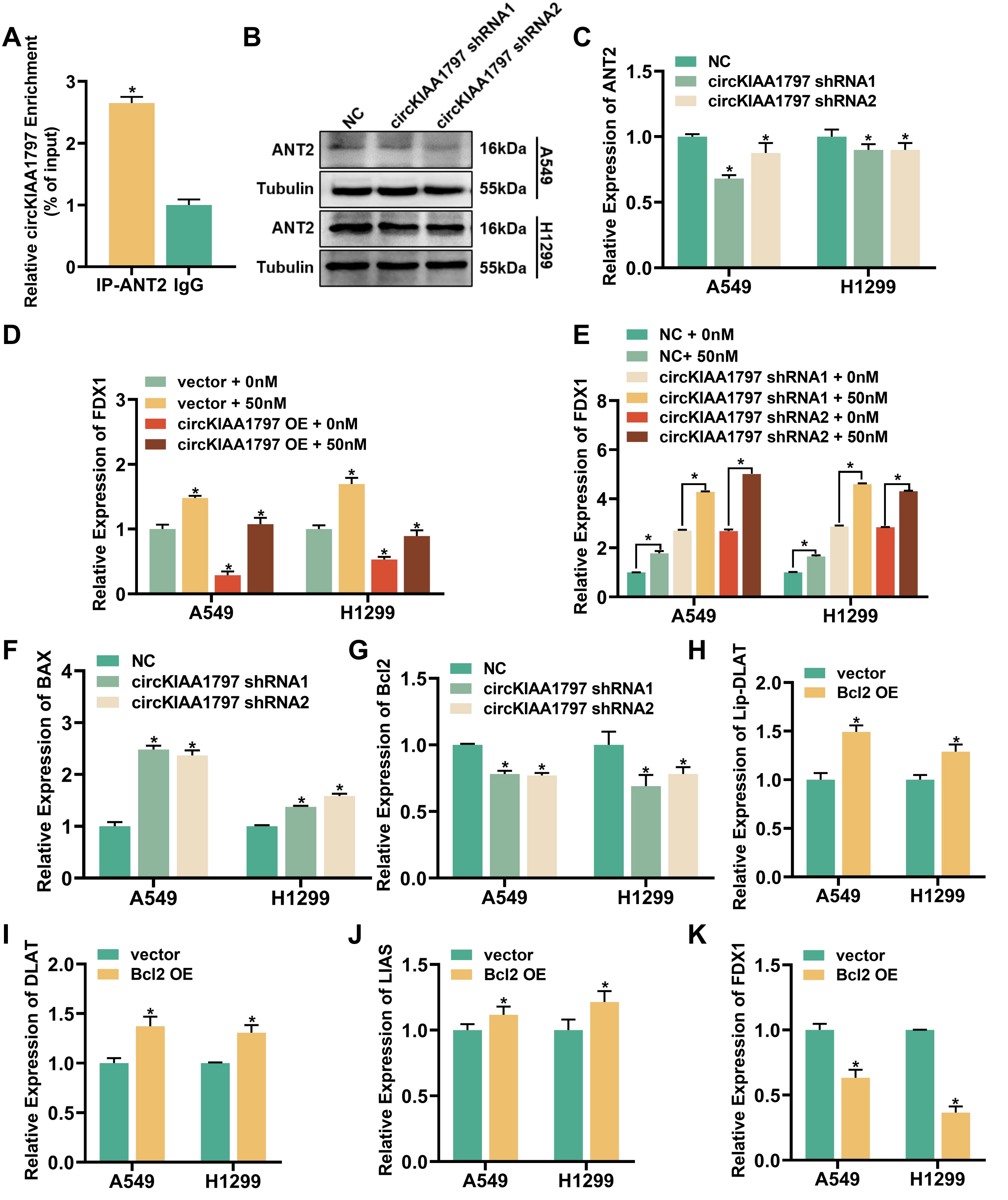

# Supplementary Figure 6

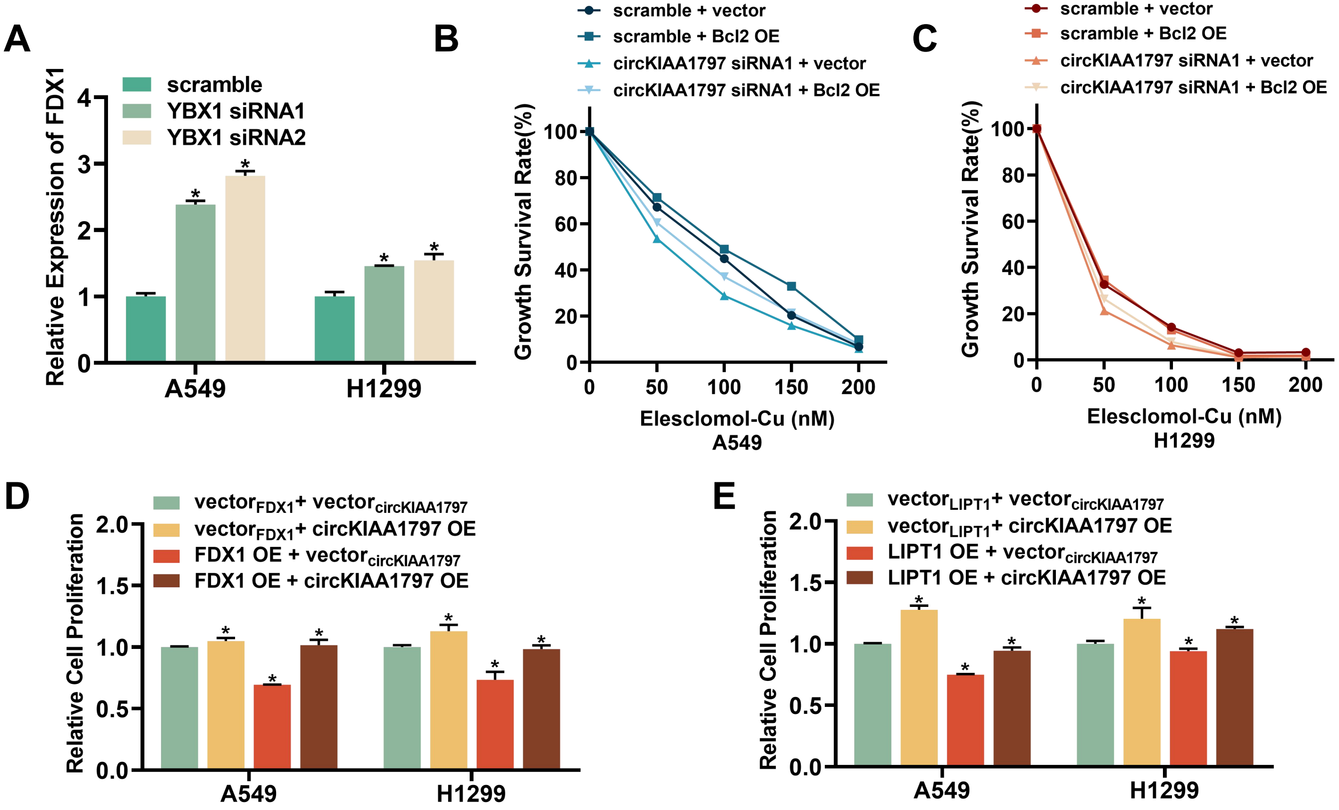

Supplement: Supplementary file 6 — Supplementary Material 6 [file 13046_2025_3365_MOESM6_ESM.pdf]
