# Supplementary material for: o8G-modified circKIAA1797 promotes lung cancer development by inhibiting cuproptosis
Source: J Exp Clin Cancer Res. 2025 Apr 2;44:110. doi: 10.1186/s13046-025-03365-z (PMC11963662; doi:10.1186/s13046-025-03365-z)
Supplement: Supplementary file 7 — Supplementary Material 7 [file 13046_2025_3365_MOESM7_ESM.docx]

**Supplementary figure legends**

**Supplementary Fig. 1**

**Gene screening of circKIAA1797.** (A) Detailed information on the top 10 circRNAs that were significantly upregulated in high-throughput sequencing. (B) Correlation analysis of the smoking status with circKIAA1797 expression in 35 pairs of tissues from lung cancer patients. (C) Correlation analysis of the tumour stage with circKIAA1797 expression in 35 pairs of tissues from lung cancer patients. (D) The FISH results of circKIAA1797 after RNase R treatment.

**Supplementary Fig. 2**

**Bioinformatics analysis of YBX1.** (A) Schematic diagram of the o8G RIP experiment. (B) Results of the *cat*RAPID web server prediction of the binding affinity of circKIAA1797 to YBX1 and AUF1, respectively. (C) YBX1 silencing efficiency was detected by qPCR after the transient silencing of YBX1 in A549 and H1299 cells. (D and E) The Kaplan-Meier plot website was used to predict the OS survival curve based on the expression of YBX1 in lung cancer samples (datasets 208628 and 208627). (F) Results of the immune cell infiltration analysis of YBX1 in lung cancer via the TIMER website.

**Supplementary Fig. 3**

**circKIAA1797 regulates the expression of the cuproptosis-related proteins FDX1** **and LIPT1**. (A) Results of TRAP experiments and silver staining. (B) Analysis of the possibility of circKIAA1797 binding to 10 cuproptosis-related proteins. (C) Volcano plot of the predicted results showing the direct binding of circKIAA1797 to the FDX1 mRNA. (D to F) Results of the grayscale value analysis of the Western blot bands representing FDX1, STAT1, and LIPT1 protein expression after the transient silencing and overexpression of circKIAA1797. (G) The efficiency of transient FDX1 overexpression in A549 and H1299 cells was detected by qPCR. (H) ChIRP–qPCR results showing the direct binding of circKIAA1797 to the FDX1 mRNA after the cells were treated with H_2_O_2_. (I) Cells were treated with H_2_O_2_, and the ability of circKIAA1797 to bind to the STAT1 protein was detected by RIP‒qPCR. (J) ChIP‒qPCR results from cells treated with H_2_O_2_ showing the ability of circKIAA1797 to bind to the LIPT1 promoter region. (K) qPCR detection of LIPT1 mRNA expression after the transient silencing and overexpression of circKIAA1797 in A549 and H1299 cells. (L) IF assay showing the extent of DLAT oligomerization after transient overexpression of LIPT1 in A549 and H1299 cells. (M) The degree of DLAT oligomerization was detected by IF staining after FDX1 was transiently overexpressed in A549 and H1299 cells. (N) Schematic diagram of the predicted binding sites of the transcription factor STAT1 to the promoter regions of the DLAT, LIAS, LIPT1, and DLD genes. (O) Schematic diagram of the predicted binding sites of the transcription factor STAT1 to the promoter region of the LIPT1 gene.

**Supplementary Fig. 4**

**Exploration of the mechanisms underlying cuproptosis induction.** (A) Schematic diagram of the lipid-based transfer process. (B) Correlation analysis of 15 cuproptosis-related proteins (ATOX1, ATP7A, ATP7B, CRIP2, SLC31A1, DBT, DLAT, DLD, DLST, FDX1, GCSH, LIAS, LIPT1, LIPT2, and SOD1). (C to E) Gradient overexpression of FDX1 followed by Western blot detection of the LIAS, LIPT1, and DLAT proteins and grayscale value analysis. (F to J) Analysis of Western blot band grayscale values for the protein expression of Lip-DLAT, DLAT, LIAS, TOM20, and FDX1 in cells with stable circKIAA1797 knockdown. (K to N) Immunohistochemistry was performed to detect the protein expression of FDX1 and LIPT1 in nude mouse tumours, and the results were quantified. (O) Schematic diagram of HADDOCK predictions of FDX1, LIPT1 and DLAT binding. (P) Validation of the LIPT1 overexpression efficiency detected by qPCR in A549 and H1299 cells. (Q) Schematic diagram of the FDX1 regulatory mechanism.

**Supplementary Fig. 5**

**circKIAA1797 inhibits cuproptosis by promoting mPTP closure.** (A) RIP-qPCR results showing that circKIAA1797 binds to ANT2. (B and C) Western blot analysis of ANT2 protein expression and grayscale value analysis after the stable silencing of circKIAA1797. (D) After the transient overexpression of circKIAA1797, 50 nM elesclomol-Cu was added, and Western blot was performed to detect FDX1 protein expression and grayscale values were analysed. (E) Grayscale values of FDX1 protein expression detected by Western blot in cells stably transfected with circKIAA1797 and treated with 50 nM elesclomol-Cu. (F and G) Analysis of BAX versus Bcl2 protein expression according to the grayscale values of the Western blot bands in cells with stable circKIAA1797 silencing. (H to K) Grayscale values of Western blot bands representing Lip-DLAT, DLAT, LIAS and FDX1 protein expression after the overexpression of Bcl2.

**Supplementary Fig. 6**

**circKIAA1797 promotes lung cancer development by inhibiting cuproptosis.** (A) Grayscale values of the protein expression of FDX1 represented by Western blot bands after the transient silencing of YBX1. (B and C) After the simultaneous silencing of circKIAA1797 and overexpression of Bcl2, a CCK-8 assay was performed to detect cellular resistance to cuproptosis. (D) Results of the EdU assay used to detect changes in cell proliferation after the simultaneous overexpression of FDX1 and circKIAA1797. (E) Results of the EdU incorporation assay showing changes in cell proliferation after the simultaneous overexpression of LIPT1 and circKIAA1797.
